# Overture: an open-source genomics data platform

**DOI:** 10.1093/gigascience/giaf038

**Published:** 2025-04-24

**Authors:** Mitchell Shiell, Rosi Bajari, Dusan Andric, Jon Eubank, Brandon F Chan, Anders J Richardsson, Azher Ali, Bashar Allabadi, Yelizar Alturmessov, Jared Baker, Ann Catton, Kim Cullion, Daniel DeMaria, Patrick Dos Santos, Henrich Feher, Francois Gerthoffert, Minh Ha, Robin A Haw, Atul Kachru, Alexandru Lepsa, Alexis Li, Rakesh N Mistry, Hardeep K Nahal-Bose, Aleksandra Pejovic, Samantha Rich, Leonardo Rivera, Ciarán Schütte, Edmund Su, Robert Tisma, Jaser Uddin, Chang Wang, Alex N Wilmer, Linda Xiang, Junjun Zhang, Lincoln D Stein, Vincent Ferretti, Mélanie Courtot, Christina K Yung

**Affiliations:** Ontario Institute for Cancer Research (OICR), Ontario, Canada, M5G 1M1; Ontario Institute for Cancer Research (OICR), Ontario, Canada, M5G 1M1; Ontario Institute for Cancer Research (OICR), Ontario, Canada, M5G 1M1; Ontario Institute for Cancer Research (OICR), Ontario, Canada, M5G 1M1; Ontario Institute for Cancer Research (OICR), Ontario, Canada, M5G 1M1; Ontario Institute for Cancer Research (OICR), Ontario, Canada, M5G 1M1; Ontario Institute for Cancer Research (OICR), Ontario, Canada, M5G 1M1; Ontario Institute for Cancer Research (OICR), Ontario, Canada, M5G 1M1; Ontario Institute for Cancer Research (OICR), Ontario, Canada, M5G 1M1; Ontario Institute for Cancer Research (OICR), Ontario, Canada, M5G 1M1; Ontario Institute for Cancer Research (OICR), Ontario, Canada, M5G 1M1; Ontario Institute for Cancer Research (OICR), Ontario, Canada, M5G 1M1; Ontario Institute for Cancer Research (OICR), Ontario, Canada, M5G 1M1; Ontario Institute for Cancer Research (OICR), Ontario, Canada, M5G 1M1; Ontario Institute for Cancer Research (OICR), Ontario, Canada, M5G 1M1; Ontario Institute for Cancer Research (OICR), Ontario, Canada, M5G 1M1; Ontario Institute for Cancer Research (OICR), Ontario, Canada, M5G 1M1; Ontario Institute for Cancer Research (OICR), Ontario, Canada, M5G 1M1; Ontario Institute for Cancer Research (OICR), Ontario, Canada, M5G 1M1; Ontario Institute for Cancer Research (OICR), Ontario, Canada, M5G 1M1; Ontario Institute for Cancer Research (OICR), Ontario, Canada, M5G 1M1; Ontario Institute for Cancer Research (OICR), Ontario, Canada, M5G 1M1; Ontario Institute for Cancer Research (OICR), Ontario, Canada, M5G 1M1; Ontario Institute for Cancer Research (OICR), Ontario, Canada, M5G 1M1; Ontario Institute for Cancer Research (OICR), Ontario, Canada, M5G 1M1; Ontario Institute for Cancer Research (OICR), Ontario, Canada, M5G 1M1; Ontario Institute for Cancer Research (OICR), Ontario, Canada, M5G 1M1; Ontario Institute for Cancer Research (OICR), Ontario, Canada, M5G 1M1; Ontario Institute for Cancer Research (OICR), Ontario, Canada, M5G 1M1; Ontario Institute for Cancer Research (OICR), Ontario, Canada, M5G 1M1; Ontario Institute for Cancer Research (OICR), Ontario, Canada, M5G 1M1; Ontario Institute for Cancer Research (OICR), Ontario, Canada, M5G 1M1; Ontario Institute for Cancer Research (OICR), Ontario, Canada, M5G 1M1; Ontario Institute for Cancer Research (OICR), Ontario, Canada, M5G 1M1; Ontario Institute for Cancer Research (OICR), Ontario, Canada, M5G 1M1; Department of Molecular Genetics, University of Toronto, Toronto, Canada, M5S 3K3; Ontario Institute for Cancer Research (OICR), Ontario, Canada, M5G 1M1; Research Center of the CHU Sainte-Justine, University of Montreal, Montreal, Canada, QC H3T 1C5; Ontario Institute for Cancer Research (OICR), Ontario, Canada, M5G 1M1; Department of Medical Biophysics, University of Toronto, Toronto, Canada, M5G 2C4; Ontario Institute for Cancer Research (OICR), Ontario, Canada, M5G 1M1

**Keywords:** research software, data management, genomics, open-source, open-science

## Abstract

**Background:**

Next-generation sequencing has created many new technological challenges in organizing and distributing genomics datasets, which now can routinely reach petabyte scales. Coupled with data-hungry artificial intelligence and machine learning applications, findable, accessible, interoperable, and reusable genomics datasets have never been more valuable. While major archives like the Genomics Data Commons, Sequence Reads Archive, and European Genome-Phenome Archive have improved researchers’ ability to share and reuse data, and general-purpose repositories such as Zenodo and Figshare provide valuable platforms for research data publication, the diversity of genomics research precludes any one-size-fits-all approach. In many cases, bespoke solutions are required, and despite funding agencies and journals increasingly mandating reusable data practices, researchers still lack the technical support needed to meet the multifaceted challenges of data reuse.

**Findings:**

Overture bridges this gap by providing open-source software for building and deploying customizable genomics data platforms. Its architecture consists of modular microservices, each of which is generalized with narrow responsibilities that together combine to create complete data management systems. These systems enable researchers to organize, share, and explore their genomics data at any scale. Through Overture, researchers can connect their data to both humans and machines, fostering reproducibility and enabling new insights through controlled data sharing and reuse.

**Conclusions:**

By making these tools freely available, we can accelerate the development of reliable genomic data management across the research community quickly, flexibly, and at multiple scales. Overture is an open-source project licensed under AGPLv3.0 with all source code publicly available from https://github.com/overture-stack and documentation on development, deployment, and usage available from www.overture.bio.

## Background

Genomics research has benefited from a strong tradition of open-science principles that have fostered comprehensive studies, transparent results, and accelerated scientific discovery [1]. As sequencing costs decrease, large and small research groups are increasingly generating massive multiomics and single-cell data sets [2], often combined with clinical and imaging data [3]. This data abundance coincides with the emergence of machine learning (ML) and artificial intelligence (AI) [4], the biggest data-consuming activities in history. These emerging fields have sprouted from the wide availability of data; however, their solutions are limited by the quality of relevant data openly available for consumption [5]. Our brave new world now demands readily available software infrastructure to collect, organize, and share data.

In response, funding agencies and academic journals increasingly insist that projects generating large amounts of sequencing data respect FAIR (Findable, Accessible, Interoperable and Reusable) data practice [5]. This shift reflects a growing recognition and expectation of the researcher’s role in facilitating data reuse. However, sharing genomics data is a multifaceted challenge:

- The volume of data often requires researchers to use cloud-based solutions that introduce new costs and expertise [6].
- Sharing data across the research community must be done in an interoperable and sustainable fashion [7].
- The legal, ethical, and social implications of genomics data sharing, including ownership, sovereignty, and data misuse, are extensive and evolving [8, 9].

While several resources for depositing genomic data, such as the Genomic Data Commons (GDC) [10], Sequence Read Archive (SRA) [11], and European Genome-Phenome Archive (EGA) [12], take on much of the responsibility of managing and archiving data, not every project is eligible to submit data to these repositories. The GDC focuses on human cancer genomes and is limited to DNA and RNA sequencing data in FASTQ and BAM formats [13]. The EGA only accepts human genomic and phenotypic data [14]. Alternatively, the SRA accepts a wide range of sequencing data types and is agnostic to the organism of origin [15]. However, like all archival resources, it faces fundamental challenges in accommodating niche and rapidly evolving data requirements [16]. This presents a serious challenge to research groups with large volumes of data that do not meet the requirements of existing archival resources, and projects with datasets that do not conform to the specific data model and file types accepted by these repositories get left out. Instead, these results must be shared *ad hoc*, such as in publication supplementary data files. To meet FAIR standards, genomic data must be maintained in an online system that allows for search and retrieval, is well structured, and supports metadata and provenance tracking. Few research groups have the expertise to implement such a system [17], leaving groups with the choice of building the expertise in-house or hiring outside consultants, developers, and IT support staff. The first solution is inefficient, and the second one frequently exceeds available funding.

We created Overture [18, 19] to enable researchers to build and deploy reproducible large-scale data platforms. With these platforms, researchers can maximize the potential of existing research, encouraging transparency, reproducibility, and reuse of data while maintaining oversight over its distribution. Other researchers can see their results, explore the data underpinning them, and reuse them to drive further discovery.

## Results

### Development of Overture

Overture was built based on a data portal and submission system developed to support the Data Coordination Center of the International Cancer Genome Consortium (ICGC-DCC) [20, 21], a popular cancer genomics resource covering 84 worldwide projects, and molecular data from over 24,000 patients. The ICGC-DCC platform provided researchers with a user-friendly interface for efficient access, visualization, and analysis of its genomic data. After its launch, the portal’s data exploration and analysis capabilities attracted attention from various research groups. Several of these groups successfully reimplemented and adapted the ICGC-DCC’s infrastructure, including the Hartwig Medical Database [22] and the Translational Human Pancreatic Islet Genotype Tissue-Expression Resource Data Portal (TIGER) [23].

However, the reusability of the ICGC-DCC infrastructure was met with significant technical challenges, particularly tied to its monolithic architecture and numerous hard-coded elements, which made it unnecessarily difficult to replicate and implement the system in projects with similar needs and limited the ability to scale in production. These limitations prompted a strategic shift toward a more flexible and scalable microservice architecture. This was chosen for several key advantages. (i) *Scalability:* microservices enable individual system components to scale independently. (ii) *Flexibility:* each microservice can be deployed and upgraded separately, easing the development of new features and modifications to existing ones. (iii) *Resilience:* if one microservice encounters a failure, other instances reduce or even avert downtime by load balancing accordingly. This resulted in the development of Overture, a collection of reusable and modular microservices that serve as a general solution for building and deploying data platforms.

### Platform overview

**Figure 1:**
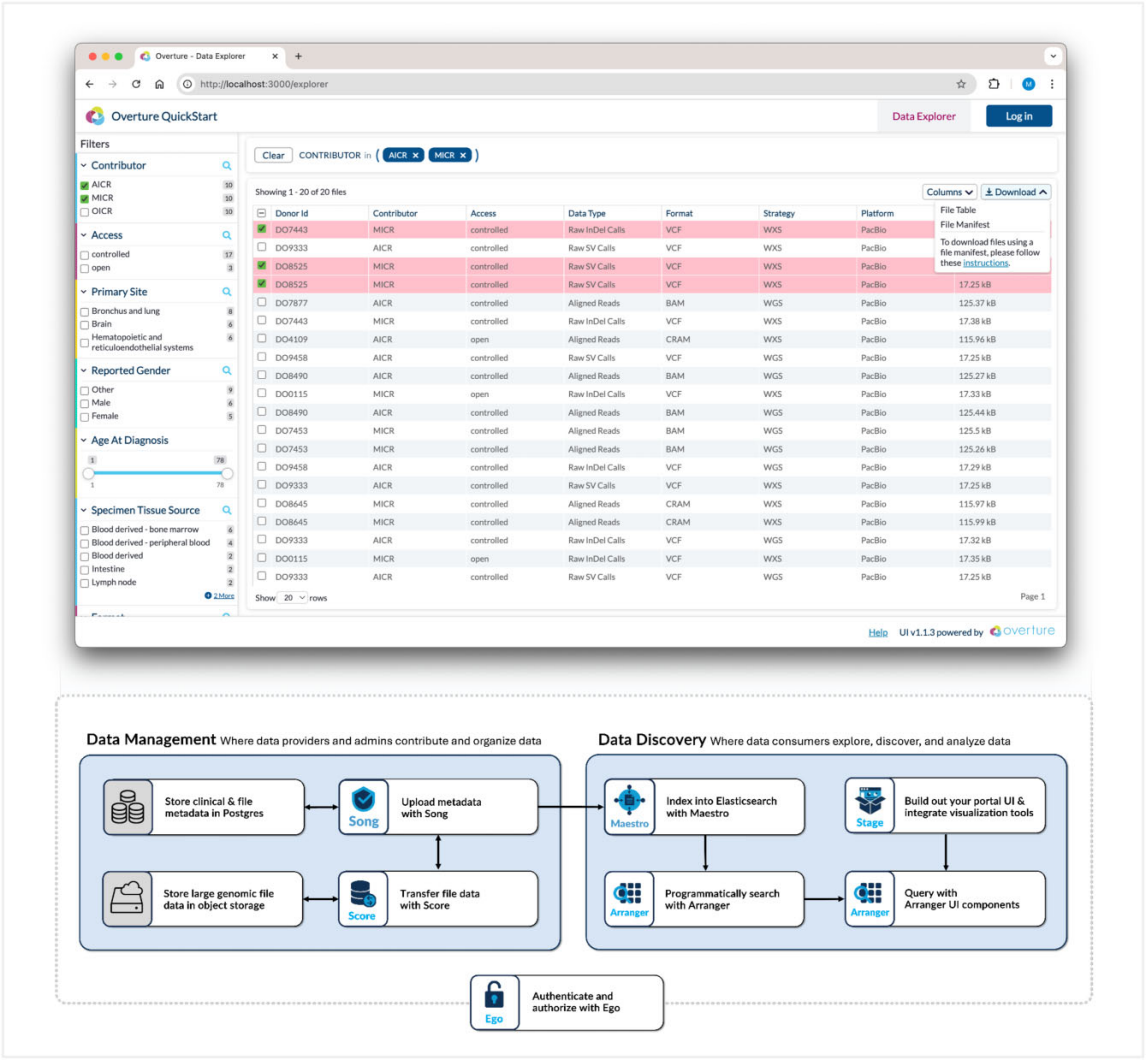
Platform components overview: on the front-end, Stage [24] provides the basic user interface (UI), including navigation menus, as well as data exploration, login, and profile pages. Arranger’s [25] library of search UI components then integrates with Stage to offer a configurable search facet panel, data table, and filter summary panel. Login and profile pages integrate with Keycloak [26] or Ego [27], which provides authentication and authorization for users and applications. Behind the scenes, Song [28] and Score [29] facilitate data management, retrieval, and submission. Score transfers large genomic files to and from S3-compatible object storage, while Song stores and handles the files’ metadata. These databases are indexed by Maestro [30] into unified Elasticsearch [31] file-centric and analysis-centric indices. Arranger then uses these to produce a GraphQL [32] search API that connects with its front-end library components on the data exploration page. Combined together, these services broadly enable the secure and scalable reuse of genomics data.

The Overture platform is highly flexible yet fundamentally has a standard feature set provided by its core software components (Fig. 1 and Table 1 ). The target users for our data platform’s core functionalities can be divided into 3 categories:

1. Data consumers retrieving data from the platform
2. Data providers submitting data to the platform
3. Data administrators who configure and maintain the platform

**Table 1:** Overview of Overture stack software components: Overture comprises 6 core components and 1 third-party component that work in concert to create genomics data management systems

Product name	Code repository	Brief description
*Song*	https://github.com/overture-stack/song	Metadata management with an automated submission validation system.
*Score*	https://github.com/overture-stack/score	File transfer microservice that supports fault-tolerant multipart parallel transfer.
*Maestro*	https://github.com/overture-stack/maestro	Indexes metadata from Song into Elasticsearch search indices, to be consumed by Arranger.
*Arranger*	https://github.com/overture-stack/arranger	Data search and exploration API and accompanying library of UI components that can be easily integrated in a data portal.
*Ego*	https://github.com/overture-stack/ego	OAuth 2.0 authorization service that supports multiple OpenID Connect (OIDC) identity providers.
*Keycloak*	https://github.com/keycloak/keycloak	A popular third-party open-source identity and access management service.
*Stage*	https://github.com/overture-stack/Stage	A React-based user interface designed to allow easy deployment of browser-friendly data portals.

### Data retrieval

**Figure 2:**
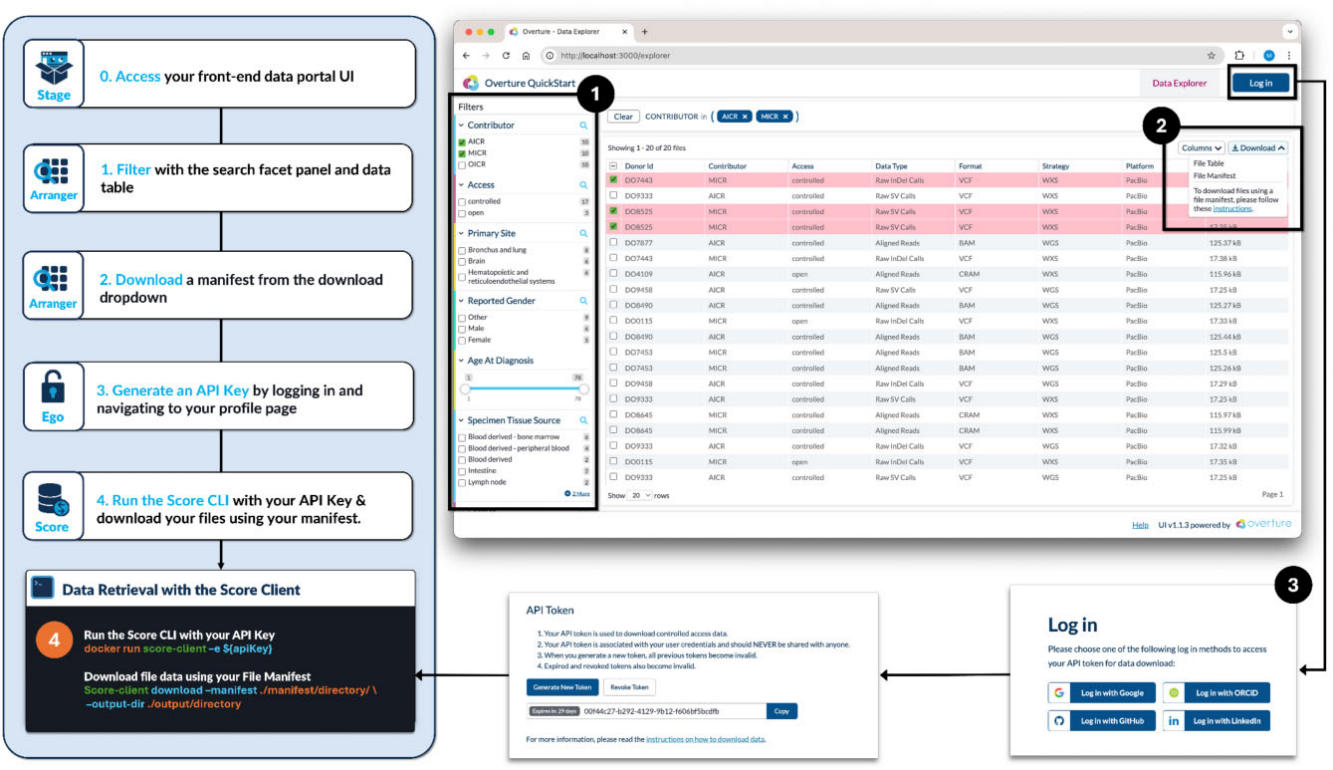
Data retrieval workflow: users first filter data via Arranger’s search components in the Stage UI’s data explorer. Once they have selected a subset, they then download a “file manifest” from the download dropdown. To access Song and Score data, users log in through Stage’s auth integration and obtain their API key from the profile page. This API key is provided when installing the score client. Finally, files are downloaded to the user’s device using the Score client’s download command, specifying the file manifest and desired output directory.

Data retrieval starts from the Stage data exploration page (Fig. 2), where users can filter data using the Arranger search facets. These enable rapid and efficient data filtering using checkboxes, date ranges, sliders, and quick search input boxes, allowing users to narrow their queries and focus on relevant data subsets. Filtered datasets are presented in the Arranger data table, which provides sortable columns, file counts, and pagination. All query parameters are summarized within an Arranger filter panel at the top of the page, giving users a clear overview of their search criteria. Users can easily share these queries using the browser URL, which gets updated with the filter parameters in real time.

Once the users have identified relevant data, they can select the download dropdown, which provides options for downloading metadata or a file manifest in a TSV format. The manifest file allows users to download their files of interest directly from the resources database and object storage using Overture’s command line interface (CLI) tools, specifically the Song and Score clients. These CLI tools are needed as massive genomic datasets require reliable multipart parallel download sessions unsuitable for a browser. To ensure secure access to data, users must supply a valid API key when installing the Song and Score clients. For controlled-access data, researchers will be able to retrieve their API key after their data access request is granted by typically the relevant data access committee. Log-in to the web portal is facilitated by either Keycloak or Ego, which supports popular identity providers, including Google, ORCiD, and GitHub.

### Data submission

**Figure 3:**
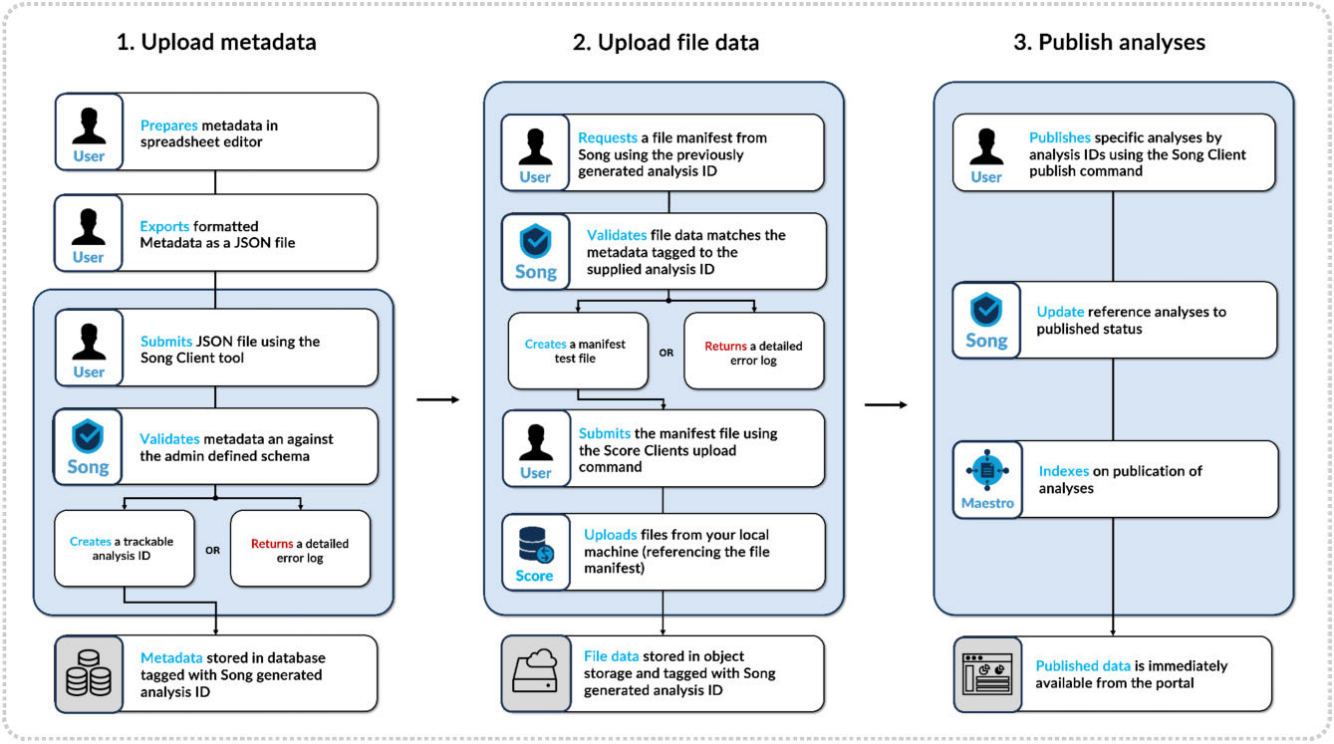
Data submission workflow: Overture’s submission process enhances data integrity with data tracking and data model adherence. It involves organizing metadata files, converting them to JSON, and uploading via the Song Client for validation. Successful submissions receive an auto-generated analysis ID. File data are then uploaded using Song and Score clients, generating a file manifest linked to the metadata. All data start unpublished and are managed through Song’s publication controls for coordinated data releases.

Overture’s submission process has been designed to ensure data integrity by facilitating data tracking and data model compliance (Fig. 3). In Overture, a set of 1 or more files plus the metadata describing that collection of files is called an analysis. To upload an analysis, data submitters first organize their metadata files, typically using a spreadsheet editor alongside a data dictionary supplied by the resource administrator. To provide a unified view of the metadata in the Overture exploration table, submitted data must be curated against the chosen metadata model. Alternatively, users can decide to create multiple exploration tables for independent and heterogeneous datasets. The data dictionary describes the required metadata fields and the expectations for the syntax of each field. Once converted to JSON, the Song Client upload command can be used to send the metadata submission to the resources Song server for validation against the admin-defined data model. If there are any issues with the metadata, the user will be provided a detailed error message. If successful, the user will be provided a success message and an auto-generated analysis ID for future reference within the system.

After submitting metadata and establishing an analysis ID, the submitter uploads file data using Song and Score clients. First, a file manifest is generated using the Song client manifest command, along with the directory where the files are located and the analysis ID assigned to the relevant metadata. This links the uploaded files to the metadata in Song’s database. The manifest and Score Client upload command is then used to transfer files to object storage. Once uploaded, Song can dynamically update file metadata, such as md5 checksums, to the appropriate analysis file within its database.

All data uploaded to the resource are, by default, in an unpublished state. Publication controls allow administrators and data providers to coordinate and prepare data releases in a predictable and timely manner. All publication controls are facilitated by Song using its publication command or endpoint. When data are ready for search and download, administrators can make them available by updating the desired analyses to a published state. If data are no longer relevant, the data administrators can make them unavailable to downstream services by setting analyses to a suppressed state.

### Data administration

Data administrators are responsible for maintaining and configuring the data resource, including providing the data model, configuring the portal search interface, and managing users’ access and permissions. With Overture, administrators have the flexibility to define the data model of their resources according to their desired standards. This involves specifying the structure and syntax of the data model in JSON format. Once the JSON schema is created, the administrator can submit it to Overture’s Song microservice through the Song API. This schema then serves to verify all future submissions to the resource. Multiple schemas can be registered to the platform, enabling validation for various data types.

Given the potential diversity of projects that can use Overture, administrators require flexibility in customizing their portal front-end interface. With Arranger’s library of UI components, among other customizations, administrators can configure the display settings of the search facets and data table on the portal exploration page. This includes configurations for what fields are visible, how they are displayed, and how users can interact with them.

Admins also have the flexibility to customize the portal’s content by building on top of the Stage UI. Stage is a React-based single-page web application designed to provide a foundation for building any data portal. Its default features include a header, a footer, and navigation menus. With functional knowledge of React, you can easily theme and extend Stage to include custom pages and menu options, such as funding acknowledgments, documentation, and data release statements.

Depending on the authorization service chosen, admins can manage user permissions through the Ego admin UI or KeyCloak. These allow admins to apply role-based permissions granting read or write access to users and applications through JSON Web Tokens (JWTs) or API Keys.

### Impact

The types of groups that can deploy Overture platforms can be categorized into 2 segments based on the scale of data and their level of in-house expertise:

1. **Large-scale consortiums** with tens to hundreds of thousands of samples and technical staff to support the expansion of both hardware and software infrastructure. They may also need to distribute data across multiple locations and jurisdictions.
2. **Medium- to small-sized labs, programs, and institutions** with hundreds to thousands of samples and limited system administration support.

We will address each of these groups in the following sections.

### Impact on consortium-level projects

Multiple consortium-level projects have successfully reused Overture components, reducing development efforts and, in turn, driving further enhancement. As a testament to the benefits of a modular approach, Overture has significantly impacted several consortium-level projects (Table 2).

**Table 2:** Impact on consortium-level projects: an overview of consortium-level data-sharing initiatives. Each initiative briefly describes the project and the Overture components that help drive it.

Project	Description	Component(s) used	Data
**Kids First Data Resource Portal** [34]	The Kids First Data Portal provides access to genomic and clinical datasets generated by the Kids First Pediatric Cancer, the Rare Diseases Data Resource, and other National Cancer Institute–supported pediatric genomics projects. The datasets are stored in a secure, centralized repository and made available worldwide to researchers and the public. For more information, see our case study page (https://www.overture.bio/case-studies/#kidsfirst).	Arranger	34,000 Human genomes
**Human Cancer Models Initiative (HCMI) Searchable Catalog** [35]	The HCMI catalogs cancer models alongside clinical, biospecimen, and molecular data. This data-sharing platform also includes protocols, informed consent templates, and clinical data forms, making it an all-in-one resource for translational cancer researchers. For more information, see our case study page (https://www.overture.bio/case-studies/#HCMI).	Arranger	307 Cancer models
**International Health Cohorts Consortium (IHCC) Cohort Atlas** [36]	The IHCC is improving clinical care and population health by aggregating large genomic data cohorts to help translational researchers uncover the biological and genetic factors of disease. The IHCC Cohort Atlas is the global data-sharing platform hosting genomics data from large cohorts (100k+). **Using a single data model unifies international cohort data and enables discovery through Arranger’s search functionalities**. For more information, see our case study page (https://www.overture.bio/case-studies/#IHCC).	Arranger	Human cohort metadata for 34 million participants
**International Cancer Genome Consortium–Accelerating Research in Genomic Oncology (ICGC-ARGO)** [37]	ICGC-ARGO is a global initiative to provide precision oncology knowledge to the world. With the goal of analyzing genomes from 100,000 patients with cancer, ICGC-ARGO aims to collect genomic data alongside high-quality clinical data and make them available to the research community in a rapid and responsible way. **(Re)use and extension of Overture components supports controlled data storage and access**. For more information, see our case study page (https://www.overture.bio/case-studies/#ARGO).	Ego, Song, Score, Maestro, Arranger	37,222 Genomic files
**VirusSeq Data Portal** [38, 39]	The VirusSeq Data Portal is an open-source and open-access data portal for all Canadian SARS-CoV-2 sequences as well as associated nonpersonal contextual data. It harmonizes, validates, and automates submission to international databases. **Using Overture, the portal was created within a 4-week time frame**. Initially intended to store 150,000 sequences, it has scaled to host over 500,000 genomes. For more information, see our case study page (https://www.overture.bio/case-studies/#virusseq).	Ego, Song, Score, Maestro, Arranger	98,266 Genomic files
**European-Canadian Cancer Network (EUCANCan)** [40]	The EUCANCan project offers a novel solution to managing and sharing cancer genomic data. **Overture’s Maestro enables federated search across 3 EUCANCan nodes**. Instead of consolidating data in one control center following some established process and timeline, each data node manages their own data locally. The nodes agree on the set of metadata that can be queried in a unified data portal that will then point to the location of the genomic data.	Ego, Song, Score, Maestro, Arranger	N/A
**Ontario Hereditary Cancer Research Network (OHCRN)** [41]	OHCRN aims to harmonize information from individuals with hereditary cancer syndrome in order to better understand and advance the prevention, early detection, and treatment of these cancers.	Arranger	N/A
**African Pathogen Data Sharing Archive (APA)** [42]	This data-sharing platform is being developed to enable real-time pathogen genomics sharing and exchange across Africa [43]. The portal allows users to upload, share, explore, and download pathogen sequences and associated metadata as per data use guidelines provided by each country. **Reusing Overture has enabled low and middle income countries' institutions to build local capacity and deploy their own platform**.	Ego, Song, Score, Maestro, Arranger	N/A

#### Case study: ICGC-ARGO

ICGC Accelerating Research in Genomic Oncology (ARGO) [33] is a global initiative to provide precision oncology knowledge. Intending to analyze genomes from 100,000 patients with cancer, ICGC-ARGO aims to collect genomic data alongside high-quality clinical data and make them available to the research community quickly and responsibly.

ICGC-ARGO operates at a global scale. To satisfy the legal requirements of data sovereignty, ARGO needs to implement a distributed network of servers or nodes located within each country of data origin. Thanks to Overture, the ARGO development team is deploying a global network of interoperable regional data-processing centers (RDPCs) where data are submitted and stored within each country of origin. Each RDPC leverages the core Overture components—Song, Score, and Maestro. The ARGO platform then federates queries through an Arranger server, enabling search across the global network of RDPC nodes from the ARGO Data Platform (https://platform.icgc-argo.org/).

### Medium to small laboratories and institutions

Overture has demonstrated its efficacy for large-scale genomics data platforms, with successful deployments across many projects. However, it is important to acknowledge that the projects presented so far only include consortium-level projects. While microservice architectures offer numerous advantages, the technical complexity of deploying our platforms has been a significant barrier for small- and medium-sized groups. To lower adoption barriers, we have identified and addressed 3 fundamental questions: How can potential new users see our platform in action? How can they openly experiment with the platform? And how can they take ownership of it?

To address the first question, we developed an Overture demo portal (https://demo.overture.bio/). This environment is accessible directly from our homepage and offers new and prospective users an immediate, interactive introduction to Overture’s capabilities. The demo features a representative mock dataset on the exploration page and includes supplementary content within the portal’s Stage UI, providing a surface-level overview of the platform’s functionality.

To facilitate open experimentation of our platform, we introduced the Overture Quickstart, a Docker Compose–based [43] makefile that enables users to deploy the entire platform locally within minutes, complete with prepopulated mock data and a preconfigured admin user. To accompany our localized Quickstart setup, we expanded our documentation to include platform guides that cover essential processes such as data submission, download, and core administrative tasks required for configuring an operational Overture platform.

To address ownership, we containerized and standardized the installation process for our microservices. Each service can now be installed using Docker and an environment variable file, ensuring broad portability across diverse computing environments. Furthermore, we now provide a comprehensive end-to-end deployment guide, which meticulously details each stage, service, and environment variable required for establishing a base Overture platform. Moving forward, we hope to provide guides and resources for automated deployments of a variety of ideal and reproducible environments leveraging popular toolings like Terraform [44] and Helm [45]. Through these initiatives, we hope to significantly reduce barriers to adoption, making Overture more accessible to a broader range of research groups, regardless of their scale or technical expertise.

## Discussion

As the software engineering team at the Ontario Institute for Cancer Research, we build data platforms with a diverse range of requirements. When our solutions prove widely applicable, they are refined into more generic tools and distributed as part of the Overture suite. In the following sections, we will discuss some of our current challenges and how they are guiding the expansion of the Overture suite.

Recent data protection laws, including the General Data Protection Regulation (EU GDPR) [46] and the Protection of Personal Information Act (POPIA) in South Africa [47], have created a shift in how we manage data across borders. Where data could formerly be transferred across jurisdictions, the current data protection laws prohibit this. Instead, we must host data in their geolocation of origin, deploying instances of the original platform in each country. This approach—*federation—*requires new means to discover the data at each node of the network instead of relying on centralized indexes. In response, we are improving our search API service Arranger, to aggregate search results across different nodes of arranger instances, enabling users to query datasets from other countries of origin while still maintaining the privacy of the individual. The extent to which the data can be aggregated and further explored centrally will need to be reviewed by ethics experts; our initial foray in the area has led to different interpretations and variable willingness to share data. Clear guidance from policy and legal experts will be required to achieve our vision of building a truly federated platform; we are tackling this through collaboration with the ICGC-ARGO ethical working group [48], as well as the GA4GH Regulatory & Ethics Work Stream (REWS) [49].

The management of patient consent and controlled access to data has become a standard requirement for large-scale human genomics platforms. In the past, to gain access to data, researchers were required to submit paper and PDF forms. This process can take months and has contributed to significant barriers to data access [7]. In response to challenges in controlled data access, we created an online application module for the ICGC-ARGO project. The Data Access Committee Office (DACO) application enables researchers to log in to an online portal, fill in and sign their application, and send it for review electronically to the Data Access Committee. The data access officer reviews applications through an online dashboard, through which they can request more information or approve/deny the applications. This process has reduced the average approval time from 4 weeks to 3.5 days for over 400 applications across 35 countries. For patient consent, we are building a virtual patient enrollment portal for the Ontario Hereditary Cancer Research Network. Designed to address real-world project demands, the patient consent portal enables study participants to provide consent and agree to online data sharing in both an ethical and accessible manner. Patient consent and controlled access to data are core requirements for platforms handling sensitive human data. Therefore, we are working to incorporate these 2 applications—the DACO system and the virtual patient enrollment portal—as new Overture components.

Overture’s development has been limited until recently for use on cancer genomics data. However, we are finding an increasing demand for projects that require data outside the context of cancer genomics [38, 39, 42]. In response, we are developing an updated data-agnostic tabular submission system to complement our existing infrastructure. This update allows us to cater to a broader range of use cases, such as pathogenic data, without additional development. Supporting additional data types from these diverse use cases introduces data heterogeneity challenges. Managing this is currently beyond the scope of Overture, and the data must be curated prior to submission for harmonized discovery against the chosen data model. We are investigating ways to semi-automate this mapping of datasets against the platform data model using large language models and natural language processing methodologies, and if successful, we will propose this as an added Overture module for data harmonization.

Our microservices architecture allows for flexibly adding and also retiring components when needed. For example, Ego is a bespoke Overture component to manage user authentication and authorization. Since its development, Keycloak has emerged as the industry standard open-source technology for authentication and authorization. Consequently, and while we currently support both Ego and Keycloak as described above, we are moving forward with deprecating Ego. This flexibility and ability to swap components means we can focus resources where needed, toward adding new functionalities and not duplicate work. As another example of this modularity, and in addition to modules described earlier for controlled access, data harmonization, and federation, our expanding user base requires better submissions support, in particular for nontechnical adopters. In response, we have started development of UI-based tools to improve platform usage and engagement, such as submission UIs, built-in documentation components, and a generic dictionary viewer. The Overture suite will continually evolve to address users’ needs—in terms of both software and documentation. Our newly developed quickstart resource has enabled us to establish a broader base of reference users. Based on their feedback, we are developing new resources to take them from planning to development and into production. These improvements to our onboarding experience will in turn promote further feedback from users, providing us the clarity and direction needed to drive greater success, adoption, and expansion of our software suite. Overture has successfully delivered on its goal to enable engineering teams to build and deploy reproducible large-scale data platforms that broadly enable FAIR data discovery and reuse. An upcoming focus of our work will be working toward making data platform development accessible to research teams with limited resources, reducing initial barriers, so teams can do more with less.

## Conclusions

The rapid expansion of genomics research and the intricate challenges of organizing and distributing its data present formidable obstacles for the field of genomics. Overture addresses this by offering software tools designed to build and deploy data platforms capable of efficiently managing and disseminating vast genomic datasets. Overture’s ability to fit in as a general solution for various collaborative efforts showcases its unique potential as a cornerstone in genomic research infrastructure. With Overture, we are working toward a future where opportunities for scientific discovery and innovation are no longer bottlenecked by challenges in the collection, storage, and sharing of genomics data.

## Methods

### Development methodology

The Overture team uses agile development practices to design, plan, and implement our software. Feature requests and bug reports are documented through GitHub issues and reviewed during monthly planning sessions. All tickets are documented, tracked, and prioritized through ZenHub. Developers peer-review “pull requests” and test them in our team’s quality assurance environments. All Overture source codebases are currently licensed under the GNU Affero General Public License v3.0.

## Availability of Supporting Source Code and Requirements Overture GitHub Repository

Project name: Overture

Project homepage: https://www.overture.bio/

GitHub link: https://github.com/overture-stack

Project documentation: https://docs.overture.bio/

Demo environment: https://demo.overture.bio/

Operating system(s): Platform independent

Programming language: Typescript, JavaScript, Java

License: AGPL-3.0

Bio.tools Unique Identifier: biotools:overture

RRID:SCR_026457

Other requirements:

- Docker Engine 19.03+ (or equivalent open-source alternatives)
- PostgreSQL database (for Songs)
- S3-compliant object storage (for Score)
- Elasticsearch 7.10+ or open-source equivalent (for Arranger)
- Node and or Maven required for development

Software Heritage PIDs:

- Arranger: swh:1:snp:d13c689513fb9e4dbf9978acc983b7251389ee52
- SONG: swh:1:snp:4527c299e4d0fa7167e30d901f58e25167b7b097
- Maestro: swh:1:snp:94dd1f9726979920c141c5f72e72424e4b71fd6d
- Stage: swh:1:snp:84d73e6e53be322f8443db17958bedba6a7c4693
- Score: swh:1:snp:ff210042483f6f25ab1408012b66c84816683863
- Ego: swh:1:snp:f34d155d92d46c1737da9293c6fb47af17898dfc
- Keycloak (third party tool): swh:1:snp:b6c1f520311456de88d94437a080495ffbad78f7

Overture microservices can be run as individual virtual containers, requiring Docker Engine version 19.03+ (or equivalent open-source alternatives). These microservices are compatible with Linux, Mac (Intel and Apple Silicon), and Windows platforms. Users can deploy and access all services locally (limited to HTTP) or externally by using custom domains that support HTTPS via TLS/SSL. All necessary configurations, including integration with other Overture microservices, are provided via environment variables. Due to variability in technologies and deployment contexts, we do not provide general guidance on maintaining production servers, including cost estimates. Documentation, including installation, configuration, and usage guides, can be found at https://www.overture.bio/getting-started/.

An Overture demo environment can be accessed from our website at https://demo.overture.bio/. We have also provided a QuickStart alongside platform guides for those interested in getting hands-on experience using our platform.

.

## Abbreviations

AI: artificial intelligence; APA: African Pathogen Data Sharing Archive; ARGO: ICGC Accelerating Research in Genomic Oncology; CLI: command line interface; DACO: Data Access Committee Office; EU GDPR: General Data Protection Regulation; EUCANCan: European-Canadian Cancer Network; FAIR: Findable, Accessible, Interoperable, Reusable; GA4GH: Global Alliance for Genomics and Health; GDC: Genomic Data Commons; HCMI: Human Cancer Models Initiative; ICGC-DCC: Data Coordination Center of the International Cancer Genome Consortium; IHCC: International Health Cohorts Consortium; JWT: JSON web token; ML: machine learning; OHCRN: Ontario Hereditary Cancer Research Network; OIDC: OpenID Connect; POPIA: Protection of Personal Information Act; RDPC: regional data-processing center; REWS: GA4GH Regulatory & Ethics Work Stream; SRA: Sequence Read Archive; TIGER: Translational Human Pancreatic Islet Genotype Tissue-Expression Resource Data Portal; UI: user interface.

## Supplementary Material

giaf038_GIGA-D-24-00541_Original_Submission

giaf038_GIGA-D-24-00541_R1

giaf038_Response_to_Reviewer_Comments_Original_Submission

giaf038_Reviewer_1_Report_Original_Submission Nelly Barret -- 12/23/2024

giaf038_Reviewer_2_Report_Original_Submission Osamu Ogasawara -- 12/26/2024

giaf038_Reviewer_3_Report_Original_Submission HervÃ© MÃ©nager -- 1/2/2025

## Data Availability

Overture microservices are open-source and freely available under the AGPL-3.0 license from the Overture GitHub organization, https://github.com/overture-stack/.
