# Supplementary material for: Overture: an open-source genomics data platform
Source: Gigascience. 2025 Apr 24;14:giaf038. doi: 10.1093/gigascience/giaf038 (PMC12020472; doi:10.1093/gigascience/giaf038)
Supplement: giaf038_GIGA-D-24-00541_Original_Submission [file giaf038_giga-d-24-00541_original_submission.pdf]

GigaScience

Overture: An Open-Source Genomics Data Platform

--Manuscript Draft--

Manuscript Number:	GIGA-D-24-00541
Full Title:	Overture: An Open-Source Genomics Data Platform
Article Type:	Technical Note
Funding Information:	Center for Biomedical Informatics and Information Technology, National Cancer Institute (#U24CA253529) — Not applicable
Abstract:	Abstract<br>Background<br>Next-generation sequencing has created many new technological challenges in organizing and distributing genomics datasets, which now can routinely reach petabyte scales. Coupled with data hungry artificial intelligence and machine learning applications, findable, accessible, interoperable and reusable genomics datasets have never been more valuable. While major archives like the Genomics Data Commons (GDC), Sequence Reads Archive (SRA), and European Genome-Phenome Archive EGA have improved researchers' ability to share and reuse data, the diversity of genomics research precludes any one-size-fits-all approach. In many cases, bespoke solutions are required and despite funding agencies and journals increasingly mandating reusable data practices, researchers still lack the technical support needed to meet the multifaceted challenges of data reuse.<br>Findings<br>Overture bridges this gap by providing open-source software for building and deploying customizable genomics data platforms. Its architecture consists of modular microservices, each of which is generalized with narrow responsibilities that together combine to create complete data management systems. These systems enable researchers to organize, share and explore their genomics data at any scale. Through Overture, researchers can connect their data to both humans and machines, fostering reproducibility and enabling new insights through controlled data sharing and reuse.<br>Conclusions<br>By making these tools freely available, we can accelerate the development of reliable genomic data management across the research community quickly, flexibly, and at multiple scales. Overture is an open-source project licensed under AGPLv3.0 with all source code publicly available from https://github.com/overture-stack and documentation on development, deployment and usage available from www.overture.bio.<br>Keywords: Research Software, Data Management, Genomics, open-source, open-science
Corresponding Author:	Mélanie Courtot<br>Ontario Institute for Cancer Research<br>Toronto, CANADA
Corresponding Author Secondary Information:	
Corresponding Author's Institution:	Ontario Institute for Cancer Research
Corresponding Author's Secondary Institution:	
First Author:	Mitchell Shiell
First Author Secondary Information:	
Order of Authors:	Mitchell Shiell
	Rosi Bajari
	Dusan Andric

Powered by Editorial Manager® and ProduXion Manager® from Aries Systems Corporation

	Jon Eubank
	Anders Richardsson
	Brandon Chan
	Azher Ali
	Bashar Allabadi
	Yelizar Alturmessov
	Jared Baker
	Ann Catton
	Kim Cullion
	Daniel DeMaria
	Patrick Dos Santos
	Henrich Feher
	Vincent Ferretti
	Francois Gerthoffert
	Minh Ha
	Robin Haw
	Atul Kachru
	Alexandru Lepsa
	Alexis Li
	Rakesh Mistry
	Hardeep Nahal-Bose
	Aleksandra Pejovic
	Sam Rich
	Leonardo Rivera
	Ciarán Schütte
	Lincoln Stein
	Edmund Su
	Robert Tisma
	Jaser Uddin
	Chang Wang
	Alex Wilmer
	Linda Xiang
	Junjun Zhang
	Mélanie Courtot
	Christina Yung
**Order of Authors Secondary Information:**	
**Additional Information:**	
**Question**	**Response**
Are you submitting this manuscript to a special series or article collection?	No

Powered by Editorial Manager® and ProduXion Manager® from Aries Systems Corporation

**Experimental design and statistics**<br><br>Full details of the experimental design and statistical methods used should be given in the Methods section, as detailed in our Minimum Standards Reporting Checklist. Information essential to interpreting the data presented should be made available in the figure legends.<br><br>Have you included all the information requested in your manuscript?	Yes
**Resources**<br><br>A description of all resources used, including antibodies, cell lines, animals and software tools, with enough information to allow them to be uniquely identified, should be included in the Methods section. Authors are strongly encouraged to cite Research Resource Identifiers (RRIDs) for antibodies, model organisms and tools, where possible.<br><br>Have you included the information requested as detailed in our Minimum Standards Reporting Checklist?	Yes
**Availability of data and materials**<br><br>All datasets and code on which the conclusions of the paper rely must be either included in your submission or deposited in publicly available repositories (where available and ethically appropriate), referencing such data using a unique identifier in the references and in the “Availability of Data and Materials” section of your manuscript.<br><br>Have you have met the above requirement as detailed in our Minimum Standards Reporting Checklist?	Yes

GigaScience has policies and guidelines in place for the use of generative AI-writing tools such as ChatGPT. If you have used such writing tools to assist with writing the manuscript this must be declared and cited in the text. Authors should not list AI-writing tools and other AI-assisted technologies as an author or co-author and should acknowledge that they are fully responsible for text generated or refined by AI-writing tools.<p><br><br>A summary of use (particularly in the introduction or among methods) needs to be included at the end of the paper, and the outputs should also be included as a supplementary file hosted in GigaDB or other open repositories. Please read our guidelines for more information. <p><br><br>By submitting to GigaScience, you are aware of the journal's AI-writing tools policy, and if you have declared use of such tools below, you have acknowledged this where appropriate in your manuscript and have made a summary of use and outputs available. <p><br><b>AI-assisted writing tools have been used in the preparation of this manuscript?	No

Overture: An Open-Source Genomics Data Platform

Authors: Mitchell Shiell[1,†], Rosi Bajari[1,†], Dusan Andric[1], Jon Eubank[1], Brandon F. Chan[1], Anders J. Richardsson[1], Azher Ali[1], Bashar Allabadi[1], Yelizar Alturmessov[1], Jared Baker[1], Ann Catton[1], Kim Cullion[1], Daniel DeMaria[1], Patrick Dos Santos[1], Henrich Feher[1], Francois Gerthoffert[1], Minh Ha[1], Robin A. Haw[1], Atul Kachru[1], Alexandru Lepsa[1], Alexis Li[1], Rakesh N. Mistry[1], Hardeep K Nahal-Bose[1], Aleksandra Pejovic[1], Samantha Rich[1], Leonardo Rivera[1], Ciarán Schütte[1], Edmund Su[1], Robert Tisma[1], Jaser Uddin[1], Chang Wang[1], Alex N. Wilmer[1], Linda Xiang[1], Junjun Zhang[1], Lincoln D. Stein[1,3], Vincent Ferretti[1,4], Mélanie Courtot[1,2,‡,#], Christina K. Yung[1,‡]

1. **Ontario Institute for Cancer Research (OICR)**
2. **University of Toronto Department of Medical Biophysics**
3. **University of Toronto Department of Molecular Genetics**
4. **Research Center of the CHU Sainte-Justine, University of Montreal**

† These authors contributed equally
‡ These authors contributed equally
# Corresponding Author

Abstract

Background

Next-generation sequencing has created many new technological challenges in organizing and distributing genomics datasets, which now can routinely reach petabyte scales. Coupled with data hungry artificial intelligence and machine learning applications, findable, accessible, interoperable and reusable genomics datasets have never been more valuable. While major archives like the Genomics Data Commons (GDC), Sequence Reads Archive (SRA), and European Genome-Phenome Archive EGA have improved researchers' ability to share and reuse data, the diversity of genomics research precludes any one-size-fits-all approach. In many cases, bespoke solutions are required and despite funding agencies and journals increasingly mandating reusable data practices, researchers still lack the technical support needed to meet the multifaceted challenges of data reuse.

Findings

Overture bridges this gap by providing open-source software for building and deploying customizable genomics data platforms. Its architecture consists of modular microservices, each of which is generalized with narrow responsibilities that together combine to create complete data management systems. These systems enable researchers to organize, share and explore their genomics data at any scale. Through Overture, researchers can connect their data to both humans and machines, fostering reproducibility and enabling new insights through controlled data sharing and reuse.

Conclusions

By making these tools freely available, we can accelerate the development of reliable genomic data management across the research community quickly, flexibly, and at multiple scales. Overture is an open-source project licensed under AGPLv3.0 with all source code publicly available from https://github.com/overture-stack and documentation on development, deployment and usage available from www.overture.bio.

Keywords: Research Software, Data Management, Genomics, open-source, open-science

First	Last	Preferred Email	Authorship
Mitchell	Shiell	mshiell@oicr.on.ca	Co-First Author
Rosi	Bajari	rosi.bajari@gmail.com	Co-First Author
Dusan	Andric	dusan.andric@gmail.com	Co-Author
Jon	Eubank	jeubank@oicr.on.ca	Co-Author
Anders	Richardsson	jrichardsson@oicr.on.ca	Co-Author
Brandon	Chan	bchan@oicr.on.ca	Co-Author
Azher	Ali	a2ali@oicr.on.ca	Co-Author
Bashar	Allabadi	basharlabadi@gmail.com	Co-Author
Yelizar	Alturmessov	yalturmessov@oicr.on.ca	Co-Author
Jared	Baker	jbaker@oicr.on.ca	Co-Author
Ann	Catton	acatton@oicr.on.ca	Co-Author
Kim	Cullion	kimcullion@gmail.com	Co-Author
Daniel	DeMaria	ddemaria@oicr.on.ca	Co-Author
Patrick	Dos Santos	pdossantos@oicr.on.ca	Co-Author
Henrich	Feher	hfeher@oicr.on.ca	Co-Author
Vincent	Ferretti	vincent.ferretti.hsj@ssss.gouv.qc.ca	Co-Author
Francois	Gerthoffert	fgerthoffert@gmail.com	Co-Author
Minh	Ha	hlminh2000@gmail.com	Co-Author
Robin	Haw	robin.haw@oicr.on.ca	Co-Author
Atul	Kachru	atul.kachru3845@gmail.com	Co-Author
Alexandru	Lepsa	lepsalex@gmail.com	Co-Author
Alexis	Li	alexis.li@oicr.on.ca	Co-Author
Rakesh	Mistry	rmistry@oicr.on.ca	Co-Author
Hardeep	Nahal-Bose	hnahal@oicr.on.ca	Co-Author
Aleksandra	Pejovic	pejovicaleks@gmail.com	Co-Author
Sam	Rich	srich@oicr.on.ca	Co-Author
Leonardo	Rivera	lrivera@oicr.on.ca	Co-Author
Ciarán	Schütte	cschutte@oicr.on.ca	Co-Author
Lincoln	Stein	lstein@oicr.on.ca	Co-Author
Edmund	Su	esu@oicr.on.ca	Co-Author
Robert	Tisma	rtisma@gmail.com	Co-Author
Jaser	Uddin	udjaser@outlook.com	Co-Author
Chang	Wang	garnwraly@gmail.com	Co-Author
Alex	Wilmer	djazium@gmail.com	Co-Author
Linda	Xiang	lxiang@oicr.on.ca	Co-Author
Junjun	Zhang	junjun.ca@gmail.com	Co-Author
Mélanie	Courtot	mcourtot@gmail.com	Co-Senior Author<br>Corresponding Author
Christina	Yung	christina.k.yung@gmail.com	Co-Senior Author

Background

Genomics research has benefited from a strong tradition of open science principles that have fostered comprehensive studies, transparent results, and accelerated scientific discovery[1]. As sequencing costs decrease, large and small research groups are increasingly generating massive multi-omics and single-cell data sets[2], often combined with clinical and imaging data[3]**.** This data abundance coincides with the emergence of machine learning (ML) and artificial intelligence (AI)[4], the biggest data-consuming activities in history. These emerging fields have sprouted from the wide availability of data; however, their solutions are limited by the quality of relevant data openly available for consumption[5]. Our brave new world now demands readily available software infrastructure to collect, organize, and share data.

In response, funding agencies and academic journals increasingly insist that projects generating large amounts of sequencing data respect FAIR (Findable, Accessible, Interoperable and Reusable) data practice[5]. This shift reflects a growing recognition and expectation of the researcher's role in facilitating data reuse. However, sharing genomics data is a multifaceted challenge:

- The volume of data often requires researchers to use cloud-based solutions that introduce new costs and expertise.[6]
- Sharing data across the research community must be done in an interoperable and sustainable fashion.[7]
- The legal, ethical, and social implications of genomics data sharing, including ownership, sovereignty, and data misuse, are extensive and evolving.[8-9].

While several resources for depositing genomic data, such as the Genomic Data Commons (GDC)[10], Sequence Read Archive (SRA)[10], and European Genome-Phenome Archive (EGA)[12], take on much of the responsibility of managing and archiving data, not every project is eligible to submit data to these repositories. The GDC focuses on human cancer genomes and is limited to DNA and RNA sequencing data in FASTQ and BAM formats[13]. The EGA only accepts human genomic and phenotypic data[14]. Alternatively, the SRA accepts a wide range of sequencing data types and is agnostic to the organism of origin[15]. However, like all archival resources, it faces fundamental challenges in accommodating niche and rapidly evolving data requirements[16]. This presents a serious challenge to research groups with large volumes of data that do not meet the requirements of existing archival resources, and projects with datasets that do not conform to the specific data model and file types accepted by these repositories get left out. Instead, these results must be shared ad hoc, such as in publication supplementary data files. To meet FAIR standards, genomic data must be maintained in an online system that allows for search and retrieval, is well-structured, and supports metadata and provenance tracking. Few research groups have the expertise to implement such a system[17], leaving groups with the choice of building the expertise in-house or hiring outside consultants, developers, and IT support staff. The first solution is inefficient, and the second one frequently exceeds available funding.

We created Overture[18-19] to enable researchers to build and deploy reproducible large-scale data platforms. With these platforms, researchers can maximize the potential of existing research, encouraging transparency, reproducibility, and reuse of data while maintaining oversight over its distribution. Other researchers can see their results, explore the data underpinning them, and reuse them to drive further discovery.

Results

Development of Overture

Overture was built based on a data portal and submission system developed to support the Data Coordination Center of the International Cancer Genome Consortium (ICGC-DCC)[20-21], a popular cancer genomics resource covering 84 worldwide projects, and molecular data from over 24,000 patients. The ICGC-DCC platform provided researchers with a user-friendly interface for efficient access, visualization, and analysis of its genomic data. After its launch, the portal's data exploration and analysis capabilities attracted attention from various research groups. Several of these groups successfully re-implemented and adapted the ICGC-DCC's infrastructure, including the Hartwig Medical Database[22] and the Translational Human Pancreatic Islet Genotype Tissue-Expression Resource Data Portal (TIGER)[23].

However, the reusability of the ICGC-DCC infrastructure was met with significant technical challenges, particularly tied to its monolithic architecture and numerous hard-coded elements, which made it unnecessarily difficult to replicate and implement the system in projects with similar needs and limited the ability to scale in production. These limitations prompted a strategic shift toward a more flexible and scalable microservice architecture. This was chosen for several key advantages; *(1) Scalability:* microservices enable individual system components to scale independently. *(2) Flexibility:* each microservice can be deployed and upgraded separately, easing the development of new features and modifications to existing ones. *(3) Resilience:* if one microservice encounters a failure, other instances reduce or even avert downtime by load balancing accordingly. This resulted in the development of Overture, a collection of reusable and modular microservices that serve as a general solution for building and deploying data platforms.

Platform Overview

Overture platforms are highly flexible yet fundamentally have a standard feature set provided by its core software components (Figure & Table 1). The target users for our data platform's core functionalities can be divided into three categories:

1. Data consumers retrieving data from the platform.
2. Data providers submitting data to the platform.
3. Data administrators who configure and maintain the platform.

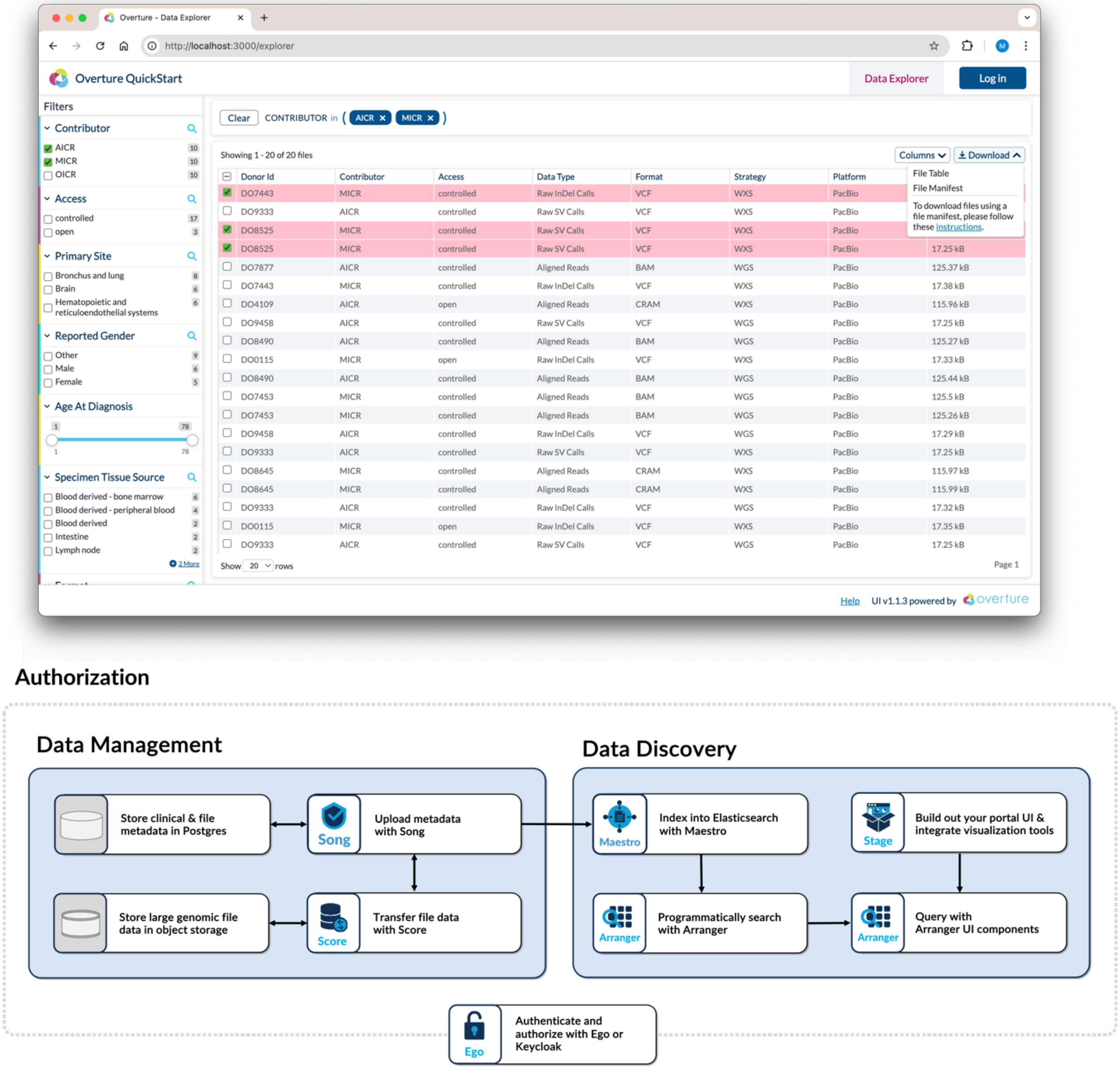

Figure 1. Platform Overview: On the front-end, Stage provides the basic user interface (UI), including navigation menus; as well as data exploration, login and profile pages. Arranger's library of search UI components then integrates with Stage to offer a configurable search facet panel, data table, and filter summary panel. Login and profile pages integrate with Keycloak[24] or Ego, which provides authentication and authorization for users and applications. Behind the scenes, Song and Score facilitate data management, retrieval and submission. Score transfers large genomic files to and from S3-compatible object storage, while Song stores and handles the files' metadata. These databases are indexed by Maestro into unified Elasticsearch[25] file-centric and analysis-centric indices. Arranger then uses these to produce a GraphQL[26] search API that connects with its front-end library components on the data exploration page. Combined together, these services broadly enable the secure and scalable reuse of genomics data.

.

Table 1. Overview of Overture Stack Software Components: Overture comprises six core components that work in concert to create genomics data management systems.

Product Name	Code repository	Brief description
Song	https://github.com/overture-stack/song	Metadata management with an automated submission validation system.
Score	https://github.com/overture-stack/score	File Transfer Microservice that supports fault-tolerant multi-part parallel transfer
Maestro	https://github.com/overture-stack/maestro	Indexes metadata from Song into Elasticsearch search indices, to be consumed by Arranger.
Arranger	https://github.com/overture-stack/arranger	Data search and exploration API, and accompanying library of UI components that can be easily integrated in a data portal.
Ego	https://github.com/overture-stack/ego	OAuth 2.0 authorization service that supports multiple OpenID Connect (OIDC) identity providers.
Stage	https://github.com/overture-stack/Stage	A React-based user interface designed to allow easy deployment of browser-friendly data portals.

Data Retrieval

Data retrieval starts from the Stage data exploration page (figure 2), where users can filter data using the Arranger search facets. These enable rapid and efficient data filtering using checkboxes, date ranges, sliders, and quick search input boxes, allowing users to narrow their queries and focus on relevant data subsets. Filtered datasets are presented in the Arranger data table, which provides sortable columns, file counts, and pagination. All query parameters are summarized within an Arranger filter panel at the top of the page, giving users a clear overview of their search criteria. Users can easily share these queries using the browser URL, which gets updated with the filter parameters in real-time.

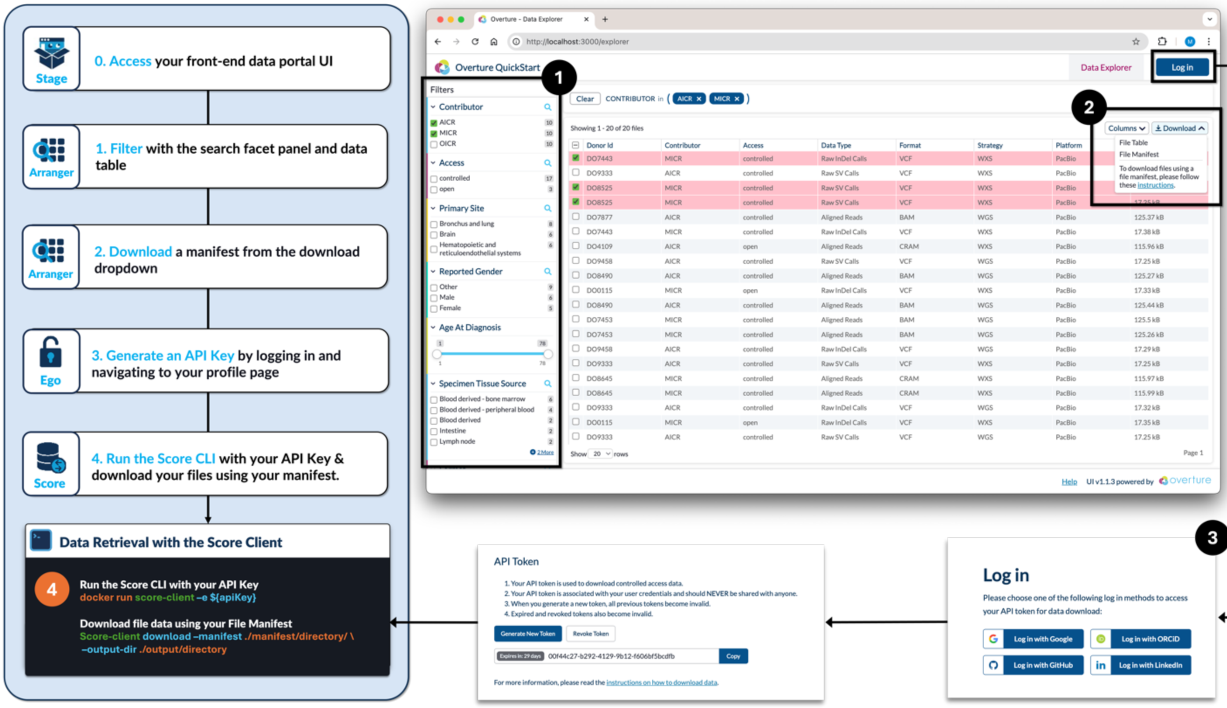

Figure 2. Data Retrieval Workflow: Users first filter data via Arranger's search components in the Stage UI's data explorer. Once they have selected a subset, they then download a 'file manifest' from the download dropdown. To access Song and Score data, users log in through Stage's auth integration and obtain their API key from the profile page. This API key is provided when installing the score client. Finally, files are downloaded to the user's device using the Score client's download command, specifying the file manifest and desired output directory.

Once the users have identified relevant data, they can select the download dropdown, which provides options for downloading metadata or a file manifest in a TSV format. The manifest file allows users to download their files of interest directly from the resources database and object storage using Overture's command line interface (CLI) tools, specifically the Song and Score clients. These CLI tools are needed as massive genomic datasets require reliable multi-part parallel download sessions unsuitable for a browser. To ensure secure access to data, users must supply a valid API key when installing the Song and Score clients. For controlled-access data, researchers will be able to retrieve their API key after their data access request is granted by typically the relevant data access committee. Log in to the web portal is facilitated by either Keycloak or Ego, which supports popular identity providers, including Google, ORCiD, and GitHub.

Data Submission

Overture's submission process has been designed to ensure data integrity by facilitating data tracking and data model compliance. In Overture, a set of one or more files plus the metadata describing that collection of files is called an analysis. To upload an analysis, data submitters first organize their metadata files, typically using a spreadsheet editor alongside a data dictionary supplied by the resource administrator. The data dictionary describes the required metadata fields and the expectations for the syntax of each field. Once converted to JSON, the Song Client upload command can be used to send the metadata submission to the resources Song server for validation against the admin defined data model. If there are any issues with the metadata, the user will be provided a detailed error message. If successful,

the user will be provided a success message and an auto-generated analysis ID for future reference within the system.

After submitting metadata and establishing an analysis ID, the submitter uploads file data using Song and Score clients. First, a file manifest is generated using the Song client manifest command, along with the directory where the files are located and the analysis ID assigned to the relevant metadata. This links the uploaded files to the metadata in Song's database. The manifest and Score Client upload command is then used to transfer files to object storage. Once uploaded, Song can dynamically update file metadata, such as md5 checksums, to the appropriate analysis file within its database.

All data uploaded to the resource are, by default, in an unpublished state. Publication controls allow administrators and data providers to coordinate and prepare data releases in a predictable and timely manner. All publication controls are facilitated by Song. When data is ready for search and download, administrators can make it available by updating the desired analyses to a published state. If data is no longer relevant, the data administrators can make them unavailable to downstream services by setting analyses to a suppressed state.

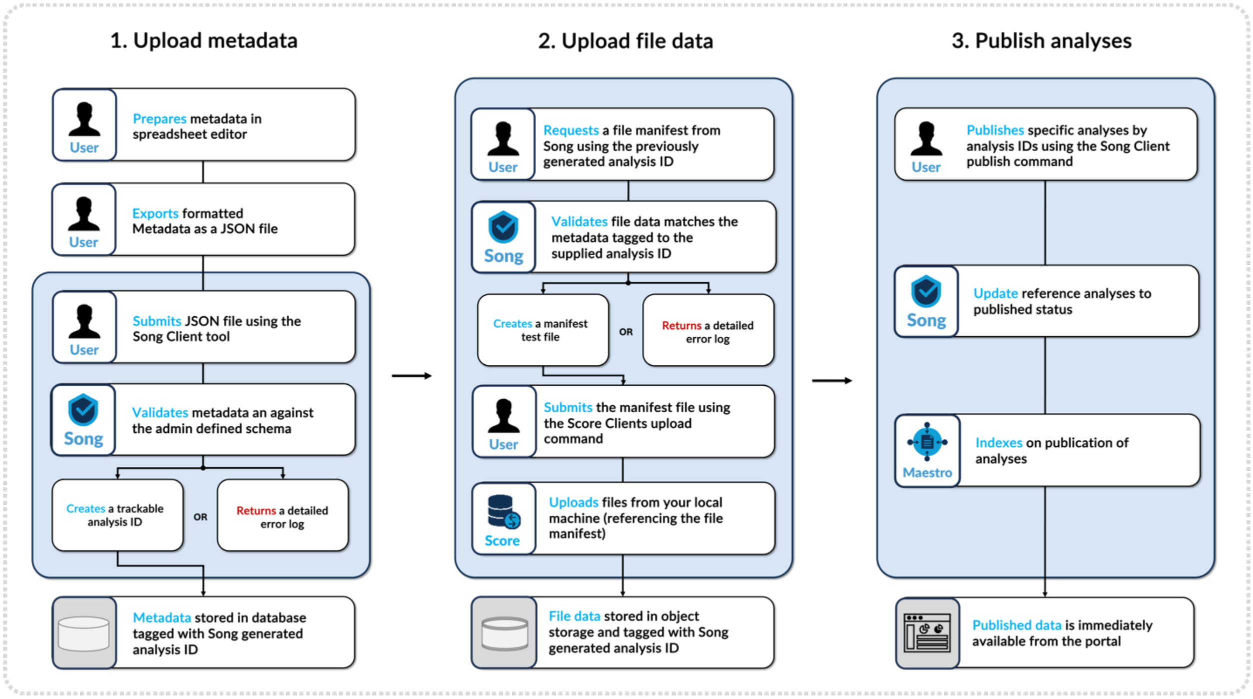

Figure 3. Data Submission Workflow: Overture's submission process enhances data integrity with data tracking and data model adherence. It involves organizing metadata files, converting them to JSON, and uploading via the Song Client for validation. Successful submissions receive an auto-generated analysis ID. File data is then uploaded using Song and Score clients, generating a file manifest linked to the metadata. All data starts unpublished and is managed through Song's publication controls for coordinated data releases.

Data Administration

Data administrators are responsible for maintaining and configuring the data resource including providing the data model, configuring the portal search interface, and managing users' access and permissions. With Overture, administrators have the flexibility to define the data model of their resources

according to their desired standards. This involves outlining the structure and syntax of the data model in JSON format. Once the JSON schema is created, the administrator can submit it to Overture's Song microservice through the Song API. This schema then serves to verify all future submissions to the resource. Multiple schemas can be registered to the platform, enabling validation for various data types.

Given the potential diversity of projects that can use Overture, administrators require flexibility in customizing their portal front-end interface. With Arranger's library of UI components, among other customizations, administrators can configure the display settings of the search facets and data table on the portal exploration page. This includes configurations for what fields are visible, how they are displayed, and how users can interact with them.

Admins also have the flexibility to customize the portal's content by building on top of the Stage UI. Stage is a React-based single-page web application designed to provide a foundation for building any data portal. Its default features include a header, footer and navigation menus. With functional knowledge of React, you can easily theme and extend Stage to include custom pages and menu options, such as funding acknowledgments, documentation, and data release statements.

Depending on the authorization service chosen, admins can manage user permissions through the Ego admin UI or KeyCloak. These allow admins to apply role-based permissions granting read or write access to users and applications through JSON Web Tokens (JWTs) or API Keys.

Impact

The types of groups that can deploy Overture platforms can be categorized into three segments based on the scale of data and their level of in-house expertise:

1. **Large-scale Consortiums** with tens to hundreds of thousands of samples, and technical staff to support the expansion of both hardware and software infrastructure. They may also need to distribute data across multiple locations and jurisdictions.
2. **Medium to small-sized labs, programs and institutions** with hundreds to thousands of samples and limited system administration support.

We will address each of these groups in the following sections.

Impact on Consortium-level Projects

Multiple consortium-level projects have successfully reused Overture components, reducing development efforts and, in turn, driving further enhancement. As a testament to the benefits of a modular approach, Overture has significantly impacted several consortium-level projects (Table 1).

Case Study ICGC-ARGO

ICGC Accelerating Research in Genomic Oncology (ARGO)[27] is a global initiative to provide precision oncology knowledge. Intending to analyze genomes from 100,000 cancer patients, ICGC-ARGO aims to collect genomic data alongside high-quality clinical data and make it available to the research community quickly and responsibly.

ICGC-ARGO operates at a global scale. To satisfy the legal requirements of data sovereignty, ARGO needs to implement a distributed network of servers or nodes located within each country of data origin. Thanks to Overture, the ARGO development team is deploying a global network of interoperable regional data processing centers (RDPCs) where data is submitted and stored within each country of origin. Each RDPC leverages the core Overture components - Song, Score and Maestro. The ARGO platform then federates queries through an Arranger server, enabling search across the global network of RDPC nodes from the ARGO Data Platform (https://platform.icgc-argo.org/) .

Table 2. Impact on consortium-level projects: an overview of consortium-level data-sharing initiatives. Each initiative briefly describes the project and the Overture components that help drive it.

Project	Description	Component(s) used	Data
**Kids First Data Resource Portal**[28]	The Kids First Data Portal provides access to genomic and clinical data sets generated by the Kids First Pediatric Cancer, the Rare Diseases Data Resource and other NCI-supported pediatric genomics projects. The data sets are stored in a secure, centralized repository and made available worldwide to researchers and the public.	Arranger	34,000 Human Genomes
**Human Cancer Models Initiative (HCMI) Searchable Catalog**[29]	The HCMI catalogs cancer models alongside clinical, biospecimen, and molecular data. This data-sharing platform also includes protocols, informed consent templates, and clinical data forms, making it an all-in-one resource for translational cancer researchers.	Arranger	307 Cancer Models
**International Health Cohorts Consortium (IHCC) Cohort Atlas**[30]	The IHCC is improving clinical care and population health by aggregating large genomic data cohorts to help translational researchers uncover the biological and genetic factors of disease. The *IHCC Cohort Atlas* is the global data-sharing platform hosting genomics data from large cohorts (100k+). **Overture Arranger provides shared discovery tools and interoperability with other international data portals.**	Arranger	Human cohort metadata for 34 million participants
**International Cancer Genome Consortium - Accelerating Research in Genomic Oncology (ICGC ARGO)**[27]	ICGC-ARGO is a global initiative to provide precision oncology knowledge to the world. With the goal of analyzing genomes from 100,000 cancer patients, ICGC-ARGO aims to collect genomic data alongside high-quality clinical data and make it available to the research community in a rapid and responsible way. **(Re)use and extension of Overture components supports controlled data storage and access.**	Ego, Song, Score, Maestro, Arranger	37,222 Genomic files
**VirusSeq Data Portal**[31]	The VirusSeq Data Portal is an open-source and open-access data portal for all Canadian SARS-CoV-2 sequences as well as associated non-personal contextual data. It harmonizes, validates and automates submission to international databases. **Using Overture, the portal was created within a 4 week timeframe.** Initially intended to store 150,000 sequences it has scaled to host over 500,000 genomes.	Ego, Song, Score, Maestro, Arranger	98,266 Genomic file
**European-Canadian Cancer Network (EUCANCan)**[32]	The EUCANCan project offers a novel solution to managing and sharing cancer genomic data. **Overture's Maestro enables federated search across 3 EUCANCan nodes.** Instead of consolidating data in one	Overture Data Management System (DMS)	N/A

	control center following some established process and timeline, each data node manages their own data locally. The nodes agree on the set of metadata that can be queried in a unified data portal that will then point to the location of the genomic data.		
**Ontario Hereditary Cancer Research Network (OHCRN)**[33]	OHCRN aims to harmonize Information from individuals with Hereditary Cancer Syndrome in order to better understand and advance the prevention, early detection and treatment of these cancers.	Arranger	N/A
**African Pathogen Data Sharing Archive (APA)**[34]	This data-sharing platform is being developed to enable real time pathogen genomics sharing and exchange across Africa[35]. The portal allows users to upload, share, explore, and download pathogen sequences and associated metadata as per data use guidelines provided by each country. **Reusing Overture has enabled LMICs institutions to build local capacity and deploy their own platform.**	Ego, Song, Score, Maestro, Arranger	N/A

Medium to small laboratories and institutions

Overture has demonstrated its efficacy for large-scale genomics data platforms, with successful deployments across many projects. However, it is important to acknowledge that the projects presented so far only include consortium-level projects. While microservice architectures offer numerous advantages, the technical complexity of deploying our platforms have been a significant barrier for small and medium sized groups. To lower adoption barriers, we've identified and addressed three fundamental questions: How can potential new users see our platform in action? How can they openly experiment with the platform? And how can they take ownership of it?

To address the first question, we developed an Overture demo portal (https://demo.overture.bio/). This environment is accessible directly from our homepage and offers new and prospective users an immediate, interactive introduction to Overture's capabilities. The demo features a representative mock dataset on the exploration page and includes supplementary content within the portal's Stage UI, providing a surface level overview of the platform's functionality.

To facilitate open experimentation of our platform, we introduced the Overture Quickstart, a Docker Compose[36] that enables users to deploy the entire platform locally within minutes, complete with pre-populated mock data and a pre-configured admin user. To accompany our localized Quickstart setup we expanded our documentation to include platform guides that cover essential processes such as data submission, download, and core administrative tasks required for configuring an operational Overture platform.

To address ownership, we containerized and standardized the installation process for our microservices. Each service can now be installed using Docker and an environment variable file, ensuring broad portability across diverse computing environments. Furthermore, we now provide a comprehensive end-to-end deployment guide, which meticulously details each stage, service, and environment variable required for establishing a base Overture platform. Moving forward we hope to provide guides and resources for automated deployments of a variety of ideal and reproducible environments leveraging popular tooling's like Terraform[37] and Helm[38]. Through these initiatives, we hope to significantly reduce

barriers to adoption, making Overture more accessible to a broader range of research groups, regardless of their scale or technical expertise.

Discussion

As the software engineering team at OICR, we build data platforms with a diverse range of requirements. When our solutions prove widely applicable, they are refined into more generic tools and distributed as part of the Overture Suite. In the following sections, we will discuss some of our current challenges and how they are guiding the expansion of the Overture suite.

Overture's development has been limited until recently for use on cancer genomics data. However, we are finding an increasing demand for projects that require data outside the context of cancer genomics[31,35]. In response, we are developing an updated data-agnostic tabular submission system to complement our existing infrastructure. This update allows us to cater to a broader range of use cases, such as pathogenic data, without additional development.

Recent data protection laws including the General Data Protection Regulation (EU GDPR)[40] and the Protection of Personal Information Act (POPIA) in South Africa[41] have created a shift in how we manage data across borders. Where data could formerly be transferred across jurisdictions, the current data protection laws prohibit this. Instead, we must host data in its geolocation of origin, deploying instances of the original platform in each country. This approach - *federation* - requires new means to discover the data at each node of the network instead of relying on centralized indexes. In response, we are improving our search API service Arranger, to aggregate search results across different nodes of arranger instances enabling users to query datasets from other countries of origin while still maintaining the privacy of the individual. The extent to which the data can be aggregated and further explored centrally will need to be reviewed by ethics experts; our initial foray in the area has led to different interpretations and variable willingness to share data. Clear guidance from policy and legal experts will be required to achieve our vision of building a truly federated platform; we are tackling this through collaboration with the ICGC-ARGO ethical working group[42], as well as the GA4GH Regulatory & Ethics Work Stream (REWS)[43].

The management of patient consent and controlled access to data has become a standard requirement for large-scale human genomics platforms. In the past, to gain access to data, researchers were required to submit paper and PDF forms. This process can take months and has contributed to significant barriers to data access[7]. In response to challenges in controlled data access, we created an online application module for the ICGC-ARGO project. The Data Access Committee Office (DACO) application enables researchers to log in to an online portal, fill in, sign their application, and send it for review electronically to the Data Access Committee. The Data Access officer reviews applications through an online dashboard through which they can request more information or approve/deny the applications. This process has reduced the average approval time from four weeks to 3.5 days for over 400 applications across 35 countries. For patient consent, we are building a virtual patient enrollment portal for the Ontario Hereditary Cancer Research Network. Designed to address real world project demands, the patient consent portal enables study participants to provide consent and agree to online data sharing in both an ethical and accessible manner. Patient consent and controlled access to data are core requirements for platforms handling sensitive human data. Therefore, we are working to incorporate these two applications - the DACO system and the virtual patient enrollment portal - as new Overture components.

Conclusions

The rapid expansion of genomics research and the intricate challenges of organizing and distributing its data present formidable obstacles for the field of genomics. Overture addresses this by offering software tools designed to build and deploy data platforms capable of efficiently managing and disseminating vast genomic datasets. Overture's ability to fit in as a general solution for various collaborative efforts showcases its unique potential as a cornerstone in genomic research infrastructure. With Overture, we are working towards a future where opportunities for scientific discovery and innovation are no longer bottlenecked by challenges in the collection, storage and sharing of genomics data.

Methods

Development Methodology

The Overture team uses agile development practices to design, plan, and implement our software. Feature requests and bug reports are documented through GitHub issues and reviewed during monthly planning sessions. All tickets are documented, tracked, and prioritized through ZenHub. Developers peer-review "pull requests" and test them in our team's QA environments. All Overture source codebases are currently licensed under the GNU Affero General Public License v3.0.

Availability of supporting source code and requirements

- **Project name:** Overture
- **Project home page:** https://www.overture.bio/
- **Operating system(s):** Linux, Mac (Intel and Apple Silicon), Windows
- **Programming language:** Typescript, JavaScript, Java
- **Other requirements:**
    - Docker Engine 19.03+ (or equivalent open-source alternatives)
    - PostgreSQL database (for Songs)
    - S3-compliant object storage (for Score)
    - Elasticsearch 7.10+ or open-source equivalent (for Arranger)
    - Node and or Maven required for development
- **License:** AGPLv3.0

Overture microservices can be run as individual virtual containers, requiring Docker Engine version 19.03+ (or equivalent open-source alternatives). These microservices are compatible with Linux, Mac (Intel and Apple Silicon), as well as Windows platforms. Users can deploy and access all services locally (limited to HTTP) or externally by using custom domains that support HTTPS via TLS/SSL. All necessary configurations, including integration with other Overture microservices, are provided via environment variables. Documentation, including installation, configuration and usage guides, can be found at https://www.overture.bio/getting-started/.

An Overture demo environment can be accessed from our website at https://demo.overture.bio/. We have also provided a QuickStart alongside platform guides for those interested in getting hands-on experience using our platform.

List of abbreviations

African Pathogen Data Sharing Archive (APA)
Artificial Intelligence (AI)
Command Line Interface (CLI)
Data Access Committee Office (DACO)
Data Coordination Center of the International Cancer Genome Consortium (ICGC-DCC) European-Canadian Cancer Network (EUCANCan)
Findable, Accessible, Interoperable, Reusable (FAIR)
Global Alliance for Genomics and Health (GA4GH)
GA4GH Regulatory & Ethics Work Stream (REWS)
General Data Protection Regulation (EU GDPR)
Genomic Data Commons (GDC)
Human Cancer Models Initiative (HCMI)
ICGC Accelerating Research in Genomic Oncology (ARGO)
International Health Cohorts Consortium (IHCC)
JSON Web Tokens (JWTs)
Machine Learning (ML)
Ontario Hereditary Cancer Research Network (OHCRN)
OpenID Connect (OIDC)
Protection of Personal Information Act (POPIA)
Regional Data Processing Centers (RDPCs)
Sequence Read Archive (SRA)
Translational Human Pancreatic Islet Genotype Tissue-Expression Resource Data Portal (TIGER)
User Interface (UI)

Declarations

Ethics approval and content to participate

Not applicable

Consent for publication

Not applicable

Competing interests

The authors declare that they have no competing interests.

Funding

Overture is supported by grant #U24CA253529 from the National Cancer Institute at the US National Institutes of Health, and additional funding from Genome Canada, the Canada Foundation for Innovation, the Canadian Institutes of Health Research, CANARIE, and the Ontario Institute for Cancer Research.

Authors' contributions:

All authors read and approved the final manuscript.

M.C., C.Y. contributed to all stages of the project's lifecycle, from inception to completion, formulating overarching goals and aims, acquiring funding and reviewing and editing the manuscript. L.S., V.F. contributed to multiple stages of the project's life cycle, particularly in its early stages by formulating overarching goals and aims and acquiring funding for development. H.N-B., E.S., L.X., A.A., B.A., Y.A., J.B., A.C., K.C., D.D., P.D.S., H.F., A.Li., R.M., S.R., L.R., C.S., J.U., A.W., J.Z. developed and implemented core features for the Overture project, contributing to its open-source codebase and enhancing system functionality. J.E., A.R., D.A. and A.Lepsa., designed the high-level system architecture and technical framework while contributing to the open-source codebase and enhancing overall functionality.F.G. and R.H. contributed to the project conceptualization, helping shape its core vision and objectives while also managing and coordinating project activities, ensuring alignment with goals and timelines. B.C., A.K. and A.P. and A.Li. worked on Overture as key administrators, overseeing software development lifecycle from planning to execution. The manuscript was written by M.S. and R.B. with reviews and edits primarily contributed by J.E., A.R., B.C., R.H., M.C., C.Y., L.S., V.F.

Acknowledgements

Not applicable

References

1. Gates, A. J., Gysi, D. M., Kellis, M. & Barabási, A.-L. A wealth of discovery built on the Human Genome Project — by the numbers. *Nature* **590**, 212–215 (2021).
2. Stephens, Z. D. *et al.* Big Data: Astronomical or Genomical? *PLoS Biol* **13**, e1002195 (2015).
3. Subramanian, I., Verma, S., Kumar, S., Jere, A. & Anamika, K. Multi-omics Data Integration, Interpretation, and Its Application. *Bioinform Biol Insights* **14**, 117793221989905 (2020).
4. Sharma, A., Lysenko, A., Jia, S., Boroevich, K. A. & Tsunoda, T. Advances in AI and machine learning for predictive medicine. *J Hum Genet* (2024) doi:10.1038/s10038-024-01231-y.
5. Wilkinson, M. D. et al. The FAIR Guiding Principles for scientific data management and stewardship. Sci Data 3, 160018 (2016).
6. Tanjo, T., Kawai, Y., Tokunaga, K., Ogasawara, O. & Nagasaki, M. Practical guide for managing large-scale human genome data in research. J Hum Genet 66, 39–52 (2021).
7. Powell, K. The broken promise that undermines human genome research. Nature 590, 198–201 (2021).
8. Rehm, H. L. et al. GA4GH: International policies and standards for data sharing across genomic research and healthcare. Cell Genomics 1, 100029 (2021).
9. Byrd, J. B., Greene, A. C., Prasad, D. V., Jiang, X. & Greene, C. S. Responsible, practical genomic data sharing that accelerates research. Nat Rev Genet 21, 615–629 (2020).
10. National Cancer Institute. Genomic Data Commons. https://gdc.cancer.gov/ (accessed July 24, 2024).
11. National Center for Biotechnology Information. Sequence Read Archive. https://www.ncbi.nlm.nih.gov/sra (accessed July 24, 2024).
12. European Genome-phenome Archive. https://ega-archive.org/ (accessed July 24, 2024).
13. National Cancer Institute. Genomic Data Commons Data Submission Process. https://gdc.cancer.gov/node/216/ (accessed July 24, 2024).
14. European Bioinformatics Institute. EGA Quick Tour: Submitting data to EGA. https://www.ebi.ac.uk/training/online/courses/ega-quick-tour/submitting-data-to-ega/ (accessed July 24, 2024).
15. National Center for Biotechnology Information. SRA Submission Format. https://www.ncbi.nlm.nih.gov/sra/docs/submitformats/ (accessed July 24, 2024).

16. Leigh, D. M. et al. Best practices for genetic and genomic data archiving. Nat Ecol Evol 8, 1224–1232 (2024).
17. Perrier, L., Blondal, E. & MacDonald, H. The views, perspectives, and experiences of academic researchers with data sharing and reuse: A meta-synthesis. PLoS ONE 15, e0229182 (2020).
18. Overture [Internet]. Software for big data genomic science. https://www.overture.bio/ (accessed 24 July 2024).
19. Overture stack [Internet]. Source code. https://github.com/overture-stack (accessed 25 July 2024).
20. ICGC Data Portal [Internet]. Available from: http://dcc.icgc.org (accessed 19 April 2024).
21. Zhang, J. et al. The International Cancer Genome Consortium Data Portal. Nat Biotechnol 37, 367–369 (2019).
22. Hartwig Medical Foundation. Database of metastatic cancer. https://www.hartwigmedicalfoundation.nl/en/data/database/ (accessed 25 July 2024).
23. Alonso, L. et al. TIGER: The gene expression regulatory variation landscape of human pancreatic islets. Cell Reports 37, 109807 (2021).
24. Keycloak [Internet]. Open Source Identity and Access Management available from: https://www.keycloak.org/ (accessed 25 July 2024).
25. Elasticsearch [Internet]. Available from: https://www.elastic.co/ (accessed 25 July 2024).

26. GraphQL [Internet]. A query language for your API. Available from: https://graphql.org/ (accessed 25 July 2024).
27. International Cancer Genome Consortium Accelerating Research in Genomic Oncology. ICGC ARGO Data Platform. https://platform.icgc-argo.org/ (accessed 25 July 2024).
28. Kids First Data Resource Center. Kids First Data Resource Portal. https://portal.kidsfirstdrc.org/ (accessed 25 July 2024).
29. National Cancer Institute. Human Cancer Models Initiative (HCMI) Searchable Catalog. https://hcmi-searchable-catalog.nci.nih.gov/ (accessed 25 July 2024).
30. International Health Cohorts Consortium. IHCC Cohort Atlas. https://ihccglobal.org/cohort-atlas/ (accessed 25 July 2024).
31. VirusSeq Data Portal. https://virusseq-dataportal.ca/ (accessed 25 July 2024).
32. European-Canadian Cancer Network. EUCANCan Data Portal. https://eucancan.com/ (accessed 25 July 2024).
33. Ontario Hereditary Cancer Research Network. OHCRN Data Portal. https://ohcrn.ca/ (accessed 25 July 2024).
34. African Pathogen Data Sharing and Archive Platform. APA Portal. https://apaportal.sanbi.ac.za/ (accessed 25 July 2024).
35. Christoffels, A. et al. A pan-African pathogen genomics data sharing platform to support disease outbreaks. Nat Med 29, 1052–1055 (2023).
36. Docker Inc. Docker Compose. https://docs.docker.com/compose/ (accessed 25 July 2024).
37. HashiCorp. Terraform. https://www.terraform.io/ (accessed 25 July 2024).
38. The Linux Foundation. Helm. https://helm.sh/ (accessed 25 July 2024).
39. Gill, E. E. *et al.* The Canadian VirusSeq Data Portal & Duotang: open resources for SARS-CoV-2 viral sequences and genomic epidemiology. Preprint at https://doi.org/10.48550/ARXIV.2405.04734 (2024).
40. *The EU General Data Protection Regulation (GDPR): A Commentary*. (Oxford University PressNew York, 2020). doi:10.1093/oso/9780198826491.001.0001.
41. POPIA. Protection of Personal Information Act. [online] Available at: https://popia.co.za/ [Accessed 8 Aug. 2024].
42. ICGC-ARGO. ARGO Project. [online] Available at: https://www.icgc-argo.org/page/104/aegc [Accessed 8 Aug. 2024].

43. Global Alliance for Genomics and Health (GA4GH). Regulatory & Ethics Work Stream. [online] Available at: https://www.ga4gh.org/work_stream/regulatory-ethics/ [Accessed 8 Aug. 2024].

Personal Cover

Click here to access/download;Personal Cover;GigaScience Cover Letter.docx

OICR
Ontario Institute for Cancer Research

MaRS Centre
661 University Avenue, Suite 510
Toronto, ON M5G 0A3 Canada

Telephone 416-977-7599
Toll-free 1-866-678-6427
oicr.on.ca

8 November 2024

Laurie Goodman, PhD
Editor-in-Chief
GigaScience

Dear Laurie,

How are you? It's been ages since our time together at CSHL. I've been up in Toronto for the past 17 years and having a great time. I hope you are similarly situated.

At long last I have a manuscript for your consideration for publication in GigaScience. This is a paper written by my colleagues, Melanie Courtot and Christina Yung, describing the Overture genomic data management system, a modular and highly scalable software system that provides all the tools needed to manage a large genomic data integration project. It provides support for genomic data and metadata submission and validation, data transfer, indexing, and search. Most notably, it makes it easy to mount a public portal for data discovery, browsing, download and integrated analytics.

Overture is being used by several big genomic data projects, including the International Cancer Genome Consortium, the NIH Human Cancer Models Initiative, the Gabriella Miller Kids First data portal, and Canada's VirusSeq repository of COVID-19 genomes.

As far as I'm aware, there is no other open source package that provides Overture's capabilities. It also has a variety of innovative software engineering design features under the hood. Given GigaScience's emphasis on Big Data research, I think the audience is a good match, and I hope you agree!

Very best wishes,

Lincoln D Stein (Lincoln)

Lincoln D. Stein, MD/PhD

Collaborate. Translate. Change lives.

Scientific Director (interim), OICR
Head, Adaptive Oncology Program, OICR
Senior Principal Investigator, OICR
Professor, Molecular Genetics, University of Toronto

Email: lincoln.stein@gmail.com
Phone: (416) 817-8240
Executive Assistant: michelle.xin@oicr.on.ca

Collaborate. Translate. Change lives.
