# Supplementary material for: Overture: an open-source genomics data platform
Source: Gigascience. 2025 Apr 24;14:giaf038. doi: 10.1093/gigascience/giaf038 (PMC12020472; doi:10.1093/gigascience/giaf038)
Supplement: giaf038_GIGA-D-24-00541_R1 [file giaf038_giga-d-24-00541_r1.pdf]

GigaScience
Overture: An Open-Source Genomics Data Platform
--Manuscript Draft--

**Manuscript Number:**	GIGA-D-24-00541R1
**Full Title:**	Overture: An Open-Source Genomics Data Platform
**Article Type:**	Technical Note
**Funding Information:**	Center for Biomedical Informatics and Information Technology, National Cancer Institute (#U24CA253529) — Not applicable
**Abstract:**	Abstract<br>Background<br>Next-generation sequencing has created many new technological challenges in organizing and distributing genomics datasets, which now can routinely reach petabyte scales. Coupled with data hungry artificial intelligence and machine learning applications, findable, accessible, interoperable and reusable genomics datasets have never been more valuable. While major archives like the Genomics Data Commons (GDC), Sequence Reads Archive (SRA), and European Genome-Phenome Archive (EGA) have improved researchers' ability to share and reuse data, and general-purpose repositories such as Zenodo and Figshare provide valuable platforms for research data publication, the diversity of genomics research precludes any one-size-fits-all approach. In many cases, bespoke solutions are required and despite funding agencies and journals increasingly mandating reusable data practices, researchers still lack the technical support needed to meet the multifaceted challenges of data reuse.<br>Findings<br>Overture bridges this gap by providing open-source software for building and deploying customizable genomics data platforms. Its architecture consists of modular microservices, each of which is generalized with narrow responsibilities that together combine to create complete data management systems. These systems enable researchers to organize, share and explore their genomics data at any scale. Through Overture, researchers can connect their data to both humans and machines, fostering reproducibility and enabling new insights through controlled data sharing and reuse.<br>Conclusions<br>By making these tools freely available, we can accelerate the development of reliable genomic data management across the research community quickly, flexibly, and at multiple scales. Overture is an open-source project licensed under AGPLv3.0 with all source code publicly available from https://github.com/overture-stack and documentation on development, deployment and usage available from www.overture.bio.<br>Keywords: Research Software, Data Management, Genomics, open-source, open-science
**Corresponding Author:**	Mélanie Courtot<br>Ontario Institute for Cancer Research<br>Toronto, CANADA
**Corresponding Author Secondary Information:**	
**Corresponding Author's Institution:**	Ontario Institute for Cancer Research
**Corresponding Author's Secondary Institution:**	
**First Author:**	Mitchell Shiell
**First Author Secondary Information:**	
**Order of Authors:**	Mitchell Shiell
	Rosi Bajari
	Dusan Andric

Powered by Editorial Manager® and ProduXion Manager® from Aries Systems Corporation

	Jon Eubank
	Anders Richardsson
	Brandon Chan
	Azher Ali
	Bashar Allabadi
	Yelizar Alturmessov
	Jared Baker
	Ann Catton
	Kim Cullion
	Daniel DeMaria
	Patrick Dos Santos
	Henrich Feher
	Vincent Ferretti
	Francois Gerthoffert
	Minh Ha
	Robin Haw
	Atul Kachru
	Alexandru Lepsa
	Alexis Li
	Rakesh Mistry
	Hardeep Nahal-Bose
	Aleksandra Pejovic
	Sam Rich
	Leonardo Rivera
	Ciarán Schütte
	Lincoln Stein
	Edmund Su
	Robert Tisma
	Jaser Uddin
	Chang Wang
	Alex Wilmer
	Linda Xiang
	Junjun Zhang
	Mélanie Courtot
	Christina Yung
**Order of Authors Secondary Information:**	
**Response to Reviewers:**	Please refer to attached Response to Reviewers letter - content copied below for reference<br><br>Response to editor:

Prof Lan: In addition, please register any new software application in the bio.tools and SciCrunch.org databases to receive RRID (Research Resource Identification Initiative ID) and biotoolsID identifiers, and include these in your manuscript. Computational workflows should be registered in workflowhub.eu and the DOIs cited in the relevant places in the manuscript. These will facilitate tracking, reproducibility and re-use of your tool.

Response to Editor: Thank you for the feedback. We have registered the components as required and added the IDs in the manuscript, under Availability of supporting source code and requirements
Section.
· RRID: SCR_026457
· Bio.tool ID: biotools:overture

Response to reviewers:

Reviewer #1: The authors present a new data platform, named Overture, for storing and browsing genomic dataset. In general, the paper is easy to read and nicely written, but it lacks major discussions on interoperability, heterogeneity management, and querying, which are not trivial when integrating genomic datasets at a large scale, as it is done in Overture. This is why I recommend a major revision, and detail it with the following points.

Response to Reviewer #1: Thank you for your careful review of the manuscript. We appreciate the time and effort you have dedicated to providing feedback, which has been valuable in improving our work. We have addressed your comments and suggestions in the manuscript and have outlined our responses below. We believe this feedback has improved both the content and presentation of our research, thank you.

Response to Major Comments:

1.I do not see a discussion on how to manage data heterogeneity, which is often present at several levels: different file formats (BAM, FASTQ, etc), different columns names, etc. I expected it when reading the sections data retrieval or data submission, and also to have it discussed in the discussion.

The Overture suite supports upload and storage of any kind of data and data model. However the reviewer is correct that if the data hosted in Overture is heterogeneous, then discovery is made harder as it can't be presented in a unified/harmonized way. To address this, in the projects we manage, we typically require a data model be mapped to by the data submitters, which requires them to curate their data into the platform model. We have added text to that effect in the data submission and discussion sections, and mention some of the Overture extensions we are working on to alleviate this issue.

2.I did not find elements regarding the querying of the data. I understand this is managed by the Arranger component but I did not find details on how to query a single dataset, multiple datasets at the same time (c.f., my previous item on how to manage heterogeneity)

Querying homogenous datasets are described in the first paragraph of our data retrieval section. As detailed above and in the updated text, when data across datasets is heterogeneous, they can be rendered into independent exploration pages. Because each data point is provided with a single unique ID in the backend, this could enable cross-querying between exploration pages. This is not yet supported in the UI, and because it is not on our medium-term roadmap further mention is out of scope for this paper. To enable querying across multiple datasets, these need to be harmonized against a shared data model.

3.I did not find either a discussion on how interoperability is managed in Overture. I see this sentence: "Overture Arranger provides shared discovery tools and interoperability with other international data portals.", but I would like to have more concrete proposals

of how Overtures ensures interoperability.

Data interoperability is thanks to the conformity of our data sets to defined schemas allowing systems to more easily be linked and interoperate. In the platforms we manage, dictionaries are carefully built to align with and reuse existing metadata standards where possible. For example, the ICGC-ARGO data model has been reused for MOHCCN (Marathon of Hope Cancer Centres Network) which means the data will be natively interoperable. We have updated the sentence pointed out by the reviewer to better reflect this. Technical interoperability is achieved when multiple Overture nodes are deployed within a single project - as described for EUCANCan in table 2 - which enables Maestro indexing to run across instances.

Response to Minor Revisions:

- Abstract : background and findings paragraphs to be justified
  - Updated accordingly
- Discussion: should be justified too
  - Updated accordingly
- Platform overview: Overture platforms are highly ... > Overture platform is highly …
  - Updated accordingly
- Data retrieval: the second paragraph could probably go before the Figure 2 image, next to the first paragraph to to loose so much space
  - Updated accordingly
- Impact: into three segments > two segments?
  - Updated accordingly
- Medium to small labs and institutions: the technical complexity ... have > has
  - Updated accordingly

Reviewer #2: This manuscript describes a data sharing system that can be utilized in various genome analysis and data sharing projects, ranging from large-scale genome analysis consortia to projects conducted by small to medium-sized research institutions. This system is based on the data portal and submission system of the International Cancer Genome Consortium (ICGC-DCC) and has been restructured into a microservice architecture to enable its use in other projects.

To promote further discoveries by facilitating the sharing and reuse of genomic data and metadata, it is essential that genomic data within online systems be properly maintained, searchable, retrievable, well-structured, and supportive of metadata and provenance tracking.

Research groups handling large-scale datasets that do not meet the requirements of existing archival resources such as GDC,SRA,EGA, or projects with datasets that do not conform to the specific data models or file formats accepted by these repositories, often face the need to build their own online data sharing systems. However, this is frequently a technical and financial challenge.

The system proposed by the authors is anticipated to be a highly effective solution, offering significant improvements in addressing these challenges while greatly enhancing accessibility, usability, and practicality for such projects.

The microservice architecture is both rationally and carefully designed, and the system is implemented using the well-established Java Spring Framework, ensuring robust security and reliability.

The manuscript's explanation of the system begins with a classification of the users who will utilize the system, and it follows a modern object-oriented development process. This approach makes the content highly comprehensible and serves as an exemplary model for similar works.

Powered by Editorial Manager® and ProduXion Manager® from Aries Systems Corporation

I believe this manuscript is all but ready for publication. However, I suggest adding explanations on a few points listed below, which would greatly enhance its utility for readers.

Response to Reviewer #2: We thank the reviewer for their positive feedback. We have addressed your comments and suggestions throughout the revised manuscript and outlined the changes below. Addressing these comments has definitely strengthened both the content and presentation of our research, thank you.

Response to Major Comments:

1.One of the key focuses of this paper is the transition to a microservice architecture, which represents a significant advancement in terms of scalability and potential for future feature expansion. Furthermore, the system is designed with high portability, not being tied to any specific cloud provider's infrastructure, and it appears to function even in on-premises environments. To aid readers in utilizing the Overture system, it would be extremely beneficial to include concrete examples of cloud IaaS configurations and on-premises hardware setups for deploying the system. For small projects, as already mentioned, deploying containers on a single server should suffice. However, for medium-to-large projects, it would be helpful to provide guidance on the intended hardware or cloud system configurations. Additionally, an estimate of the computational resources required for varying data sizes would be highly valuable to the readers.

We support users in configuring our software for production through our documentation and administration guides however we do not provide guidance on maintaining a production server due to both the rapidly evolving technological landscape and variability across deployment contexts.

While we can provide numbers based on the projects in table 2, those would only be anecdotal, which is why we instead describe the data size. Indeed, costs fluctuate based on the cloud or on-prem service provider, which would be the best to provide up-to-date cost estimates. We have added a sentence in “Availability of supporting source code and requirements” clarifying this.

2.Since the system is designed with federation in mind, and assumes long-distance communication across different jurisdictions, it would be helpful to include examples of federation setups across countries. These examples do not necessarily need to reflect existing implementations, but it would be valuable to describe the anticipated hardware or cloud configurations for such a setup.

EUCANCan described in table 2 was our first attempt at international federation. We shared only a subset of the metadata for discovery in a central node, while the genomic data remained in place at each host institution. For ICGC ARGO we are planning to provide ‘true federation’ where neither the metadata nor data move from their host institution, and rather the querying is done at each node. In both cases the hardware and cloud configuration required is (1) not necessarily different from other nodes and (2) dependent on each host institution's capabilities. As the reviewer suggests, and pending the outcome of the ethical review we mention in the discussion section, there may be a future need to adopt different cloud configurations, such as requiring geolocation of the server hosting the data. Should this be the case, this would be managed by the hosting provider rather than the Overture suite.

3.When applying this system to actual projects, it would be helpful for readers if the manuscript discussed efforts or strategies, such as training or education, to encourage adoption by data curators and resource managers, enabling them to accept and start using the system effectively.

Thank you for this recommendation. We wholeheartedly agree, and indeed since the submission of this paper we have extensively overhauled our documentation and provided direct support to users as well as provide a new online discussion forum. Based on their empirical feedback, our platform's approachability, defined by the initial ease of local setup, available documentation and support has emerged as a key

Powered by Editorial Manager® and ProduXion Manager® from Aries Systems Corporation

differentiator from our competitors. We have added text in the discussion section to highlight that this is still work in progress.

Reviewer #3: The authors present an open-source data platform that provides a generic framework to facilitate the development and deployment of genomics data applications. This software has been designed to address the growing need for data platforms tailored to the custom and diverse requirements of various scientific projects. The publication is well-structured and effectively describes the different components of Overture, making it accessible to the target audience.

As highlighted in the introduction, the increasing diversity of genomics-based projects and datasets is not yet matched by adequate software solutions for storing and sharing such data online. Challenges such as the development costs of these platforms and the short-term funding of projects often hinder the creation, reliability, and sustainability of resources. The authors tackle these issues with a modular solution, organized into configurable components.

This solution is distributed under open-source terms, and significant effort has been invested in providing extensive documentation, with commendable results. Additionally, the availability of a Docker Compose-based setup facilitates minimal local deployments, enabling new users to quickly explore the platform. These aspects demonstrate a strong commitment to usability and accessibility, enhancing the potential impact of the platform.

Response to Reviewer #3: Thank you for your positive feedback. We believe these changes outlined within our paper and below have substantially improved the manuscript's clarity and completeness. We thank the reviewer for their valuable feedback that has helped enhance the paper's quality.

Response to Major Comments

1.One of the notable strengths of Overture is its modularity, as demonstrated by the diverse component combinations outlined in Table 2, "Impact on consortium-level projects." While the ICGC ARGO project, which utilizes all components, is elaborated on in detail, it would be valuable to hear more about projects that employ only one component. Such use cases could further illustrate the platform's flexibility compared to more monolithic solutions.

For this paper we prioritized showcasing the diversity of use cases in Table 2 as well as the complexity of ICGC ARGO vs going in depth for a restricted number of applications.Most projects from Table 2 are however associated with a more comprehensive case study description in our documentation and we have now added the respective hyperlinks to Table 2, thanks for the suggestion.

2.The manuscript mentions Keycloak and Ego as authentication and authorization components. Keycloak is a third-party service, while Ego is a custom component. However, the distinction between the two is not clearly addressed. Are these options fully interchangeable? What are their respective properties, and under which criteria might one be chosen over the other for local deployments? A discussion of these aspects would enhance clarity.

We have added text in the Discussion section explaining the current support of both and future plans.

3.Another area that could benefit from elaboration is data submission from the user's perspective. While the manuscript provides a comprehensive description of the user interface for browsing and searching data, it minimally discusses the tools or processes for data submission. This is a critical aspect, as submission workflows often present significant challenges. Based on the online documentation, new components such as Lyric and Lectern are under development to assist with metadata model design and submission. Additionally, metadata submission via a command-line

	interface is already supported. Including a discussion of the existing command-line tools and ongoing developments in the manuscript's conclusion would provide a more complete picture.<br><br>As noted by the reviewer, we do have some components under development to support submission. As those are not established and production ready yet, we purposely decide not to include them under Methods. However, we do appreciate the point made by the reviewer and the usefulness of mentioning this work in progress, and have consequently added some text to the Discussion section.<br><br>Response to Minor Revisions:<br><br>●Background: The authors highlight the challenges of sharing data that do not conform to the specific data models and formats of major international platforms. While the critique of ad hoc solutions like supplementary files is valid, mention could be made of generic data publication platforms such as Zenodo or Figshare. These platforms, while not as advanced as the one described here, offer improved findability and citability compared to supplementary materials.<br>○We've updated the background to mention " general-purpose repositories such as Zenodo and Figshare" highlighting how they "provide valuable platforms for research data publication".<br>● Figure 1: Consider renaming the figure to "Platform Components Overview" for clarity. From the caption, it is not immediately evident that the entities listed represent the platform's components.<br>○We've updated figure 1 accordingly<br>● Table 1: While both Keycloak and Ego are mentioned as authentication/authorization services in the text, only Ego appears in Table 1. Clarifying the differences between these components and providing guidance on their selection would improve this section.<br>○Overture is made to be highly modular, KeyCloak is a third-party open-source identity and access management service that can be used in place of Ego. We've updated the description and table 1 content to reflect this information.<br>● Data Submission: The statement "All publication controls are facilitated by Song" could be expanded with specific examples of controls implemented on existing deployments to illustrate this point more concretely.<br>○As described in this section Songs publications controls include the commands "publish, unpublish and suppress". We've appended the sentence to read as follows "All publication controls are facilitated by Song using its publication command or endpoint."<br>● Data Administration: The phrase "outlining the structure and syntax of the data model in JSON format" could be revised for precision. Replacing "outlining" with "describing" or "specifying" better reflects the role of JSON schemas in providing detailed definitions of data models.<br>○We have updated "outlining" to "specifying"<br>● Table 2: The component referred to as the "Overture Data Management System (DMS)" in the EUCANCan project is not clearly identified elsewhere in the manuscript. Is this equivalent to Song or another component? Clarification would be helpful (Ego, Song, Score, Maestro, Arranger).<br>○The DMS included all core Overture services packaged with automated scripts for server deployments. For various reasons such as lack of scalability and configuration this Overture package was discontinued and has now been replaced with our more flexible docker setups. We have updated the components used to reflect all the services deployed rather than mentioning the DMS.<br>● "Medium to small laboratories and institutions": In the phrase "a Docker Compose that enables users to deploy…," consider replacing "a Docker Compose" with "a Docker Compose-based makefile" for greater technical accuracy (and to drive readers to use the Makefile rather than the more complicated docker-compose).<br>○We have updated the terminology to "Docker Compose-based makefile"
**Additional Information:**	
**Question**	**Response**
Are you submitting this manuscript to a special series or article collection?	No

**Experimental design and statistics**<br><br>Full details of the experimental design and statistical methods used should be given in the Methods section, as detailed in our Minimum Standards Reporting Checklist. Information essential to interpreting the data presented should be made available in the figure legends.<br><br>Have you included all the information requested in your manuscript?	Yes
**Resources**<br><br>A description of all resources used, including antibodies, cell lines, animals and software tools, with enough information to allow them to be uniquely identified, should be included in the Methods section. Authors are strongly encouraged to cite Research Resource Identifiers (RRIDs) for antibodies, model organisms and tools, where possible.<br><br>Have you included the information requested as detailed in our Minimum Standards Reporting Checklist?	Yes
**Availability of data and materials**<br><br>All datasets and code on which the conclusions of the paper rely must be either included in your submission or deposited in publicly available repositories (where available and ethically appropriate), referencing such data using a unique identifier in the references and in the "Availability of Data and Materials" section of your manuscript.<br><br>Have you have met the above requirement as detailed in our Minimum Standards Reporting Checklist?	Yes

GigaScience has policies and guidelines in place for the use of generative AI-writing tools such as ChatGPT. If you have used such writing tools to assist with writing the manuscript this must be declared and cited in the text. Authors should not list AI-writing tools and other AI-assisted technologies as an author or co-author and should acknowledge that they are fully responsible for text generated or refined by AI-writing tools.<p><br><br>A summary of use (particularly in the introduction or among methods) needs to be included at the end of the paper, and the outputs should also be included as a supplementary file hosted in GigaDB or other open repositories. Please read our guidelines for more information. <p><br><br>By submitting to GigaScience, you are aware of the journal's AI-writing tools policy, and if you have declared use of such tools below, you have acknowledged this where appropriate in your manuscript and have made a summary of use and outputs available. <p><br><b>AI-assisted writing tools have been used in the preparation of this manuscript?	No

Powered by Editorial Manager® and ProduXion Manager® from Aries Systems Corporation

Manuscript

Click here to access/download;Manuscript;Overture Paper - Manuscript - Final.docx

Overture: An Open-Source Genomics Data Platform

Authors: Mitchell Shiell[1,†], Rosi Bajari[1,†], Dusan Andric[1], Jon Eubank[1], Brandon F. Chan[1], Anders J. Richardsson[1], Azher Ali[1], Bashar Allabadi[1], Yelizar Alturmessov[1], Jared Baker[1], Ann Catton[1], Kim Cullion[1], Daniel DeMaria[1], Patrick Dos Santos[1], Henrich Feher[1], Francois Gerthoffert[1], Minh Ha[1], Robin A. Haw[1], Atul Kachru[1], Alexandru Lepsa[1], Alexis Li[1], Rakesh N. Mistry[1], Hardeep K Nahal-Bose[1], Aleksandra Pejovic[1], Samantha Rich[1], Leonardo Rivera[1], Ciarán Schütte[1], Edmund Su[1], Robert Tisma[1], Jaser Uddin[1], Chang Wang[1], Alex N. Wilmer[1], Linda Xiang[1], Junjun Zhang[1], Lincoln D. Stein[1,3], Vincent Ferretti[1,4], Mélanie Courtot[1,2,‡,#], Christina K. Yung[1,‡]

1. **Ontario Institute for Cancer Research (OICR)**
2. **University of Toronto Department of Medical Biophysics**
3. **University of Toronto Department of Molecular Genetics**
4. **Research Center of the CHU Sainte-Justine, University of Montreal**

† These authors contributed equally
‡ These authors contributed equally
# Corresponding Author

ORCID iDs: Mitchell Shiell [0000-0003-3255-6348]; Rosi Bajari [0000-0002-1402-7496]; Dusan Andric [0000-0001-7216-2330]; Jon Eubank [0009-0003-2584-4912]; Anders Richardsson [0009-0005-5879-5883]; Brandon Chan [0000-0003-2812-522X]; Azher Ali; Bashar Allabadi; Yelizar Alturmessov; Jared Baker; Ann Catton; Kim Cullion; Daniel DeMaria; Patrick Dos Santos; Henrich Feher; Vincent Ferretti [0000-0002-2555-1277]; Francois Gerthoffert; Minh Ha; Robin Haw [0000-0002-2013-7835]; Atul Kachru; Alexandru Lepsa [0009-0002-4583-6234]; Alexis Li; Rakesh Mistry; Hardeep Nahal-Bose [0000-0002-0774-2862]; Aleksandra Pejovic; Sam Rich; Leonardo Rivera; Ciarán Schütte; Lincoln Stein [0000-0002-1983-4588]; Edmund Su [0000-0003-3156-176X]; Robert Tisma; Jaser Uddin; Chang Wang; Alex Wilmer; Linda Xiang [0000-0002-1377-1125]; Junjun Zhang [0000-0001-5654-243X]; Mélanie Courtot [0000-0002-9551-6370]; Christina Yung [0000-0003-2958-150X]

Abstract

Background

Next-generation sequencing has created many new technological challenges in organizing and distributing genomics datasets, which now can routinely reach petabyte scales. Coupled with data hungry artificial intelligence and machine learning applications, findable, accessible, interoperable and reusable genomics datasets have never been more valuable. While major archives like the Genomics Data Commons (GDC), Sequence Reads Archive (SRA), and European Genome-Phenome Archive (EGA) have improved researchers' ability to share and reuse data, and general-purpose repositories such as Zenodo and Figshare provide valuable platforms for research data publication, the diversity of genomics research precludes any one-size-fits-all approach. In many cases, bespoke solutions are required and despite funding agencies and journals increasingly mandating reusable data practices, researchers still lack the technical support needed to meet the multifaceted challenges of data reuse.

Findings

Overture bridges this gap by providing open-source software for building and deploying customizable genomics data platforms. Its architecture consists of modular microservices, each of which is generalized with narrow responsibilities that together combine to create complete data management systems. These systems enable researchers to organize, share and explore their genomics data at any scale. Through Overture, researchers can connect their data to both humans and machines, fostering reproducibility and enabling new insights through controlled data sharing and reuse.

Conclusions

By making these tools freely available, we can accelerate the development of reliable genomic data management across the research community quickly, flexibly, and at multiple scales. Overture is an open-source project licensed under AGPLv3.0 with all source code publicly available from https://github.com/overture-stack and documentation on development, deployment and usage available from www.overture.bio.

Keywords: Research Software, Data Management, Genomics, open-source, open-science

First	Last	Preferred Email	Authorship
Mitchell	Shiell	mshiell@oicr.on.ca	Co-First Author
Rosi	Bajari	rosi.bajari@gmail.com	Co-First Author
Dusan	Andric	dusan.andric@gmail.com	Co-Author
Jon	Eubank	jeubank@oicr.on.ca	Co-Author
Anders	Richardsson	jrichardsson@oicr.on.ca	Co-Author
Brandon	Chan	bchan@oicr.on.ca	Co-Author
Azher	Ali	a2ali@oicr.on.ca	Co-Author
Bashar	Allabadi	basharlabadi@gmail.com	Co-Author
Yelizar	Alturmessov	yalturmessov@oicr.on.ca	Co-Author
Jared	Baker	jbaker@oicr.on.ca	Co-Author
Ann	Catton	acatton@oicr.on.ca	Co-Author
Kim	Cullion	kimcullion@gmail.com	Co-Author
Daniel	DeMaria	ddemaria@oicr.on.ca	Co-Author
Patrick	Dos Santos	pdossantos@oicr.on.ca	Co-Author
Henrich	Feher	hfeher@oicr.on.ca	Co-Author
Vincent	Ferretti	vincent.ferretti.hsj@ssss.gouv.qc.ca	Co-Author
Francois	Gerthoffert	fgerthoffert@gmail.com	Co-Author
Minh	Ha	hlminh2000@gmail.com	Co-Author
Robin	Haw	robin.haw@oicr.on.ca	Co-Author
Atul	Kachru	atul.kachru3845@gmail.com	Co-Author
Alexandru	Lepsa	lepsalex@gmail.com	Co-Author
Alexis	Li	alexis.li@oicr.on.ca	Co-Author
Rakesh	Mistry	rmistry@oicr.on.ca	Co-Author
Hardeep	Nahal-Bose	hnahal@oicr.on.ca	Co-Author
Aleksandra	Pejovic	pejovicaleks@gmail.com	Co-Author
Sam	Rich	srich@oicr.on.ca	Co-Author
Leonardo	Rivera	lrivera@oicr.on.ca	Co-Author
Ciarán	Schütte	cschutte@oicr.on.ca	Co-Author
Lincoln	Stein	lstein@oicr.on.ca	Co-Author
Edmund	Su	esu@oicr.on.ca	Co-Author
Robert	Tisma	rtisma@gmail.com	Co-Author
Jaser	Uddin	udjaser@outlook.com	Co-Author
Chang	Wang	garnwraly@gmail.com	Co-Author
Alex	Wilmer	djazium@gmail.com	Co-Author
Linda	Xiang	lxiang@oicr.on.ca	Co-Author
Junjun	Zhang	junjun.ca@gmail.com	Co-Author
Mélanie	Courtot	mcourtot@gmail.com	Co-Senior Author<br>Corresponding Author
Christina	Yung	christina.k.yung@gmail.com	Co-Senior Author

Background

Genomics research has benefited from a strong tradition of open science principles that have fostered comprehensive studies, transparent results, and accelerated scientific discovery[1]. As sequencing costs decrease, large and small research groups are increasingly generating massive multi-omics and single-cell data sets[2], often combined with clinical and imaging data[3]**.** This data abundance coincides with the emergence of machine learning (ML) and artificial intelligence (AI)[4], the biggest data-consuming activities in history. These emerging fields have sprouted from the wide availability of data; however, their solutions are limited by the quality of relevant data openly available for consumption[5]. Our brave new world now demands readily available software infrastructure to collect, organize, and share data.

In response, funding agencies and academic journals increasingly insist that projects generating large amounts of sequencing data respect FAIR (Findable, Accessible, Interoperable and Reusable) data practice[5]. This shift reflects a growing recognition and expectation of the researcher's role in facilitating data reuse. However, sharing genomics data is a multifaceted challenge:

- The volume of data often requires researchers to use cloud-based solutions that introduce new costs and expertise.[6]
- Sharing data across the research community must be done in an interoperable and sustainable fashion.[7]
- The legal, ethical, and social implications of genomics data sharing, including ownership, sovereignty, and data misuse, are extensive and evolving.[8-9].

While several resources for depositing genomic data, such as the Genomic Data Commons (GDC)[10], Sequence Read Archive (SRA)[10], and European Genome-Phenome Archive (EGA)[12], take on much of the responsibility of managing and archiving data, not every project is eligible to submit data to these repositories. The GDC focuses on human cancer genomes and is limited to DNA and RNA sequencing data in FASTQ and BAM formats[13]. The EGA only accepts human genomic and phenotypic data[14]. Alternatively, the SRA accepts a wide range of sequencing data types and is agnostic to the organism of origin[15]. However, like all archival resources, it faces fundamental challenges in accommodating niche and rapidly evolving data requirements[16]. This presents a serious challenge to research groups with large volumes of data that do not meet the requirements of existing archival resources, and projects with datasets that do not conform to the specific data model and file types accepted by these repositories get left out. Instead, these results must be shared ad hoc, such as in publication supplementary data files. To meet FAIR standards, genomic data must be maintained in an online system that allows for search and retrieval, is well-structured, and supports metadata and provenance tracking. Few research groups have the expertise to implement such a system[17], leaving groups with the choice of building the expertise in-house or hiring outside consultants, developers, and IT support staff. The first solution is inefficient, and the second one frequently exceeds available funding.

We created Overture[18-19] to enable researchers to build and deploy reproducible large-scale data platforms. With these platforms, researchers can maximize the potential of existing research, encouraging transparency, reproducibility, and reuse of data while maintaining oversight over its distribution. Other researchers can see their results, explore the data underpinning them, and reuse them to drive further discovery.

Results

Development of Overture

Overture was built based on a data portal and submission system developed to support the Data Coordination Center of the International Cancer Genome Consortium (ICGC-DCC)[20-21], a popular cancer genomics resource covering 84 worldwide projects, and molecular data from over 24,000 patients. The ICGC-DCC platform provided researchers with a user-friendly interface for efficient access, visualization, and analysis of its genomic data. After its launch, the portal's data exploration and analysis capabilities attracted attention from various research groups. Several of these groups successfully re-implemented and adapted the ICGC-DCC's infrastructure, including the Hartwig Medical Database[22] and the Translational Human Pancreatic Islet Genotype tissue-Expression Resource Data Portal (TIGER)[23].

However, the reusability of the ICGC-DCC infrastructure was met with significant technical challenges, particularly tied to its monolithic architecture and numerous hard-coded elements, which made it unnecessarily difficult to replicate and implement the system in projects with similar needs, and limited the ability to scale in production. These limitations prompted a strategic shift toward a more flexible and scalable microservice architecture. This was chosen for several key advantages; *(1) Scalability:* microservices enable individual system components to scale independently. *(2) Flexibility:* each microservice can be deployed and upgraded separately, easing the development of new features and modifications to existing ones. *(3) Resilience:* if one microservice encounters a failure, other instances reduce or even avert downtime by load balancing accordingly. This resulted in the development of Overture, a collection of reusable and modular microservices that serve as a general solution for building and deploying data platforms.

Platform Overview

The Overture platform is highly flexible yet fundamentally has a standard feature set provided by its core software components (Figure & Table 1). The target users for our data platform's core functionalities can be divided into three categories:

1. Data consumers retrieving data from the platform.
2. Data providers submitting data to the platform.
3. Data administrators who configure and maintain the platform.

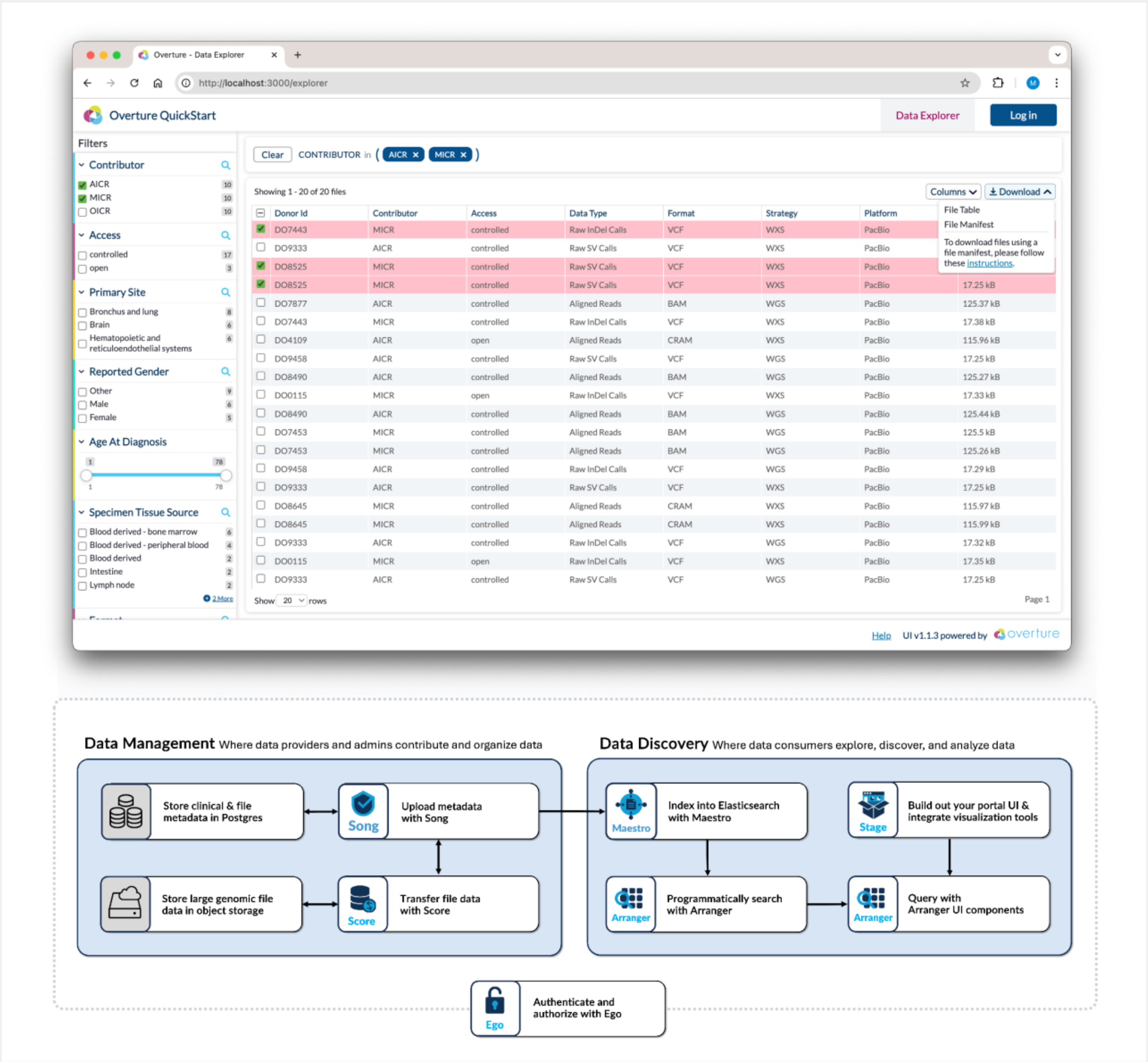

Figure 1. Platform Components Overview: On the front-end, Stage[24] provides the basic user interface (UI), including navigation menus; as well as data exploration, login and profile pages. Arranger's[25] library of search UI components then integrates with Stage to offer a configurable search facet panel, data table, and filter summary panel. Login and profile pages integrate with Keycloak[26] or Ego[27], which provides authentication and authorization for users and applications. Behind the scenes, Song[28] and Score[29] facilitate data management, retrieval and submission. Score transfers large genomic files to and from S3-compatible object storage, while Song stores and handles the files' metadata. These databases are indexed by Maestro[30] into unified Elasticsearch[31] file-centric and analysis-centric indices. Arranger then uses these to produce a GraphQL[32] search API that connects with its front-end library components on the data exploration page. Combined together, these services broadly enable the secure and scalable reuse of genomics data.

.

Table 1. Overview of Overture Stack Software Components: Overture comprises six core components and one third-party component, that work in concert to create genomics data management systems.

Product Name	Code repository	Brief description
Song	https://github.com/overture-stack/song	Metadata management with an automated submission validation system.
Score	https://github.com/overture-stack/score	File Transfer Microservice that supports fault-tolerant multi-part parallel transfer
Maestro	https://github.com/overture-stack/maestro	Indexes metadata from Song into Elasticsearch search indices, to be consumed by Arranger.
Arranger	https://github.com/overture-stack/arranger	Data search and exploration API, and accompanying library of UI components that can be easily integrated in a data portal.
Ego	https://github.com/overture-stack/ego	OAuth 2.0 authorization service that supports multiple OpenID Connect (OIDC) identity providers.
Keycloak	https://github.com/keycloak/keycloak	A popular third-party open-source identity and access management service.
Stage	https://github.com/overture-stack/Stage	A React-based user interface designed to allow easy deployment of browser-friendly data portals.

Data Retrieval

Data retrieval starts from the Stage data exploration page (figure 2), where users can filter data using the Arranger search facets. These enable rapid and efficient data filtering using checkboxes, date ranges, sliders, and quick search input boxes, allowing users to narrow their queries and focus on relevant data subsets. Filtered datasets are presented in the Arranger data table, which provides sortable columns, file counts, and pagination. All query parameters are summarized within an Arranger filter panel at the top of the page, giving users a clear overview of their search criteria. Users can easily share these queries using the browser URL, which gets updated with the filter parameters in real-time.

Once the users have identified relevant data, they can select the download dropdown, which provides options for downloading metadata or a file manifest in a TSV format. The manifest file allows users to download their files of interest directly from the resources database and object storage using Overture's command line interface (CLI) tools, specifically the Song and Score clients. These CLI tools are needed as massive genomic datasets require reliable multi-part parallel download sessions unsuitable for a browser. To ensure secure access to data, users must supply a valid API key when installing the Song and Score clients. For controlled-access data, researchers will be able to retrieve their API key after their data access request is granted by typically the relevant data access committee. Log in to the web portal is facilitated by either Keycloak or Ego, which supports popular identity providers, including Google, ORCiD, and GitHub.

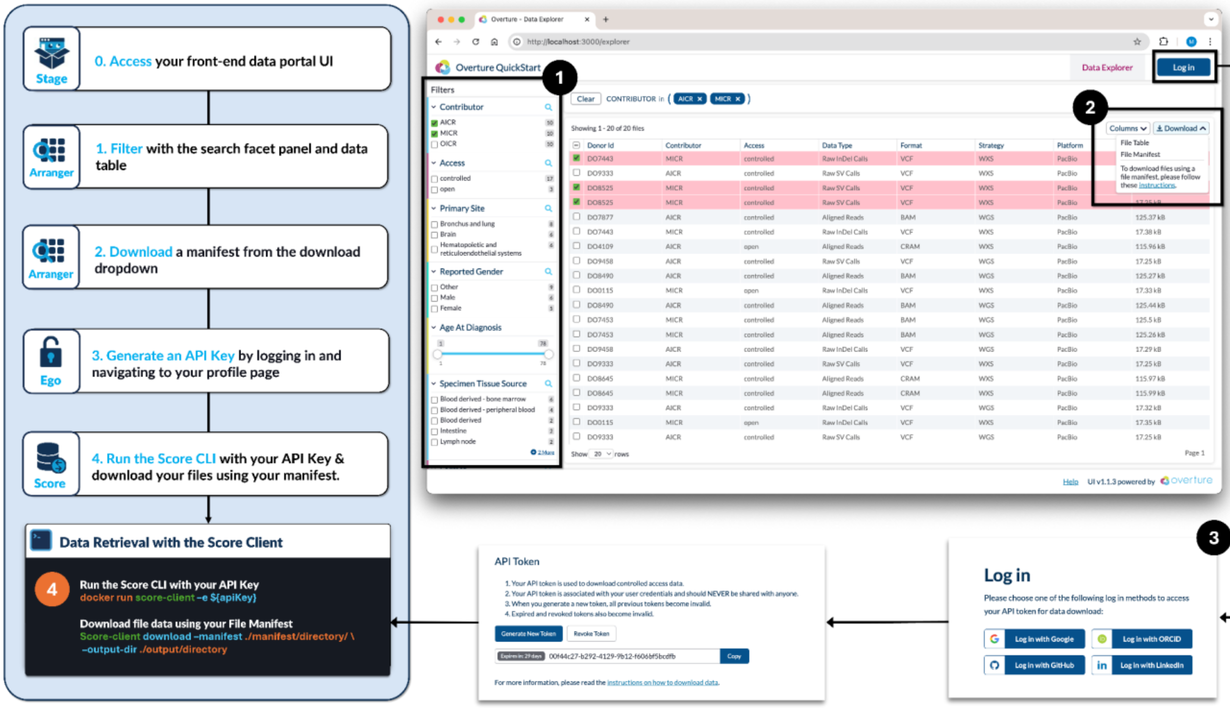

Figure 2. Data Retrieval Workflow: Users first filter data via Arranger's search components in the Stage UI's data explorer. Once they have selected a subset, they then download a 'file manifest' from the download dropdown. To access Song and Score data, users log in through Stage's auth integration and obtain their API key from the profile page. This API key is provided when installing the score client. Finally, files are downloaded to the user's device using the Score client's download command, specifying the file manifest and desired output directory.

Data Submission

Overture's submission process has been designed to ensure data integrity by facilitating data tracking and data model compliance. In Overture, a set of one or more files plus the metadata describing that collection of files is called an analysis. To upload an analysis, data submitters first organize their metadata files, typically using a spreadsheet editor alongside a data dictionary supplied by the resource administrator. To provide a unified view of the metadata in the Overture exploration table, submitted data must be curated against the chosen metadata model. Alternatively, users can decide to create multiple exploration tables for independent and heterogeneous datasets. The data dictionary describes the required metadata fields and the expectations for the syntax of each field. Once converted to JSON, the Song Client upload command can be used to send the metadata submission to the resources Song server for validation against the admin defined data model. If there are any issues with the metadata, the user will be provided a detailed error message. If successful, the user will be provided a success message and an auto-generated analysis ID for future reference within the system.

After submitting metadata and establishing an analysis ID, the submitter uploads file data using Song and Score clients. First, a file manifest is generated using the Song client manifest command, along with the directory where the files are located and the analysis ID assigned to the relevant metadata. This links the uploaded files to the metadata in Song's database. The manifest and Score Client upload

command is then used to transfer files to object storage. Once uploaded, Song can dynamically update file metadata, such as md5 checksums, to the appropriate analysis file within its database.

All data uploaded to the resource are, by default, in an unpublished state. Publication controls allow administrators and data providers to coordinate and prepare data releases in a predictable and timely manner. All publication controls are facilitated by Song using its publication command or endpoint. When data is ready for search and download, administrators can make it available by updating the desired analyses to a published state. If data is no longer relevant, the data administrators can make them unavailable to downstream services by setting analyses to a suppressed state.

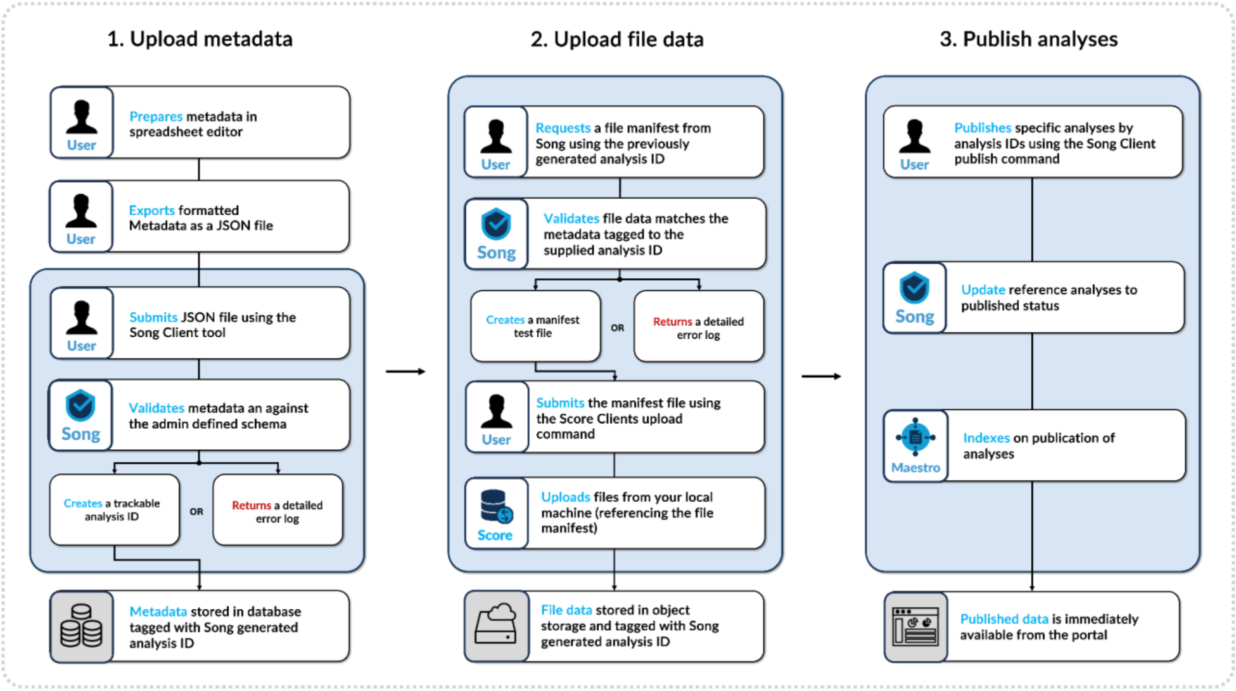

Figure 3. Data Submission Workflow: Overture's submission process enhances data integrity with data tracking and data model adherence. It involves organizing metadata files, converting them to JSON, and uploading via the Song Client for validation. Successful submissions receive an auto-generated analysis ID. File data is then uploaded using Song and Score clients, generating a file manifest linked to the metadata. All data starts unpublished and is managed through Song's publication controls for coordinated data releases.

Data Administration

Data administrators are responsible for maintaining and configuring the data resource including providing the data model, configuring the portal search interface, and managing users' access and permissions. With Overture, administrators have the flexibility to define the data model of their resources according to their desired standards. This involves specifying the structure and syntax of the data model in JSON format. Once the JSON schema is created, the administrator can submit it to Overture's Song microservice through the Song API. This schema then serves to verify all future submissions to the resource. Multiple schemas can be registered to the platform, enabling validation for various data types.

Given the potential diversity of projects that can use Overture, administrators require flexibility in customizing their portal front-end interface. With Arranger's library of UI components, among other customizations, administrators can configure the display settings of the search facets and data table on the portal exploration page. This includes configurations for what fields are visible, how they are displayed, and how users can interact with them.

Admins also have the flexibility to customize the portal's content by building on top of the Stage UI. Stage is a React-based single-page web application designed to provide a foundation for building any data portal. Its default features include a header, footer and navigation menus. With functional knowledge of React, you can easily theme and extend Stage to include custom pages and menu options, such as funding acknowledgments, documentation, and data release statements.

Depending on the authorization service chosen, admins can manage user permissions through the Ego admin UI or KeyCloak. These allow admins to apply role-based permissions granting read or write access to users and applications through JSON Web Tokens (JWTs) or API Keys.

Impact

The types of groups that can deploy Overture platforms can be categorized into two segments based on the scale of data and their level of in-house expertise:

1. **Large-scale Consortiums** with tens to hundreds of thousands of samples, and technical staff to support the expansion of both hardware and software infrastructure. They may also need to distribute data across multiple locations and jurisdictions.
2. **Medium to small-sized labs, programs and institutions** with hundreds to thousands of samples and limited system administration support.

We will address each of these groups in the following sections.

Impact on Consortium-level Projects

Multiple consortium-level projects have successfully reused Overture components, reducing development efforts and, in turn, driving further enhancement. As a testament to the benefits of a modular approach, Overture has significantly impacted several consortium-level projects (Table 1).

Case Study ICGC-ARGO

ICGC Accelerating Research in Genomic Oncology (ARGO)[33] is a global initiative to provide precision oncology knowledge. Intending to analyze genomes from 100,000 cancer patients, ICGC-ARGO aims to collect genomic data alongside high-quality clinical data and make it available to the research community quickly and responsibly.

ICGC-ARGO operates at a global scale. To satisfy the legal requirements of data sovereignty, ARGO needs to implement a distributed network of servers or nodes located within each country of data origin. Thanks to Overture, the ARGO development team is deploying a global network of interoperable regional data processing centers (RDPCs) where data is submitted and stored within each country of origin. Each RDPC leverages the core Overture components - Song, Score and Maestro. The ARGO platform

then federates queries through an Arranger server, enabling search across the global network of RDPC nodes from the ARGO Data Platform (https://platform.icgc-argo.org/) .

Table 2. Impact on consortium-level projects: an overview of consortium-level data-sharing initiatives. Each initiative briefly describes the project and the Overture components that help drive it.

Project	Description	Component(s) used	Data
**Kids First Data Resource Portal**[34]	The Kids First Data Portal provides access to genomic and clinical data sets generated by the Kids First Pediatric Cancer, the Rare Diseases Data Resource and other NCI-supported pediatric genomics projects. The data sets are stored in a secure, centralized repository and made available worldwide to researchers and the public. For more information, see our case study page (https://www.overture.bio/case-studies/#kidsfirst)	Arranger	34,000 Human Genomes
**Human Cancer Models Initiative (HCMI) Searchable Catalog**[35]	The HCMI catalogs cancer models alongside clinical, biospecimen, and molecular data. This data-sharing platform also includes protocols, informed consent templates, and clinical data forms, making it an all-in-one resource for translational cancer researchers. For more information, see our case study page (https://www.overture.bio/case-studies/#HCMI)	Arranger	307 Cancer Models
**International Health Cohorts Consortium (IHCC) Cohort Atlas**[36]	The IHCC is improving clinical care and population health by aggregating large genomic data cohorts to help translational researchers uncover the biological and genetic factors of disease. The *IHCC Cohort Atlas* is the global data-sharing platform hosting genomics data from large cohorts (100k+). **Using a single data model unifies international cohort data and enables discovery through Arrangers search functionalities.** For more information, see our case study page (https://www.overture.bio/case-studies/#IHCC)	Arranger	Human cohort metadata for 34 Million participants
**International Cancer Genome Consortium - Accelerating Research in Genomic Oncology (ICGC ARGO)**[37]	ICGC-ARGO is a global initiative to provide precision oncology knowledge to the world. With the goal of analyzing genomes from 100,000 cancer patients, ICGC-ARGO aims to collect genomic data alongside high-quality clinical data and make it available to the research community in a rapid and responsible way. **(Re)use and extension of Overture components supports controlled data storage and access.** For more information, see our case study page (https://www.overture.bio/case-studies/#ARGO)	Ego, Song, Score, Maestro, Arranger	37,222 Genomic files
**VirusSeq Data Portal**[38,39]	The VirusSeq Data Portal is an open-source and open-access data portal for all Canadian SARS-CoV-2 sequences as well as associated non-personal contextual data. It harmonizes, validates and automates submission to international databases. **Using Overture, the portal was created within a 4 week timeframe.** Initially intended to store 150,000 sequences it has scaled to host over 500,000 genomes. For more information, see our case study page (https://www.overture.bio/case-studies/#virusseq)	Ego, Song, Score, Maestro, Arranger	98,266 Genomic file

**European-Canadian Cancer Network (EUCANCan)**[40]	The EUCANCan project offers a novel solution to managing and sharing cancer genomic data. **Overture's Maestro enables federated search across 3 EUCANCan nodes.** Instead of consolidating data in one control center following some established process and timeline, each data node manages their own data locally. The nodes agree on the set of metadata that can be queried in a unified data portal that will then point to the location of the genomic data.	Ego, Song, Score, Maestro, Arranger	N/A
**Ontario Hereditary Cancer Research Network (OHCRN)**[41]	OHCRN aims to harmonize Information from individuals with Hereditary Cancer Syndrome in order to better understand and advance the prevention, early detection and treatment of these cancers.	Arranger	N/A
**African Pathogen Data Sharing Archive (APA)**[42]	This data-sharing platform is being developed to enable real time pathogen genomics sharing and exchange across Africa[43]. The portal allows users to upload, share, explore, and download pathogen sequences and associated metadata as per data use guidelines provided by each country. **Reusing Overture has enabled LMICs institutions to build local capacity and deploy their own platform.**	Ego, Song, Score, Maestro, Arranger	N/A

Medium to small laboratories and institutions

Overture has demonstrated its efficacy for large-scale genomics data platforms, with successful deployments across many projects. However, it is important to acknowledge that the projects presented so far only include consortium-level projects. While microservice architectures offer numerous advantages, the technical complexity of deploying our platforms has been a significant barrier for small and medium sized groups. To lower adoption barriers, we've identified and addressed three fundamental questions: How can potential new users see our platform in action? How can they openly experiment with the platform? And how can they take ownership of it?

To address the first question, we developed an Overture demo portal (https://demo.overture.bio/). This environment is accessible directly from our homepage and offers new and prospective users an immediate, interactive introduction to Overture's capabilities. The demo features a representative mock dataset on the exploration page and includes supplementary content within the portal's Stage UI, providing a surface level overview of the platform's functionality.

To facilitate open experimentation of our platform, we introduced the Overture Quickstart, a Docker Compose-based[43] makefile that enables users to deploy the entire platform locally within minutes, complete with pre-populated mock data and a pre-configured admin user. To accompany our localized Quickstart setup we expanded our documentation to include platform guides that cover essential processes such as data submission, download, and core administrative tasks required for configuring an operational Overture platform.

To address ownership, we containerized and standardized the installation process for our microservices. Each service can now be installed using Docker and an environment variable file, ensuring broad portability across diverse computing environments. Furthermore we now provide a comprehensive end-to-end deployment guide, which meticulously details each stage, service, and environment variable

required for establishing a base Overture platform. Moving forward we hope to provide guides and resources for automated deployments of a variety of ideal and reproducible environments leveraging popular toolings like Terraform[44] and Helm[45]. Through these initiatives, we hope to significantly reduce barriers to adoption, making Overture more accessible to a broader range of research groups, regardless of their scale or technical expertise.

Discussion

As the software engineering team at OICR, we build data platforms with a diverse range of requirements. When our solutions prove widely applicable, they are refined into more generic tools and distributed as part of the Overture Suite. In the following sections, we will discuss some of our current challenges and how they are guiding the expansion of the Overture suite.

Recent data protection laws including the General Data Protection Regulation (EU GDPR)[46] and the Protection of Personal Information Act (POPIA) in South Africa[47] have created a shift in how we manage data across borders. Where data could formerly be transferred across jurisdictions, the current data protection laws prohibit this. Instead, we must host data in its geolocation of origin, deploying instances of the original platform in each country. This approach - *federation* - requires new means to discover the data at each node of the network instead of relying on centralized indexes. In response, we are improving our search API service Arranger, to aggregate search results across different nodes of arranger instances enabling users to query datasets from other countries of origin while still maintaining the privacy of the individual. The extent to which the data can be aggregated and further explored centrally will need to be reviewed by ethics experts; our initial foray in the area has led to different interpretations and variable willingness to share data. Clear guidance from policy and legal experts will be required to achieve our vision of building a truly federated platform; we are tackling this through collaboration with the ICGC-ARGO ethical working group[48], as well as the GA4GH Regulatory & Ethics Work Stream (REWS)[49].

The management of patient consent and controlled access to data has become a standard requirement for large-scale human genomics platforms. In the past, to gain access to data, researchers were required to submit paper and PDF forms. This process can take months and has contributed to significant barriers to data access[7]. In response to challenges in controlled data access, we created an online application module for the ICGC-ARGO project. The Data Access Committee Office (DACO) application enables researchers to log in to an online portal, fill in, sign their application, and send it for review electronically to the Data Access Committee. The Data Access officer reviews applications through an online dashboard through which they can request more information or approve/deny the applications. This process has reduced the average approval time from four weeks to 3.5 days for over 400 applications across 35 countries. For patient consent, we are building a virtual patient enrollment portal for the Ontario Hereditary Cancer Research Network. Designed to address real world project demands, the patient consent portal enables study participants to provide consent and agree to online data sharing in both an ethical and accessible manner. Patient consent and controlled access to data are core requirements for platforms handling sensitive human data. Therefore, we are working to incorporate these two applications - the DACO system and the virtual patient enrollment portal - as new Overture components.

Overture's development has been limited until recently for use on cancer genomics data. However, we are finding an increasing demand for projects that require data outside the context of cancer genomics[38,39,42]. In response, we are developing an updated data-agnostic tabular submission system to complement our existing infrastructure. This update allows us to cater to a broader range of use cases, such as pathogenic data, without additional development. Supporting additional data types from these

diverse use cases, comes the challenge of data heterogeneity. Managing this is currently beyond the scope of Overture, and the data must be curated prior to submission for harmonized discovery against the chosen data model. We are investigating ways to semi-automate this mapping of datasets against the platform data model using large language models and natural language processing methodologies, and if successful will propose this as an added Overture module for data harmonization.

Our microservices architecture allows for flexible adding and also retiring components when needed. For example, Ego is a bespoke Overture component to manage user authentication and authorization. Since its development, Keycloak has emerged as the industry standard open-source technology for authentication and authorization. Consequently, and while we currently support both Ego and Keycloak as described above, we are moving forward with deprecating Ego. This flexibility and ability to swap components means we can focus resources where needed, towards adding new functionalities and not duplicate work. As another example of this modularity,, and in addition to modules described earlier for controlled access, data harmonization and federation, our expanding user base requires better submissions support, in particular for non-technical adopters. In response, we have started development of UI-based tools to improve platform usage and engagement, such as submission UIs, built in documentation components, and a generic dictionary viewer. The Overture suite will continually evolve to address users needs - both in terms of software and documentation. Our newly developed quickstart resource has enabled us to establish a broader base of reference users. Based on their feedback we are developing new resources to take them from planning to development and into production. These improvements to our onboarding experience will in turn promote further feedback from users, providing us the clarity and direction needed to drive greater success, adoption and expansion of our software suite. As Overture has successfully delivered on its goal to enable engineering teams to build and deploy reproducible large-scale data platforms that broadly enable FAIR data discovery and reuse. An upcoming focus of our work will be working towards making data platform development accessible to research teams with limited resources, reducing initial barriers, so teams can do more with less.

Conclusions

The rapid expansion of genomics research and the intricate challenges of organizing and distributing its data present formidable obstacles for the field of genomics. Overture addresses this by offering software tools designed to build and deploy data platforms capable of efficiently managing and disseminating vast genomic datasets. Overture’s ability to fit in as a general solution for various collaborative efforts showcases its unique potential as a cornerstone in genomic research infrastructure. With Overture, we are working towards a future where opportunities for scientific discovery and innovation are no longer bottlenecked by challenges in the collection, storage and sharing of genomics data.

Methods

Development Methodology

The Overture team uses agile development practices to design, plan, and implement our software. Feature requests and bug reports are documented through GitHub issues and reviewed during monthly planning sessions. All tickets are documented, tracked, and prioritized through ZenHub. Developers peer-review "pull requests" and test them in our team's QA environments. All Overture source codebases are currently licensed under the GNU Affero General Public License v3.0.

Availability of supporting source code and requirements

Overture GitHub repository
Project name: Overture
Project home page: https://www.overture.bio/
Github link: https://github.com/overture-stack
Project documentation: https://docs.overture.bio/
Demo environment: https://demo.overture.bio/
Operating system(s): Platform independent
Programming language: : Typescript, JavaScript, Java
License: AGPL-3.0
Bio.tools Unique Identifier: biotools:overture
RRID: SCR_026457
Other requirements:

- Docker Engine 19.03+ (or equivalent open-source alternatives)
- PostgreSQL database (for Songs)
- S3-compliant object storage (for Score)
- Elasticsearch 7.10+ or open-source equivalent (for Arranger)
- Node and or Maven required for development

Software Heritage PIDs:

- Arranger: swh:1:snp:d13c689513fb9e4dbf9978acc983b7251389ee52
- SONG: swh:1:snp:4527c299e4d0fa7167e30d901f58e25167b7b097
- Maestro: swh:1:snp:94dd1f9726979920c141c5f72e72424e4b71fd6d
- Stage: swh:1:snp:84d73e6e53be322f8443db17958bedba6a7c4693
- Score: swh:1:snp:ff210042483f6f25ab1408012b66c84816683863
- Ego: swh:1:snp:f34d155d92d46c1737da9293c6fb47af17898dfc
- Keycloak (third party tool): swh:1:snp:b6c1f520311456de88d94437a080495ffbad78f7

Overture microservices can be run as individual virtual containers, requiring Docker Engine version 19.03+ (or equivalent open-source alternatives). These microservices are compatible with Linux, Mac (Intel and Apple Silicon), as well as Windows platforms. Users can deploy and access all services locally (limited to HTTP) or externally by using custom domains that support HTTPS via TLS/SSL. All necessary configurations, including integration with other Overture microservices, are provided via environment variables. Due to variability in technologies and deployment contexts we do not provide general guidance on maintaining production servers including cost estimates. Documentation, including installation, configuration and usage guides, can be found at https://www.overture.bio/getting-started/.

An Overture demo environment can be accessed from our website at https://demo.overture.bio/. We have also provided a QuickStart alongside platform guides for those interested in getting hands-on experience using our platform.

List of abbreviations

African Pathogen Data Sharing Archive (APA)
Artificial Intelligence (AI)
Command Line Interface (CLI)
Data Access Committee Office (DACO)

Data Coordination Center of the International Cancer Genome Consortium (ICGC-DCC) European-Canadian Cancer Network (EUCANCan)
Findable, Accessible, Interoperable, Reusable (FAIR)
Global Alliance for Genomics and Health (GA4GH)
GA4GH Regulatory & Ethics Work Stream (REWS)
General Data Protection Regulation (EU GDPR)
Genomic Data Commons (GDC)
Human Cancer Models Initiative (HCMI)
ICGC Accelerating Research in Genomic Oncology (ARGO)
International Health Cohorts Consortium (IHCC)
JSON Web Tokens (JWTs)
Machine Learning (ML)
Ontario Hereditary Cancer Research Network (OHCRN)
OpenID Connect (OIDC)
Protection of Personal Information Act (POPIA)
Regional Data Processing Centers (RDPCs)
Sequence Read Archive (SRA)
Translational Human Pancreatic Islet Genotype Tissue-Expression Resource Data Portal (TIGER)
User Interface (UI)

Declarations

Ethics approval and content to participate

Not applicable

Consent for publication

Not applicable

Data Availability

Overture microservices are open-source and freely available under the AGPL-3.0 license from the Overture GitHub organization, https://github.com/overture-stack/.

Competing interests

The authors declare that they have no competing interests.

Funding

National Cancer Institute, Advanced Development of Informatics Technologies for Cancer Research and Management (U24 Clinical Trial Optional), #U24CA253529, M Courtot, L Stein, C Yung;

Canadian Institutes of Health Research, Operating Grant: Coronavirus Variants Rapid Response Network (CoVaRR-Net) Extension, 175622, , M Courtot; Langlois, M; Abraham, N; Behr, M A; Brouwers, M C;

Crawley, A M; Flamand, L; Gingras, A; Gommerman, J L; Grimshaw, J M; Hsu, A T; Huyser, K R; Manuel, D G; Muhajarine, N; Ragoussis, I; Rasmussen, A; Shapiro, J;

Genome Quebec, COVID-19 Regional Genomic Initiative, PT#91095 Fund#257627, Bourque, G; Stein, S; Hsiao, W; Brinkman, F;

Canada Foundation for Innovation, Discovery Frontiers: Advancing Big Data Science in Genomics Research, CFI#32586 UofTFund#496447, Stein, L;

Canadian Institutes of Health Research, Team Grant: Pan-Canadian Human Genome Library, HGA-190675, Bourque, G; Boycott, K M; Scherer, S W; Bhérer, C; Brazas, M D; Brudno, M; Caron, N R; Courtot, M; Ferretti, V; Joly, Y; Jones, S; Lerner-Ellis, J P; Stedman, I; Stein, L; Wasserman, W W; Zawati, M H M.;

CANARIE, Research Software Program, L Stein; C Yung;

The Ontario Institute for Cancer Research is supported by funds provided by the Government of Ontario.

Authors' contributions:

All authors read and approved the final manuscript.

M.C., C.Y. contributed to all stages of the project's lifecycle, from inception to completion, formulating overarching goals and aims, acquiring funding and reviewing and editing the manuscript. L.S., V.F. contributed to multiple stages of the project's life cycle, particularly in its early stages by formulating overarching goals and aims and acquiring funding for development. H.N-B., E.S., L.X., A.A., B.A., Y.A., J.B., A.C., K.C., D.D., P.D.S., H.F., A.Li., R.M., S.R., L.R., C.S., J.U., A.W., J.Z. developed and implemented core features for the Overture project, contributing to its open-source codebase and enhancing system functionality. J.E., A.R., D.A. and A.Lepsa., designed the high-level system architecture and technical framework while contributing to the open-source codebase and enhancing overall functionality.F.G. and R.H. contributed to the project conceptualization, helping shape its core vision and objectives while also managing and coordinating project activities, ensuring alignment with goals and timelines. B.C., A.K. and A.P. and A.Li. worked on Overture as key administrators, overseeing software development lifecycle from planning to execution. The manuscript was written by M.S. and R.B. with reviews and edits primarily contributed by J.E., A.R., B.C., R.H., M.C., C.Y., L.S., V.F.

Acknowledgements

Not applicable

References

1. Gates, A. J., Gysi, D. M., Kellis, M. & Barabási, A.-L. A wealth of discovery built on the Human Genome Project — by the numbers. *Nature* **590**, 212–215 (2021).
2. Stephens, Z. D. *et al.* Big Data: Astronomical or Genomical? *PLoS Biol* **13**, e1002195 (2015).
3. Subramanian, I., Verma, S., Kumar, S., Jere, A. & Anamika, K. Multi-omics Data Integration, Interpretation, and Its Application. *Bioinform Biol Insights* **14**, 117793221989905 (2020).
4. Sharma, A., Lysenko, A., Jia, S., Boroevich, K. A. & Tsunoda, T. Advances in AI and machine learning for predictive medicine. *J Hum Genet* (2024) doi:10.1038/s10038-024-01231-y.

5. Wilkinson, M. D. et al. The FAIR Guiding Principles for scientific data management and stewardship. Sci Data 3, 160018 (2016).
6. Tanjo, T., Kawai, Y., Tokunaga, K., Ogasawara, O. & Nagasaki, M. Practical guide for managing large-scale human genome data in research. J Hum Genet 66, 39–52 (2021).
7. Powell, K. The broken promise that undermines human genome research. Nature 590, 198–201 (2021).
8. Rehm, H. L. et al. GA4GH: International policies and standards for data sharing across genomic research and healthcare. Cell Genomics 1, 100029 (2021).
9. Byrd, J. B., Greene, A. C., Prasad, D. V., Jiang, X. & Greene, C. S. Responsible, practical genomic data sharing that accelerates research. Nat Rev Genet 21, 615–629 (2020).
10. National Cancer Institute. Genomic Data Commons. https://gdc.cancer.gov/ (accessed July 24, 2024).
11. National Center for Biotechnology Information. Sequence Read Archive. https://www.ncbi.nlm.nih.gov/sra (accessed July 24, 2024).
12. European Genome-phenome Archive. https://ega-archive.org/ (accessed July 24, 2024).
13. National Cancer Institute. Genomic Data Commons Data Submission Process. https://gdc.cancer.gov/node/216/ (accessed July 24, 2024).
14. European Bioinformatics Institute. EGA Quick Tour: Submitting data to EGA. https://www.ebi.ac.uk/training/online/courses/ega-quick-tour/submitting-data-to-ega/ (accessed July 24, 2024).
15. National Center for Biotechnology Information. SRA Submission Format. https://www.ncbi.nlm.nih.gov/sra/docs/submitformats/ (accessed July 24, 2024).
16. Leigh, D. M. et al. Best practices for genetic and genomic data archiving. Nat Ecol Evol 8, 1224–1232 (2024).
17. Perrier, L., Blondal, E. & MacDonald, H. The views, perspectives, and experiences of academic researchers with data sharing and reuse: A meta-synthesis. PLoS ONE 15, e0229182 (2020).
18. Overture [Internet]. Software for big data genomic science. https://www.overture.bio/ (accessed 24 July 2024).
19. Overture stack [Internet]. Source code. https://github.com/overture-stack (accessed 25 July 2024).
20. ICGC Data Portal [Internet]. Available from: http://dcc.icgc.org (accessed 19 April 2024).
21. Zhang, J. et al. The International Cancer Genome Consortium Data Portal. Nat Biotechnol 37, 367–369 (2019).
22. Hartwig Medical Foundation. Database of metastatic cancer. https://www.hartwigmedicalfoundation.nl/en/data/database/ (accessed 25 July 2024).
23. Alonso, L. et al. TIGER: The gene expression regulatory variation landscape of human pancreatic islets. Cell Reports 37, 109807 (2021).
24. Shiell M, Bajari R, Andric D, Eubank J et al. (2025) Stage: React-based user interface framework to facilitate the creation of browser-accessible data portals (Version 1.0.1). [Computer software]. Software Heritage, https://archive.softwareheritage.org/swh:1:snp:84d73e6e53be322f8443db17958bedba6a7c4693;origin=https://github.com/overture-stack/stage
25. Shiell M, Bajari R, Andric D, Eubank J et al. (2025) Arranger - Data Portal API and UI component Generation (Version 2.12.3). [Computer software]. Software Heritage, https://archive.softwareheritage.org/swh:1:snp:d13c689513fb9e4dbf9978acc983b7251389ee52;origin=https://github.com/overture-stack/arranger
26. Keycloak (2025). Keycloak: Open Source Identity and Access Management For Modern Applications and Services (Version 26.1.3). [Computer software]. Software Heritage, https://archive.softwareheritage.org/swh:1:snp:b6c1f520311456de88d94437a080495ffbad78f7;origin=https://github.com/keycloak/keycloak

27. Shiell M, Bajari R, Andric D, Eubank J et al. (2025) Ego - Authentication and Authorization Microservice (Version 5.3.0). [Computer software]. Software Heritage, https://archive.softwareheritage.org/swh:1:snp:f34d155d92d46c1737da9293c6fb47af17898dfc;origin=https://github.com/overture-stack/ego
28. Shiell M, Bajari R, Andric D, Eubank J et al. (2025) SONG: Metadata management and automated validation system (Version 4.5.1). [Computer software]. Software Heritage, https://archive.softwareheritage.org/swh:1:snp:4527c299e4d0fa7167e30d901f58e25167b7b097;origin=https://github.com/overture-stack/SONG
29. Shiell M, Bajari R, Andric D, Eubank J et al. (2025) Score: Secure Cloud Object REsource -file transfer microservice (Version 5.11.0). [Computer software]. Software Heritage, https://archive.softwareheritage.org/swh:1:snp:ff210042483f6f25ab1408012b66c84816683863;origin=https://github.com/overture-stack/score
30. Shiell M, Bajari R, Andric D, Eubank J et al. (2025) Maestro: Indexing software to connect SONG and Arranger through Elasticsearch (Version 4.0.0). [Computer software]. Software Heritage, https://archive.softwareheritage.org/swh:1:snp:94dd1f9726979920c141c5f72e72424e4b71fd6d;origin=https://github.com/overture-stack/maestro
31. Elasticsearch [Internet]. Available from: https://www.elastic.co/ (accessed 25 July 2024).
32. GraphQL [Internet]. A query language for your API. Available from: https://graphql.org/ (accessed 25 July 2024).
33. International Cancer Genome Consortium Accelerating Research in Genomic Oncology. ICGC ARGO Data Platform. https://platform.icgc-argo.org/ (accessed 25 July 2024).
34. Kids First Data Resource Center. Kids First Data Resource Portal. https://portal.kidsfirstdrc.org/ (accessed 25 July 2024).
35. National Cancer Institute. Human Cancer Models Initiative (HCMI) Searchable Catalog. https://hcmi-searchable-catalog.nci.nih.gov/ (accessed 25 July 2024).
36. International Health Cohorts Consortium. IHCC Cohort Atlas. https://ihccglobal.org/cohort-atlas/ (accessed 25 July 2024).
37. VirusSeq Data Portal. https://virusseq-dataportal.ca/ (accessed 25 July 2024).
38. Gill, E. E. *et al.* The Canadian VirusSeq Data Portal & Duotang: open resources for SARS-CoV-2 viral sequences and genomic epidemiology. Preprint at https://doi.org/10.48550/ARXIV.2405.04734 (2024).
39. European-Canadian Cancer Network. EUCANCan Data Portal. https://eucancan.com/ (accessed 25 July 2024).
40. Ontario Hereditary Cancer Research Network. OHCRN Data Portal. https://ohcrn.ca/ (accessed 25 July 2024).
41. African Pathogen Data Sharing and Archive Platform. APA Portal. https://apaportal.sanbi.ac.za/ (accessed 25 July 2024).
42. Christoffels, A. et al. A pan-African pathogen genomics data sharing platform to support disease outbreaks. Nat Med 29, 1052–1055 (2023).
43. Docker Inc. Docker Compose. https://docs.docker.com/compose/ (accessed 25 July 2024).
44. HashiCorp. Terraform. https://www.terraform.io/ (accessed 25 July 2024).
45. The Linux Foundation. Helm. https://helm.sh/ (accessed 25 July 2024).
46. *The EU General Data Protection Regulation (GDPR): A Commentary*. (Oxford University PressNew York, 2020). doi:10.1093/oso/9780198826491.001.0001.
47. POPIA. Protection of Personal Information Act. [online] Available at: https://popia.co.za/ [Accessed 8 Aug. 2024].
48. ICGC-ARGO. ARGO Project. [online] Available at: https://www.icgc-argo.org/page/104/aegc [Accessed 8 Aug. 2024].
49. Global Alliance for Genomics and Health (GA4GH). Regulatory & Ethics Work Stream. [online] Available at: https://www.ga4gh.org/work_stream/regulatory-ethics/ [Accessed 8 Aug. 2024].

 Click here to access/download;Personal Cover;CoverLetter.pdf

OICR
Ontario Institute for Cancer Research

MaRS Centre
661 University Avenue, Suite 510
Toronto, ON M5G 0A3 Canada

Telephone 416-977-7599
Toll-free 1-866-678-6427
oicr.on.ca

Feb 28 2025
Qing Lan, PhD
Assistant Editor of GigaScience
GigaScience

Dear Dr. Lan,

Thank you for approving our manuscript (GIGAD2400541) for publication in GigaScience, contingent on minor revisions.

We thank the reviewers for their comments and suggestions for improving our manuscript. We have revised the document based on their input (shown as red text in the main document). We also provided detailed responses in the attached Response to reviewers letter. We trust the answers and changes incorporated here allow the manuscript to be formally accepted for publication in GigaScience and look forward to your positive reply.

Kind Regards,

Mélanie Courtot
Director, Genome Informatics and Principal Investigator
Assistant Professor, Medical Biophysics Department, University of Toronto
Assistant Professor, Department of Computer Science, University of Toronto
mcourtot@oicr.on.ca

Ontario Institute for Cancer Research
MaRS Centre, 661 University Avenue, Suite 510, Toronto, Ontario, Canada M5G 0A3
www.oicr.on.ca

Collaborate. Translate. Change lives.

Collaborate. Translate. Change lives.

Click here to access/download;Personal Cover;ResponseToReviewers.docx

Title: Response to Comments
Date: February 28th, 2025

Response to editor:

Prof Lan: In addition, please register any new software application in the bio.tools and SciCrunch.org databases to receive RRID (Research Resource Identification Initiative ID) and biotoolsID identifiers, and include these in your manuscript. Computational workflows should be registered in workflowhub.eu and the DOIs cited in the relevant places in the manuscript. These will facilitate tracking, reproducibility and re-use of your tool.

Response to Editor: Thank you for the feedback. We have registered the components as required and added the IDs in the manuscript, under Availability of supporting source code and requirements Section.

- RRID: SCR_026457
- Bio.tool ID: biotools:overture

Response to reviewers:

Reviewer #1: The authors present a new data platform, named Overture, for storing and browsing genomic dataset. In general, the paper is easy to read and nicely written, but it lacks major discussions on interoperability, heterogeneity management, and querying, which are not trivial when integrating genomic datasets at a large scale, as it is done in Overture. This is why I recommend a major revision, and detail it with the following points.

Response to Reviewer #1: Thank you for your careful review of the manuscript. We appreciate the time and effort you have dedicated to providing feedback, which has been valuable in improving our work. We have addressed your comments and suggestions in the manuscript and have outlined our responses below. We believe this feedback has improved both the content and presentation of our research, thank you.

Response to Major Comments:

1. I do not see a discussion on how to manage data heterogeneity, which is often present at several levels: different file formats (BAM, FASTQ, etc), different columns names, etc. I expected it when reading the sections data retrieval or data submission, and also to have it discussed in the discussion.

   The Overture suite supports upload and storage of any kind of data and data model. However the reviewer is correct that if the data hosted in Overture is heterogeneous, then discovery is made harder as it can't be presented in a unified/harmonized way. To address this, in the projects we manage, we typically require a data model be mapped to by the data submitters, which requires them to curate their data into the platform model. We have added text to that effect in the data submission and discussion sections, and mention some of the Overture extensions we are working on to alleviate this issue.

2. I did not find elements regarding the querying of the data. I understand this is managed by the Arranger component but I did not find details on how to query a single dataset, multiple datasets at the same time (c.f., my previous item on how to manage heterogeneity)

   Querying homogenous datasets are described in the first paragraph of our data retrieval section. As detailed above and in the updated text, when data across datasets is heterogeneous, they can be rendered into independent exploration pages. Because each data point is provided with a single unique ID in the backend, this could enable cross-querying between exploration pages. This is not yet supported in the UI, and because it is not on our medium-term roadmap further mention is out of scope for this paper. To enable querying across multiple datasets, these need to be harmonized against a shared data model.

3. I did not find either a discussion on how interoperability is managed in Overture. I see this sentence: "Overture Arranger provides shared discovery tools and interoperability with other international data portals.", but I would like to have more concrete proposals of how Overtures ensures interoperability.

   Data interoperability is thanks to the conformity of our data sets to defined schemas allowing systems to more easily be linked and interoperate. In the platforms we manage, dictionaries are carefully built to align with and reuse existing metadata standards where possible. For example, the ICGC-ARGO data model has been reused for MOHCCN (Marathon of Hope Cancer Centres Network) which means the data will be natively interoperable. We have updated the sentence pointed out by the reviewer to better reflect this. Technical interoperability is achieved when multiple Overture nodes are deployed within a single project - as described for EUCANCan in table 2 - which enables Maestro indexing to run across instances.

Response to Minor Revisions:

- Abstract : background and findings paragraphs to be justified
  - Updated accordingly
- Discussion: should be justified too
  - Updated accordingly
- Platform overview: Overture platforms are highly ... > Overture platform is highly …
  - Updated accordingly
- Data retrieval: the second paragraph could probably go before the Figure 2 image, next to the first paragraph to to loose so much space
  - Updated accordingly
- Impact: into three segments > two segments?
  - Updated accordingly
- Medium to small labs and institutions: the technical complexity ... have > has
  - Updated accordingly

Reviewer #2: This manuscript describes a data sharing system that can be utilized in various genome analysis and data sharing projects, ranging from large-scale genome analysis consortia to projects conducted by small to medium-sized research institutions. This system is based on the data portal and submission system of the International Cancer Genome Consortium (ICGC-DCC) and has been restructured into a microservice architecture to enable its use in other projects.

To promote further discoveries by facilitating the sharing and reuse of genomic data and metadata, it is essential that genomic data within online systems be properly maintained, searchable, retrievable, well-structured, and supportive of metadata and provenance tracking.

Research groups handling large-scale datasets that do not meet the requirements of existing archival resources such as GDC,SRA,EGA, or projects with datasets that do not conform to the specific data models or file formats accepted by these repositories, often face the need to build their own online data sharing systems. However, this is frequently a technical and financial challenge.

The system proposed by the authors is anticipated to be a highly effective solution, offering significant improvements in addressing these challenges while greatly enhancing accessibility, usability, and practicality for such projects.

The microservice architecture is both rationally and carefully designed, and the system is implemented using the well-established Java Spring Framework, ensuring robust security and reliability.

The manuscript's explanation of the system begins with a classification of the users who will utilize the system, and it follows a modern object-oriented development process. This approach makes the content highly comprehensible and serves as an exemplary model for similar works.

I believe this manuscript is all but ready for publication. However, I suggest adding explanations on a few points listed below, which would greatly enhance its utility for readers.

Response to Reviewer #2: We thank the reviewer for their positive feedback. We have addressed your comments and suggestions throughout the revised manuscript and outlined the changes below. Addressing these comments has definitely strengthened both the content and presentation of our research, thank you.

Response to Major Comments:

1. One of the key focuses of this paper is the transition to a microservice architecture, which represents a significant advancement in terms of scalability and potential for future feature expansion. Furthermore, the system is designed with high portability, not being tied to any specific cloud provider's infrastructure, and it appears to function even in on-premises environments. To aid readers in utilizing the Overture system, it would be extremely beneficial to include concrete examples of cloud IaaS configurations and on-premises hardware setups for deploying the system. For small projects, as already mentioned, deploying containers on a single server should suffice. However, for medium-to-large projects, it would be helpful to provide guidance on the intended hardware or cloud system configurations. Additionally, an estimate of the computational resources required for varying data sizes would be highly valuable to the readers.

We support users in configuring our software for production through our documentation and administration guides however we do not provide guidance on maintaining a production server due to both the rapidly evolving technological landscape and variability across deployment contexts.

While we can provide numbers based on the projects in table 2, those would only be anecdotal, which is why we instead describe the data size. Indeed, costs fluctuate based on the cloud or on-prem service provider, which would be the best to provide up-to-date cost estimates. We have added a sentence in “Availability of supporting source code and requirements” clarifying this.

2. Since the system is designed with federation in mind, and assumes long-distance communication across different jurisdictions, it would be helpful to include examples of federation setups across countries. These examples do not necessarily need to reflect existing implementations, but it would be valuable to describe the anticipated hardware or cloud configurations for such a setup.

   EUCANCan described in table 2 was our first attempt at international federation. We shared only a subset of the metadata for discovery in a central node, while the genomic data remained in place at each host institution. For ICGC ARGO we are planning to provide ‘true federation’ where neither the metadata nor data move from their host institution, and rather the querying is done at each node. In both cases the hardware and cloud configuration required is (1) not necessarily different from other nodes and (2) dependent on each host institution's capabilities. As the reviewer suggests, and pending the outcome of the ethical review we mention in the discussion section, there may be a future need to adopt different cloud configurations, such as requiring geolocation of the server hosting the data. Should this be the case, this would be managed by the hosting provider rather than the Overture suite.

3. When applying this system to actual projects, it would be helpful for readers if the manuscript discussed efforts or strategies, such as training or education, to encourage adoption by data curators and resource managers, enabling them to accept and start using the system effectively.

   Thank you for this recommendation. We wholeheartedly agree, and indeed since the submission of this paper we have extensively overhauled our documentation and provided direct support to users as well as provide a new online discussion forum. Based on their empirical feedback, our platform's approachability, defined by the initial ease of local setup, available documentation and support has emerged as a key differentiator from our competitors. We have added text in the discussion section to highlight that this is still work in progress.

Reviewer #3: The authors present an open-source data platform that provides a generic framework to facilitate the development and deployment of genomics data applications. This software has been designed to address the growing need for data platforms tailored to the custom and diverse requirements of various scientific projects. The publication is well-structured and effectively describes the different components of Overture, making it accessible to the target audience.

As highlighted in the introduction, the increasing diversity of genomics-based projects and datasets is not yet matched by adequate software solutions for storing and sharing such data online. Challenges such as the development costs of these platforms and the short-term funding of projects often hinder the creation,

reliability, and sustainability of resources. The authors tackle these issues with a modular solution, organized into configurable components.

This solution is distributed under open-source terms, and significant effort has been invested in providing extensive documentation, with commendable results. Additionally, the availability of a Docker Compose-based setup facilitates minimal local deployments, enabling new users to quickly explore the platform. These aspects demonstrate a strong commitment to usability and accessibility, enhancing the potential impact of the platform.

Response to Reviewer #3: Thank you for your positive feedback. We believe these changes outlined within our paper and below have substantially improved the manuscript's clarity and completeness. We thank the reviewer for their valuable feedback that has helped enhance the paper's quality.

Response to Major Comments

1. One of the notable strengths of Overture is its modularity, as demonstrated by the diverse component combinations outlined in Table 2, "Impact on consortium-level projects." While the ICGC ARGO project, which utilizes all components, is elaborated on in detail, it would be valuable to hear more about projects that employ only one component. Such use cases could further illustrate the platform's flexibility compared to more monolithic solutions.

   For this paper we prioritized showcasing the diversity of use cases in Table 2 as well as the complexity of ICGC ARGO vs going in depth for a restricted number of applications.Most projects from Table 2 are however associated with a more comprehensive case study description in our documentation and we have now added the respective hyperlinks to Table 2, thanks for the suggestion.

2. The manuscript mentions Keycloak and Ego as authentication and authorization components. Keycloak is a third-party service, while Ego is a custom component. However, the distinction between the two is not clearly addressed. Are these options fully interchangeable? What are their respective properties, and under which criteria might one be chosen over the other for local deployments? A discussion of these aspects would enhance clarity.

   We have added text in the Discussion section explaining the current support of both and future plans.

3. Another area that could benefit from elaboration is data submission from the user's perspective. While the manuscript provides a comprehensive description of the user interface for browsing and searching data, it minimally discusses the tools or processes for data submission. This is a critical aspect, as submission workflows often present significant challenges. Based on the online documentation, new components such as Lyric and Lectern are under development to assist with metadata model design and submission. Additionally, metadata submission via a command-line interface is already supported. Including a discussion of the existing command-line tools and ongoing developments in the manuscript's conclusion would provide a more complete picture.

   As noted by the reviewer, we do have some components under development to support submission. As those are not established and production ready yet, we purposely decide not to include them under Methods. However, we do appreciate the point made by the reviewer and the

usefulness of mentioning this work in progress, and have consequently added some text to the Discussion section.

Response to Minor Revisions:

- **Background**: The authors highlight the challenges of sharing data that do not conform to the specific data models and formats of major international platforms. While the critique of ad hoc solutions like supplementary files is valid, mention could be made of generic data publication platforms such as Zenodo or Figshare. These platforms, while not as advanced as the one described here, offer improved findability and citability compared to supplementary materials.
    - We've updated the background to mention " general-purpose repositories such as Zenodo and Figshare" highlighting how they "provide valuable platforms for research data publication".
- **Figure 1:** Consider renaming the figure to "Platform Components Overview" for clarity. From the caption, it is not immediately evident that the entities listed represent the platform's components.
    - We've updated figure 1 accordingly
- **Table 1:** While both Keycloak and Ego are mentioned as authentication/authorization services in the text, only Ego appears in Table 1. Clarifying the differences between these components and providing guidance on their selection would improve this section.
    - Overture is made to be highly modular, KeyCloak is a third-party open-source identity and access management service that can be used in place of Ego. We've updated the description and table 1 content to reflect this information.
- **Data Submission:** The statement "All publication controls are facilitated by Song" could be expanded with specific examples of controls implemented on existing deployments to illustrate this point more concretely.
    - As described in this section Songs publications controls include the commands "publish, unpublish and suppress". We've appended the sentence to read as follows "All publication controls are facilitated by Song using its publication command or endpoint."
- **Data Administration:** The phrase "outlining the structure and syntax of the data model in JSON format" could be revised for precision. Replacing "outlining" with "describing" or "specifying" better reflects the role of JSON schemas in providing detailed definitions of data models.
    - We have updated "outlining" to "specifying"
- **Table 2:** The component referred to as the "Overture Data Management System (DMS)" in the EUCANCan project is not clearly identified elsewhere in the manuscript. Is this equivalent to Song or another component? Clarification would be helpful (Ego, Song, Score, Maestro, Arranger).
    - The DMS included all core Overture services packaged with automated scripts for server deployments. For various reasons such as lack of scalability and configuration this Overture package was discontinued and has now been replaced with our more flexible docker setups. We have updated the components used to reflect all the services deployed rather than mentioning the DMS.
- **"Medium to small laboratories and institutions":** In the phrase "a Docker Compose that enables users to deploy…," consider replacing "a Docker Compose" with "a Docker Compose-based makefile" for greater technical accuracy (and to drive readers to use the Makefile rather than the more complicated docker-compose).
    - We have updated the terminology to "Docker Compose-based makefile"
