# Supplementary material for: Overture: an open-source genomics data platform
Source: Gigascience. 2025 Apr 24;14:giaf038. doi: 10.1093/gigascience/giaf038 (PMC12020472; doi:10.1093/gigascience/giaf038)
Supplement: giaf038_Response_to_Reviewer_Comments_Original_Submission [file giaf038_response_to_reviewer_comments_original_submission.pdf]

Author's Response To Reviewer Comments

Please refer to attached Response to Reviewers letter - content copied below for reference

Response to editor:

Prof Lan: In addition, please register any new software application in the bio.tools and SciCrunch.org databases to receive RRID (Research Resource Identification Initiative ID) and biotoolsID identifiers, and include these in your manuscript. Computational workflows should be registered in workflowhub.eu and the DOIs cited in the relevant places in the manuscript. These will facilitate tracking, reproducibility and re-use of your tool.

Response to Editor: Thank you for the feedback. We have registered the components as required and added the IDs in the manuscript, under Availability of supporting source code and requirements Section.

· RRID: SCR_026457
· Bio.tool ID: biotools:overture

Response to reviewers:

Reviewer #1: The authors present a new data platform, named Overture, for storing and browsing genomic dataset. In general, the paper is easy to read and nicely written, but it lacks major discussions on interoperability, heterogeneity management, and querying, which are not trivial when integrating genomic datasets at a large scale, as it is done in Overture. This is why I recommend a major revision, and detail it with the following points.

Response to Reviewer #1: Thank you for your careful review of the manuscript. We appreciate the time and effort you have dedicated to providing feedback, which has been valuable in improving our work. We have addressed your comments and suggestions in the manuscript and have outlined our responses below. We believe this feedback has improved both the content and presentation of our research, thank you.

Response to Major Comments:

1. I do not see a discussion on how to manage data heterogeneity, which is often present at several levels: different file formats (BAM, FASTQ, etc), different columns names, etc. I expected it when reading the sections data retrieval or data submission, and also to have it discussed in the discussion.

The Overture suite supports upload and storage of any kind of data and data model. However the reviewer is correct that if the data hosted in Overture is heterogeneous, then discovery is made harder as it can't be presented in a unified/harmonized way. To address this, in the projects we manage, we typically require a data model be mapped to by the data submitters, which requires them to curate their data into the platform model. We have added text to that effect in the data submission and discussion sections, and mention some of the Overture extensions we are working on to alleviate this issue.

2. I did not find elements regarding the querying of the data. I understand this is managed by the Arranger component but I did not find details on how to query a single dataset, multiple datasets at the same time (c.f., my previous item on how to manage heterogeneity)

Querying homogenous datasets are described in the first paragraph of our data retrieval section. As detailed above and in the updated text, when data across datasets is heterogeneous, they can be rendered into independent exploration pages. Because each data point is provided with a single unique ID in the backend, this could enable cross-querying between exploration pages. This is not yet supported in the UI, and because it is not on our medium-term roadmap further mention is out of scope for this paper. To

enable querying across multiple datasets, these need to be harmonized against a shared data model.

3. I did not find either a discussion on how interoperability is managed in Overture. I see this sentence: "Overture Arranger provides shared discovery tools and interoperability with other international data portals.", but I would like to have more concrete proposals of how Overtures ensures interoperability.

Data interoperability is thanks to the conformity of our data sets to defined schemas allowing systems to more easily be linked and interoperate. In the platforms we manage, dictionaries are carefully built to align with and reuse existing metadata standards where possible. For example, the ICGC-ARGO data model has been reused for MOHCCN (Marathon of Hope Cancer Centres Network) which means the data will be natively interoperable. We have updated the sentence pointed out by the reviewer to better reflect this. Technical interoperability is achieved when multiple Overture nodes are deployed within a single project - as described for EUCANCan in table 2 - which enables Maestro indexing to run across instances.

Response to Minor Revisions:

● Abstract : background and findings paragraphs to be justified
○ Updated accordingly
● Discussion: should be justified too
○ Updated accordingly
● Platform overview: Overture platforms are highly ... > Overture platform is highly …
○ Updated accordingly
● Data retrieval: the second paragraph could probably go before the Figure 2 image, next to the first paragraph to to loose so much space
○ Updated accordingly
● Impact: into three segments > two segments?
○ Updated accordingly
● Medium to small labs and institutions: the technical complexity ... have > has
○ Updated accordingly

Reviewer #2: This manuscript describes a data sharing system that can be utilized in various genome analysis and data sharing projects, ranging from large-scale genome analysis consortia to projects conducted by small to medium-sized research institutions. This system is based on the data portal and submission system of the International Cancer Genome Consortium (ICGC-DCC) and has been restructured into a microservice architecture to enable its use in other projects.

To promote further discoveries by facilitating the sharing and reuse of genomic data and metadata, it is essential that genomic data within online systems be properly maintained, searchable, retrievable, well-structured, and supportive of metadata and provenance tracking.

Research groups handling large-scale datasets that do not meet the requirements of existing archival resources such as GDC,SRA,EGA, or projects with datasets that do not conform to the specific data models or file formats accepted by these repositories, often face the need to build their own online data sharing systems. However, this is frequently a technical and financial challenge.

The system proposed by the authors is anticipated to be a highly effective solution, offering significant improvements in addressing these challenges while greatly enhancing accessibility, usability, and practicality for such projects.

The microservice architecture is both rationally and carefully designed, and the system is implemented using the well-established Java Spring Framework, ensuring robust security and reliability.

The manuscript's explanation of the system begins with a classification of the users who will utilize the system, and it follows a modern object-oriented development process. This approach makes the content highly comprehensible and serves as an exemplary model for similar works.

I believe this manuscript is all but ready for publication. However, I suggest adding explanations on a few points listed below, which would greatly enhance its utility for readers.

Response to Reviewer #2: We thank the reviewer for their positive feedback. We have addressed your comments and suggestions throughout the revised manuscript and outlined the changes below. Addressing these comments has definitely strengthened both the content and presentation of our research, thank you.

Response to Major Comments:

1. One of the key focuses of this paper is the transition to a microservice architecture, which represents a significant advancement in terms of scalability and potential for future feature expansion. Furthermore, the system is designed with high portability, not being tied to any specific cloud provider's infrastructure, and it appears to function even in on-premises environments. To aid readers in utilizing the Overture system, it would be extremely beneficial to include concrete examples of cloud IaaS configurations and on-premises hardware setups for deploying the system. For small projects, as already mentioned, deploying containers on a single server should suffice. However, for medium-to-large projects, it would be helpful to provide guidance on the intended hardware or cloud system configurations. Additionally, an estimate of the computational resources required for varying data sizes would be highly valuable to the readers.

We support users in configuring our software for production through our documentation and administration guides however we do not provide guidance on maintaining a production server due to both the rapidly evolving technological landscape and variability across deployment contexts.

While we can provide numbers based on the projects in table 2, those would only be anecdotal, which is why we instead describe the data size. Indeed, costs fluctuate based on the cloud or on-prem service provider, which would be the best to provide up-to-date cost estimates. We have added a sentence in “Availability of supporting source code and requirements” clarifying this.

2. Since the system is designed with federation in mind, and assumes long-distance communication across different jurisdictions, it would be helpful to include examples of federation setups across countries. These examples do not necessarily need to reflect existing implementations, but it would be valuable to describe the anticipated hardware or cloud configurations for such a setup.

EUCANCan described in table 2 was our first attempt at international federation. We shared only a subset of the metadata for discovery in a central node, while the genomic data remained in place at each host institution. For ICGC ARGO we are planning to provide ‘true federation’ where neither the metadata nor data move from their host institution, and rather the querying is done at each node. In both cases the hardware and cloud configuration required is (1) not necessarily different from other nodes and (2) dependent on each host institution's capabilities. As the reviewer suggests, and pending the outcome of the ethical review we mention in the discussion section, there may be a future need to adopt different cloud configurations, such as requiring geolocation of the server hosting the data. Should this be the case, this would be managed by the hosting provider rather than the Overture suite.

3. When applying this system to actual projects, it would be helpful for readers if the manuscript discussed efforts or strategies, such as training or education, to encourage adoption by data curators and resource managers, enabling them to accept and start using the system effectively.

Thank you for this recommendation. We wholeheartedly agree, and indeed since the submission of this paper we have extensively overhauled our documentation and provided direct support to users as well as provide a new online discussion forum. Based on their empirical feedback, our platform's approachability, defined by the initial ease of local setup, available documentation and support has emerged as a key differentiator from our competitors. We have added text in the discussion section to highlight that this is

still work in progress.

Reviewer #3: The authors present an open-source data platform that provides a generic framework to facilitate the development and deployment of genomics data applications. This software has been designed to address the growing need for data platforms tailored to the custom and diverse requirements of various scientific projects. The publication is well-structured and effectively describes the different components of Overture, making it accessible to the target audience.

As highlighted in the introduction, the increasing diversity of genomics-based projects and datasets is not yet matched by adequate software solutions for storing and sharing such data online. Challenges such as the development costs of these platforms and the short-term funding of projects often hinder the creation, reliability, and sustainability of resources. The authors tackle these issues with a modular solution, organized into configurable components.

This solution is distributed under open-source terms, and significant effort has been invested in providing extensive documentation, with commendable results. Additionally, the availability of a Docker Compose-based setup facilitates minimal local deployments, enabling new users to quickly explore the platform. These aspects demonstrate a strong commitment to usability and accessibility, enhancing the potential impact of the platform.

Response to Reviewer #3: Thank you for your positive feedback. We believe these changes outlined within our paper and below have substantially improved the manuscript's clarity and completeness. We thank the reviewer for their valuable feedback that has helped enhance the paper's quality.

Response to Major Comments

1. One of the notable strengths of Overture is its modularity, as demonstrated by the diverse component combinations outlined in Table 2, "Impact on consortium-level projects." While the ICGC ARGO project, which utilizes all components, is elaborated on in detail, it would be valuable to hear more about projects that employ only one component. Such use cases could further illustrate the platform's flexibility compared to more monolithic solutions.

For this paper we prioritized showcasing the diversity of use cases in Table 2 as well as the complexity of ICGC ARGO vs going in depth for a restricted number of applications.Most projects from Table 2 are however associated with a more comprehensive case study description in our documentation and we have now added the respective hyperlinks to Table 2, thanks for the suggestion.

2. The manuscript mentions Keycloak and Ego as authentication and authorization components. Keycloak is a third-party service, while Ego is a custom component. However, the distinction between the two is not clearly addressed. Are these options fully interchangeable? What are their respective properties, and under which criteria might one be chosen over the other for local deployments? A discussion of these aspects would enhance clarity.

We have added text in the Discussion section explaining the current support of both and future plans.

3. Another area that could benefit from elaboration is data submission from the user's perspective. While the manuscript provides a comprehensive description of the user interface for browsing and searching data, it minimally discusses the tools or processes for data submission. This is a critical aspect, as submission workflows often present significant challenges. Based on the online documentation, new components such as Lyric and Lectern are under development to assist with metadata model design and submission. Additionally, metadata submission via a command-line interface is already supported. Including a discussion of the existing command-line tools and ongoing developments in the manuscript's conclusion would provide a more complete picture.

As noted by the reviewer, we do have some components under development to support submission. As those are not established and production ready yet, we purposely decide not to include them under Methods. However, we do appreciate the point made by the reviewer and the usefulness of mentioning this work in progress, and have consequently added some text to the Discussion section.

Response to Minor Revisions:

- Background: The authors highlight the challenges of sharing data that do not conform to the specific data models and formats of major international platforms. While the critique of ad hoc solutions like supplementary files is valid, mention could be made of generic data publication platforms such as Zenodo or Figshare. These platforms, while not as advanced as the one described here, offer improved findability and citability compared to supplementary materials.
  - We've updated the background to mention " general-purpose repositories such as Zenodo and Figshare" highlighting how they "provide valuable platforms for research data publication".
- Figure 1: Consider renaming the figure to "Platform Components Overview" for clarity. From the caption, it is not immediately evident that the entities listed represent the platform's components.
  - We've updated figure 1 accordingly
- Table 1: While both Keycloak and Ego are mentioned as authentication/authorization services in the text, only Ego appears in Table 1. Clarifying the differences between these components and providing guidance on their selection would improve this section.
  - Overture is made to be highly modular, KeyCloak is a third-party open-source identity and access management service that can be used in place of Ego. We've updated the description and table 1 content to reflect this information.
- Data Submission: The statement "All publication controls are facilitated by Song" could be expanded with specific examples of controls implemented on existing deployments to illustrate this point more concretely.
  - As described in this section Songs publications controls include the commands "publish, unpublish and suppress". We've appended the sentence to read as follows "All publication controls are facilitated by Song using its publication command or endpoint."
- Data Administration: The phrase "outlining the structure and syntax of the data model in JSON format" could be revised for precision. Replacing "outlining" with "describing" or "specifying" better reflects the role of JSON schemas in providing detailed definitions of data models.
  - We have updated "outlining" to "specifying"
- Table 2: The component referred to as the "Overture Data Management System (DMS)" in the EUCANCan project is not clearly identified elsewhere in the manuscript. Is this equivalent to Song or another component? Clarification would be helpful (Ego, Song, Score, Maestro, Arranger).
  - The DMS included all core Overture services packaged with automated scripts for server deployments. For various reasons such as lack of scalability and configuration this Overture package was discontinued and has now been replaced with our more flexible docker setups. We have updated the components used to reflect all the services deployed rather than mentioning the DMS.
- "Medium to small laboratories and institutions": In the phrase "a Docker Compose that enables users to deploy…," consider replacing "a Docker Compose" with "a Docker Compose-based makefile" for greater technical accuracy (and to drive readers to use the Makefile rather than the more complicated docker-compose).
  - We have updated the terminology to "Docker Compose-based makefile"
