# Supplementary material for: Overture: an open-source genomics data platform
Source: Gigascience. 2025 Apr 24;14:giaf038. doi: 10.1093/gigascience/giaf038 (PMC12020472; doi:10.1093/gigascience/giaf038)
Supplement: giaf038_Reviewer_1_Report_Original_Submission — Nelly Barret -- 12/23/2024 [file giaf038_reviewer_1_report_original_submission.pdf]

Reviewer Report

Title: Overture: An Open-Source Genomics Data Platform

Version: Original Submission **Date:** 12/23/2024

Reviewer name: Nelly Barret

Reviewer Comments to Author:

The authors present a new data platform, named Overture, for storing and browsing genomic dataset. In general, the paper is easy to read and nicely written, but it lacks major discussions on interoperability, heterogeneity management, and querying, which are not trivial when integrating genomic datasets at a large scale, as it is done in Overture.This is why I recommend a major revision, and detail it with the following points :- I do not see a discussion on how to manage data heterogeneity, which is often present at several levels: different file formats (BAM, FASTQ, etc), different columns names, etc. I expected it when reading the sections data retrieval or data submission, and also to have it discussed in the discussion.- I did not find elements regarding the querying of the data. I understand this is managed by the Arranger component but I did not find details on how to query a single dataset, multiple datasets at the same time (c.f., my previous item on how to manage heterogeneity)- I did not find either a discussion on how interoperability is managed in Overture. I see this sentence: "Overture Arranger provides shared discovery tools and interoperability with other international data portals.", but I would like to have more concrete proposals of how Overtures ensures interoperability.- Several typos/presentation failures :- Abstract : background and findings paragraphs to be justified- Discussion: should be justified too- Platform overview: Overture platforms are highly ... -> Overture platform is highly ...- Data retrieval: the second paragraph could probably go before the Figure 2 image, next to the first paragraph to to loose so much space- Impact: into three segments -> two segments?- Medium to small labs and institutions: the technical complexity ... have -> has

Level of Interest

Please indicate how interesting you found the manuscript: Choose an item.

Quality of Written English

Please indicate the quality of language in the manuscript: Choose an item.

Declaration of Competing Interests

Please complete a declaration of competing interests, considering the following questions:

- Have you in the past five years received reimbursements, fees, funding, or salary from an organisation that may in any way gain or lose financially from the publication of this manuscript, either now or in the future?

- Do you hold any stocks or shares in an organisation that may in any way gain or lose financially from the publication of this manuscript, either now or in the future?
- Do you hold or are you currently applying for any patents relating to the content of the manuscript?
- Have you received reimbursements, fees, funding, or salary from an organization that holds or has applied for patents relating to the content of the manuscript?
- Do you have any other financial competing interests?
- Do you have any non-financial competing interests in relation to this paper?

If you can answer no to all of the above, write 'I declare that I have no competing interests' below. If your reply is yes to any, please give details below.

I declare that I have no competing interests

I agree to the open peer review policy of the journal. I understand that my name will be included on my report to the authors and, if the manuscript is accepted for publication, my named report including any attachments I upload will be posted on the website along with the authors' responses. I agree for my report to be made available under an Open Access Creative Commons CC-BY license (http://creativecommons.org/licenses/by/4.0/). I understand that any comments which I do not wish to be included in my named report can be included as confidential comments to the editors, which will not be published.

Choose an item.

To further support our reviewers, we have joined with Publons, where you can gain additional credit to further highlight your hard work (see: https://publons.com/journal/530/gigascience). On publication of this paper, your review will be automatically added to Publons, you can then choose whether or not to claim your Publons credit. I understand this statement.

Yes Choose an item.
