# Supplementary material for: Overture: an open-source genomics data platform
Source: Gigascience. 2025 Apr 24;14:giaf038. doi: 10.1093/gigascience/giaf038 (PMC12020472; doi:10.1093/gigascience/giaf038)
Supplement: giaf038_Reviewer_2_Report_Original_Submission — Osamu Ogasawara -- 12/26/2024 [file giaf038_reviewer_2_report_original_submission.pdf]

Reviewer Report

Title: Overture: An Open-Source Genomics Data Platform

Version: Original Submission **Date:** 12/26/2024

Reviewer name: Osamu Ogasawara

Reviewer Comments to Author:

This manuscript describes a data sharing system that can be utilized in various genome analysis and data sharing projects, ranging from large-scale genome analysis consortia to projects conducted by small to medium-sized research institutions. This system is based on the data portal and submission system of the International Cancer Genome Consortium (ICGC-DCC) and has been restructured into a microservice architecture to enable its use in other projects.To promote further discoveries by facilitating the sharing and reuse of genomic data and metadata, it is essential that genomic data within online systems be properly maintained, searchable, retrievable, well-structured, and supportive of metadata and provenance tracking.Research groups handling large-scale datasets that do not meet the requirements of existing archival resources such as GDC,SRA,EGA, or projects with datasets that do not conform to the specific data models or file formats accepted by these repositories, often face the need to build their own online data sharing systems. However, this is frequently a technical and financial challenge.The system proposed by the authors is anticipated to be a highly effective solution, offering significant improvements in addressing these challenges while greatly enhancing accessibility, usability, and practicality for such projects.The microservice architecture is both rationally and carefully designed, and the system is implemented using the well-established Java Spring Framework, ensuring robust security and reliability.The manuscript's explanation of the system begins with a classification of the users who will utilize the system, and it follows a modern object-oriented development process. This approach makes the content highly comprehensible and serves as an exemplary model for similar works.I believe this manuscript is all but ready for publication. However, I suggest adding explanations on a few points listed below, which would greatly enhance its utility for readers.1. One of the key focuses of this paper is the transition to a microservice architecture, which represents a significant advancement in terms of scalability and potential for future feature expansion. Furthermore, the system is designed with high portability, not being tied to any specific cloud provider's infrastructure, and it appears to function even in on-premises environments.To aid readers in utilizing the Overture system, it would be extremely beneficial to include concrete examples of cloud IaaS configurations and on-premises hardware setups for deploying the system. For small projects, as already mentioned, deploying containers on a single server should suffice. However, for medium-to-large projects, it would be helpful to provide guidance on the intended hardware or cloud system configurations. Additionally, an estimate of the computational resources required for varying data sizes would be highly valuable to the readers.2. Since the system is designed with federation in mind, and assumes long-distance communication across different jurisdictions, it would be helpful to include examples of federation setups across countries. These examples do not necessarily need to reflect existing implementations, but it would be valuable to describe the anticipated hardware or cloud configurations for such a setup.3. When applying this system

to actual projects, it would be helpful for readers if the manuscript discussed efforts or strategies, such as training or education, to encourage adoption by data curators and resource managers, enabling them to accept and start using the system effectively.

Level of Interest

Please indicate how interesting you found the manuscript: Choose an item.

Quality of Written English

Please indicate the quality of language in the manuscript: Choose an item.

Declaration of Competing Interests

Please complete a declaration of competing interests, considering the following questions:

- Have you in the past five years received reimbursements, fees, funding, or salary from an organisation that may in any way gain or lose financially from the publication of this manuscript, either now or in the future?
- Do you hold any stocks or shares in an organisation that may in any way gain or lose financially from the publication of this manuscript, either now or in the future?
- Do you hold or are you currently applying for any patents relating to the content of the manuscript?
- Have you received reimbursements, fees, funding, or salary from an organization that holds or has applied for patents relating to the content of the manuscript?
- Do you have any other financial competing interests?
- Do you have any non-financial competing interests in relation to this paper?

If you can answer no to all of the above, write 'I declare that I have no competing interests' below. If your reply is yes to any, please give details below.

I declare that I have no competing interests.

I agree to the open peer review policy of the journal. I understand that my name will be included on my report to the authors and, if the manuscript is accepted for publication, my named report including any attachments I upload will be posted on the website along with the authors' responses. I agree for my report to be made available under an Open Access Creative Commons CC-BY license (http://creativecommons.org/licenses/by/4.0/). I understand that any comments which I do not wish to be included in my named report can be included as confidential comments to the editors, which will not be published.

Choose an item.

To further support our reviewers, we have joined with Publons, where you can gain additional credit to further highlight your hard work (see: https://publons.com/journal/530/gigascience). On publication of this paper, your review will be automatically added to Publons, you can then choose whether or not to claim your Publons credit. I understand this statement.

Yes Choose an item.
