# Supplementary material for: Overture: an open-source genomics data platform
Source: Gigascience. 2025 Apr 24;14:giaf038. doi: 10.1093/gigascience/giaf038 (PMC12020472; doi:10.1093/gigascience/giaf038)
Supplement: giaf038_Reviewer_3_Report_Original_Submission — HervÃ© MÃ©nager -- 1/2/2025 [file giaf038_reviewer_3_report_original_submission.pdf]

Reviewer Report

Title: Overture: An Open-Source Genomics Data Platform

Version: Original Submission **Date:** 1/2/2025

Reviewer name: HervÃ© MÃ©nager

Reviewer Comments to Author:

The authors present an open-source data platform that provides a generic framework to facilitate the development and deployment of genomics data applications. This software has been designed to address the growing need for data platforms tailored to the custom and diverse requirements of various scientific projects. The publication is well-structured and effectively describes the different components of Overture, making it accessible to the target audience.As highlighted in the introduction, the increasing diversity of genomics-based projects and datasets is not yet matched by adequate software solutions for storing and sharing such data online. Challenges such as the development costs of these platforms and the short-term funding of projects often hinder the creation, reliability, and sustainability of resources. The authors tackle these issues with a modular solution, organized into configurable components.This solution is distributed under open-source terms, and significant effort has been invested in providing extensive documentation, with commendable results. Additionally, the availability of a Docker Compose-based setup facilitates minimal local deployments, enabling new users to quickly explore the platform. These aspects demonstrate a strong commitment to usability and accessibility, enhancing the potential impact of the platform.**Major Comments**- One of the notable strengths of Overture is its modularity, as demonstrated by the diverse component combinations outlined in Table 2, "Impact on consortium-level projects." While the ICGC ARGO project, which utilizes all components, is elaborated on in detail, it would be valuable to hear more about projects that employ only one component. Such use cases could further illustrate the platform's flexibility compared to more monolithic solutions.- The manuscript mentions Keycloak and Ego as authentication and authorization components. Keycloak is a third-party service, while Ego is a custom component. However, the distinction between the two is not clearly addressed. Are these options fully interchangeable? What are their respective properties, and under which criteria might one be chosen over the other for local deployments? A discussion of these aspects would enhance clarity.- Another area that could benefit from elaboration is data submission from the user's perspective. While the manuscript provides a comprehensive description of the user interface for browsing and searching data, it minimally discusses the tools or processes for data submission. This is a critical aspect, as submission workflows often present significant challenges. Based on the online documentation, new components such as Lyric and Lectern are under development to assist with metadata model design and submission. Additionally, metadata submission via a command-line interface is already supported. Including a discussion of the existing command-line tools and ongoing developments in the manuscript's conclusion would provide a more complete picture.**Minor Points**- **Background:** The authors highlight the challenges of sharing data that do not conform to the specific data models and formats of major international platforms. While the critique of ad hoc solutions like supplementary files is valid, mention could be

made of generic data publication platforms such as Zenodo or Figshare. These platforms, while not as advanced as the one described here, offer improved findability and citability compared to supplementary materials.- **Figure 1:** Consider renaming the figure to "Platform Components Overview" for clarity. From the caption, it is not immediately evident that the entities listed represent the platform's components.- **Table 1:** While both Keycloak and Ego are mentioned as authentication/authorization services in the text, only Ego appears in Table 1. Clarifying the differences between these components and providing guidance on their selection would improve this section.- **Data Submission:** The statement "All publication controls are facilitated by Song" could be expanded with specific examples of controls implemented on existing deployments to illustrate this point more concretely.- **Data Administration:** The phrase "outlining the structure and syntax of the data model in JSON format" could be revised for precision. Replacing "outlining" with "describing" or "specifying" better reflects the role of JSON schemas in providing detailed definitions of data models.- **Table 2:** The component referred to as the "Overture Data Management System (DMS)" in the EUCANCan project is not clearly identified elsewhere in the manuscript. Is this equivalent to Song or another component? Clarification would be helpful.- **"Medium to small laboratories and institutions":** In the phrase "a Docker Compose that enables users to deploy…," consider replacing "a Docker Compose" with "a Docker Compose-based makefile" for greater technical accuracy (and to drive readers to use the Makefile rather than the more complicated docker-compose).

Level of Interest

Please indicate how interesting you found the manuscript: Choose an item.

Quality of Written English

Please indicate the quality of language in the manuscript: Choose an item.

Declaration of Competing Interests

Please complete a declaration of competing interests, considering the following questions:

- Have you in the past five years received reimbursements, fees, funding, or salary from an organisation that may in any way gain or lose financially from the publication of this manuscript, either now or in the future?
- Do you hold any stocks or shares in an organisation that may in any way gain or lose financially from the publication of this manuscript, either now or in the future?
- Do you hold or are you currently applying for any patents relating to the content of the manuscript?
- Have you received reimbursements, fees, funding, or salary from an organization that holds or has applied for patents relating to the content of the manuscript?
- Do you have any other financial competing interests?
- Do you have any non-financial competing interests in relation to this paper?

If you can answer no to all of the above, write 'I declare that I have no competing interests' below. If your reply is yes to any, please give details below.

I declare that I have no competing interests

I agree to the open peer review policy of the journal. I understand that my name will be included on my report to the authors and, if the manuscript is accepted for publication, my named report including any attachments I upload will be posted on the website along with the authors' responses. I agree for my report to be made available under an Open Access Creative Commons CC-BY license (http://creativecommons.org/licenses/by/4.0/). I understand that any comments which I do not wish to be included in my named report can be included as confidential comments to the editors, which will not be published.

Choose an item.

To further support our reviewers, we have joined with Publons, where you can gain additional credit to further highlight your hard work (see: https://publons.com/journal/530/gigascience). On publication of this paper, your review will be automatically added to Publons, you can then choose whether or not to claim your Publons credit. I understand this statement.

Yes Choose an item.
